# Supplementary material for: Genome assembly of Musa beccarii shows extensive chromosomal rearrangements and genome expansion during evolution of Musaceae genomes
Source: Gigascience. 2023 Feb 21;12:giad005. doi: 10.1093/gigascience/giad005 (PMC9941839; doi:10.1093/gigascience/giad005)
Supplement: giad005_GIGA-D-22-00219_Revision_1 [file giad005_giga-d-22-00219_revision_1.pdf]

GigaScience

Genome assembly of Musa beccarii shows extensive chromosomal rearrangements and genome expansion during evolution of Musaceae genomes

--Manuscript Draft--

**Manuscript Number:**	GIGA-D-22-00219R1
**Full Title:**	Genome assembly of Musa beccarii shows extensive chromosomal rearrangements and genome expansion during evolution of Musaceae genomes
**Article Type:**	Data Note
**Funding Information:**	
**Abstract:**	Background: Musa beccarii (Musaceae) is a species of banana native to Borneo, sometimes grown as an ornamental. Other Musa species have a basic chromosome number of x=7, 10 or 11, but M. beccarii is the only species with x=9 (2n=2x=18) in the genus, a number shared with the sister genera Ensete and Musella. M. beccarii is in section Callimusa of the genus, sister to section Musa which includes currently assembled genomes. To better understand genome evolution and diversity in Musaceae, we aimed to generate a high-quality chromosome-scale genome assembly of M. beccarii.<br>Findings: The M. beccarii genome was assembled by long read and Hi-C sequencing, with gene annotations using both long Iso-Seq and short RNA-seq reads. M. beccarii displayed the largest assembly size, ~570 Mbp, among known Musaceae assemblies, attributed to transposable element expansion and amplification of 45S rDNA sites. We detected extensive genome-wide chromosome fusions and fissions between M. beccarii and the other Musa and Ensete species by synteny analysis, far beyond those expected from chromosome number differences. Within Musaceae, M. beccarii showed a reduced number of terpenoid synthase genes which are related to chemical defense and enrichment in lipid metabolism genes linked to the physical defense of the cell wall. Furthermore, type III polyketide synthase (T3PKS) was the most abundant biosynthetic gene cluster in M. beccarii with no conservation in the other Musaceae genomes.<br>Conclusions: M. beccarii, as the first chromosome scale genome assembly sequenced in the Callimusa section in Musa, provides an important resource for understanding evolutionary mechanisms in the Musaceae and builds knowledge of the pangenome.
**Corresponding Author:**	Xue-Jun Ge<br>South China Botanical Garden<br>Guangzhou, Guangdong CHINA
**Corresponding Author Secondary Information:**	
**Corresponding Author's Institution:**	South China Botanical Garden
**Corresponding Author's Secondary Institution:**	
**First Author:**	Zheng-Feng Wang
**First Author Secondary Information:**	
**Order of Authors:**	Zheng-Feng Wang
	Mathieu Rouard
	Gaetan Droc
	Pat (J.S.) Heslop-Harrison
	Xue-Jun Ge
**Order of Authors Secondary Information:**	
**Response to Reviewers:**	December 24, 2022<br>GigaScience

Powered by Editorial Manager® and ProduXion Manager® from Aries Systems Corporation

Dear editor,

Thank you very much for your decision letter about our manuscript entitled “Genome assembly of Musa beccarii shows extensive chromosomal rearrangements and genome expansion during evolution of Musaceae genomes”(No. GIGA-D-22-00219), including the comments.

We are now sending our responses to comments with our revised manuscript (both tracked and clean versions). All the modified parts in the revised manuscript are marked in red.

Our specific responses are as follows:

Reviewer(s)' Comments to Author:

Reviewer: 1

Wang et al. describe the genome sequence of Musa beccarii. The assembly is presented and compared against existing genome sequences. This additional genome sequence provides further insights into the evolution of Musaceae. All data sets were submitted to the corresponding repositories and will be shared via the banana genome hub. This work is certainly of interest to the community and the availability of all data sets will facilitate reuse in future studies. While the data set is of high quality, there are some (technical) issues that should be addressed. For example, the statements about the genome size should be checked and additional evidence for the contraction/expansion event would be helpful. Please find a list of specific comments below.

1) There is a difference between genome (DNA) and a genome sequence (information). This is particularly relevant when the genome size does not match the assembly size. Please adjust the use of both terms throughout the manuscript.

>>>We now replaced “genome” with “assembly” or “assembled genome” in the revised manuscript as suggested.

2) Do the authors have an approval from Malaysia to use this material for research and to publish genomic and transcriptomic data sets?

>>> Musa beccarii was named as a new species by Prof. Simmonds in 1960 based on a cultivated plant in Trinidad. M. beccarii was commonly cultivated in many botanical gardens across the world. Our material was retrieved from ex situ collection hosted in one European botanical garden, not directly from the wild, which arrived in Europe before 1993 and for which we got a transfer agreement. The material was used only for scientific research, not commercial purpose. Based on the non-retroactive principle of "Convention on Biological Diversity", this material was not constrained by the International Treaty on Plant Genetic Resources for Food and Agriculture.

Therefore, since the material was collected before Malaysia adopted a national ABS law, there is no need to get approval from Malaysia to use this material for research and to publish genomic and transcriptomic data sets. We now delete the sentence “The origin of this species was one botanical garden at Sabah, Borneo, Malaysia.” and briefly include above information in “Ethics Approval and Consent to Participate” section. Please see lines 691-692.

3) line 113: PacBio is not able to sequence RNAs. ONT is the only technology for high throughout RNA sequencing. What the authors are trying to describe is probably a cDNA sequencing run (with HiFi?). The short reads are most likely also derived from cDNAs.

>>>We agree with the reviewer. We have clarified it. Please see line 113-114 as follows: “total RNA from M. beccarii leaves of the same individual were extracted and reverse-transcribed to cDNA for PacBio full-length RNA transcripts sequencing (Iso-Seq)”.

4) Details of the conditions and time points of RNA extraction need to be added. Almost nothing is written about the samples used for the sequencing experiments. Please make sure to update the metadata (growth condition, plant part, developmental stage, ...) accordingly.

>>>We have included the information and updated the metadata of our submission to the GenBank (Biosample attributes tables). Please see lines 104-106. We deposited GenBank data (SRR16526886 for the Nanopore, and SRR16526885 for PacBio HiFi reads, SRR16526887 for the Illumina WGS reads, SRR16588090 and SRR16588091

for the Illumina Hi-C reads, SRR16351760 for the Illumina RNA-seq reads, SRR16351759 for the PacBio Iso-seq reads).

5) Where are the PacBio and ONT reads coming from? It seems that a description of the sequencing is missing. Which library preparation protocol was used? Which sequencing platform?
>>> We have included this information in Supplementary Table S1 and Supplementary File 1. Please see lines 116-117 and Supplementary Table S1, and Supplementary File 1.

6) The assembly is the core of this study thus more details about the actual assembly process are needed. Which parameters were used when running Nextdenovo? The authors might also want to compare their assembly results against an HiFi assembly. Apparently, this assembly was generated, but it is not clear how that was performed. Also, it would be good to compare the results against the results of other assemblers e.g. HiCanu (10.1101/gr.263566.120). Flye (https://doi.org/10.1038/s41587-019-0072-8) could be a good choice due to the high proportion of repetitive elements.
>>>The assembly script (with parameter) with the other results as GigaScience required (
http://gigadb.org/site/guidegenomic) upon submitting the manuscript have been uploaded via FTP for reviewer, we also uploaded this file into figshare (https://doi.org/10.6084/m9.figshare.19165280.v10) and added this information in the revised text (please see lines 134-142). We also included the comparisons of different assemblers (please see lines 293-298 and Supplementary Table S6).

7) What is the definition of properly mapped reads? (line 145) While this is always desired, it is technically challenging to achieve this. Please explain how the mapping/filtering was performed.
>>>We have corrected the wrong expression. It is “properly paired” not “properly mapped”. Please see lines 155-156.

8) Please specify the BLAST default parameters (line 155)
>>>We have included them. Please see lines 167-169.

9) The annotation could also be evaluated by a comparison against the banana genome hub information. How many of the typical Musaceae genes are present? When using BUSCO, it is important to specify the configuration in detail. Depending on these settings, completeness values can change substantially.
>>>The predicted gene sets were compared with the other Musa species, please see Figure S4. We also rephrased the sentences to provide more information, please see lines 208, 372-378. The script to run BUSCO is “busco --in mb_gap_closed_polished.fa --cpu 96 --out Mbe-busco --lineage_dataset /home/w/download/embryophyta_odb10 --mode geno --augustus_species arabidopsis”. It is now provided in the assembly script, and with the other BUSCO result files (“Busco_full_table.tsv”, “Busco_missing_list.tsv” and “Busco_short_summary.txt”) uploaded via FTP for reviewer and also could be found in figshare (https://doi.org/10.6084/m9.figshare.19165280.v10).

10) The identification of gene families based on OrthoFinder is not an ideal approach. Gene families might be broken into multiple orthogroups or merged with non-members. At least for the MYBs, a dedicated annotation workflow is available (https://doi.org/10.1186/s12864-022-08452-5). Please check how the results compare against the OrthoFinder results to evaluate the performance of this analysis.
>>>We agree with the reviewer.
When examining MYB genes identified from MYB_annotator in OrthoFinder results (Supplementary Table S10), it revealed that each type of MYBs were assorted to different orthologous groups except those only one gene occurred. Then some more MYB alike genes identified in OrthoFinder. For example, in M. beccarii the MYB genes were found in 95 orthologous groups, while in these groups there were a total of 398 genes, far larger than 268 MYB genes identified from MYB_annotator. Therefore, more stringent workflow was required for particular gene family analysis but not merely using OrthoFinder.
This is the reason why we used a complementary approach for the transcription

Powered by Editorial Manager® and ProduXion Manager® from Aries Systems Corporation

factors using iTAK. In addition, we ran MYB_annotator. MYB_annotator identified similar amount of the MYB genes compared to iTAK results in M. beccarii and the others (Supplementary Table S10 and S11), and the amount from the former was generally slightly smaller than the latter. It makes us confident in the analyses computed for transcription factors, that complement Orthofinder for which there is easy alternate solution for all other gene families at large scale.

We update the manuscript with MYB results. Please see lines 201-203, 351-356. Supplementary Table S10-S12.

11) Why is all-versus-all BLASTP used if OrthoFinder already constructed groups? If this is for further refinement within orthogroups, it would be important to validate that there are no mis-assignments. Checking the synteny of gene pairs would be important. Apparently, such a synteny analysis was already performed, but the results need to be integrated.

>>> The all-versus-all BLASTP is required in the DupGen_finder pipeline. The pipeline was developed to identify different modes of duplicated gene pairs. The first step of the pipeline is to perform the all-versus-all local BLASTP to search all potential homologous gene pairs within genome. Then, the MCScanX (Wang et al. 2012) algorithm, which is based on MCscan (Wang et al. 2008), was utilized to identify the WGD-derived gene pairs. After excluding WGD-pairs from the whole set of homologous pairs (or BLASTP hits), the pipeline further determines the single-gene duplications, including tandem duplications (TD), proximal duplications (PD), transposed duplications (TRD), and dispersed duplications (DSD). In the text, we have removed the sentences ("…all-versus-all BLASTP …") that are actually not necessary as it is implicit with MCscan. Furthermore, because the same MCscan algorithm was used in the synteny analysis (please see section "Whole genome alignment and synteny analysis" of the text), part of DupGen_finder results (the WGD results) and synteny analysis results were similar. However, considering parameters and algorithms modifications, and different alignment search program used (BLASTP in DupGen_finder pipeline vs. LASTAL in synteny analysis (https://github.com/tanghaibao/jcvi/wiki/MCscan-(Python-version)) in two analyses, some different results might not be fully integrated in the current status.

References:

Tang HB, Wang XY, Bowers JE, Ming R, Alam M, Paterson AH. Unraveling ancient hexaploidy through multiply-aligned angiosperm gene maps. Genome Res. 2008,18(12):1944-1954.

Wang YP, Tang HB, DeBarry JD, Tan X, Li JP, Wang XY, et al. MCScanX: a toolkit for detection and evolutionary analysis of gene synteny and collinearity. Nucleic Acids Res. 2012,40:e49.

12) What is the definition of an enriched GO term? More details should be added about this analysis.

>>>We have included words to make that clear. Please see lines 222-226.

13) REVIGO lacks a reference (https://doi.org/10.1371/journal.pone.0021800).

>>>It actually has been cited in the previous section "Gene family and comparative genomics". Please see lines 226-227.

14) Were the NLR-Annotator results compared against the gene structures produced through the gene prediction process? NLR genes should be one of the best ways to perform the benchmarking of a high quality assembly.

>>>We have included this information. Please see lines 448-454 and Supplementary Table S34.

15) Predictions resulting from KmerGenie and GenomeScope differ by about 20%. Therefore, I would suggest to run some additional tools to narrow down the range of possible genome sizes. Here are some options: findGSE (https://doi.org/10.1093/bioinformatics/btx637), MGSE (https://doi.org/10.1101/607390), Gnodes (https://doi.org/10.1101/2022.05.13.491861), and gce (https://arxiv.org/abs/1308.2012).

>>>Thank you for the suggestion. We have included them and rephrased the sentence in the text accordingly. Except using Illumina short reads in the genome size estimation, we also include HiFi reads for the estimation. Please see lines 124-128,

Powered by Editorial Manager® and ProduXion Manager® from Aries Systems Corporation

288-291 and Supplementary Table S5.

16) It is not clear to me what this sentence means "KmerGenie estimated the optimal k-mer size for the WGS short reads was 87". Is this the best k-mer for an assembly?

>>>Yes, it was automatically provided after performing KmerGenie program. We rephrased the sentence to make it clear. Please see line 287-288.

17) line 309: Was this check only performed based on the M. beccarii assembly or also by screening all reads belonging to that species? It is possible that a technical issue kept the region from being represented in the assembly. Checking the reads would be the more reliable approach.

>>>It was examined by both performing blasting against the assembly and mapping different types of WGS reads against assembly. The Egcen region was absent in M. beccarii. We rephrase the sentence to make it clearer. Please see lines 325-327.

18) MYBs are usually the most abundant group of TFs in plants. How do the numbers and sequences compare to the MYBs in Musa acuminata (https://doi.org/10.1371/journal.pone.0239275; https://doi.org/10.1101/2022.08.15.503939)? This is an available framework to ensure that the TF identification actually works.

>>>We have included this information. Please see answer to point 10, and additional lines 356-359, and Supplementary Table S10-12.

19) The purpose of performing GO and KEGG enrichment analyses is not clear to me. These analyses always produce some results, but what is their relevance? It would be important to look into complex pathways like the flavonoid biosynthesis to see HOW the species are different. Does a particular difference (presence/absence) correlate with the biology/phenotype of the respective species? I find it hard to believe that closely related plant species show a fundamental difference in the ratio of transcriptional control vs. translational control of their gene expression (suggested by the results). It is also unlikely that one species has significantly more membrane proteins than another species. There might be differences with respect to the specific proteins, but an overall differences does not seem plausible. Please check the results and drawn conclusions again.

>>>We have removed redundancy enrichment analyses including content of “helitron repetitive elements”, Supplementary Table S9, S10, S13, S14, S19 and S20. The number of genes in the flavonoid biosynthesis is shown in Supplementary Table S9, we now include the pathways picture (please see Supplementary Figure S15).

However, we did not conduct experiments to compare the relationships of gene numbers and particular flavonoid biosynthesis related traits, nor gene expression experiments to find the relationship, we therefore have not drawn some conclusion like those in the text. Based on current results, some experiments could be conducted in the future. We have rephrased the sentences (removing the word of “over-expression” in sentence, lines 574-576) and included new sentences to make it clear (lines 584-587).

20) Is there a BGC that is specific to M. beccarii and could explain a pathogen response phenotype? BGCs are often associated with evolutionary novel defense components.

>>>We agree with reviewer. According to Table 2, our study did not find M. beccarii specific BGCs, future studies are needed to confirm these BGCs. We added some words to explain that. Please see line 632.

21) The authors investigated gene family expansion with focus on the TFs and looked for mechanisms to explain duplications. I would recommend to screen the assembly for duplicated regions that have been reported in close relatives before e.g. large segmental duplication on chr2 (https://doi.org/10.1534/g3.119.400847). Finding such duplications would support the statement about the TF duplications.

>>>We have included the analyses the reviewer suggested. The results show no large segmental nor recent duplications occurred in M. beccarii (a diploid species). Please see lines 509-516 and Supplementary Figure S13 and S14.

22) Is the large variation in chromosome sizes due to biological differences or could

these be caused by technical artifacts. How does the fraction of missing/unassigned sequences compare between the species? For example, all unassigned sequences in another species could belong to the largest chromosome. The statement about the variation of chromosome sizes would required assemblies of equal quality in all species.

>>> The assembly size of M. beccarii is among the estimations of different programs (we run different genome size estimating as suggested by question 15 from the reviewer, and please see the results in Supplementary Table S5). It currently unknown how different genome sizes among Musaceae species result in different biological functions. We then run structure variation analysis for M. beccarii with M. acuminata version 4, which is the most complete genome in Musaceae, it shows that the increased sequences in M. beccarii come from its different chromosomes. We included new sentences and table for the answers. Please see lines 478-482 and Supplementary Table S35.

23) It seems that the statement about the largest genome size (570Mbp, M. beccarii) is contradicted by some of the following sentences. The authors might want to check this again. It might be necessary to take the ploidy into consideration when comparing genome sizes.

>>>The assembly size of M. beccarii is among the estimations of different programs, please see above question 15. The “contradiction” sentences following the “genome size (assembly ≈ 570 Mbp….” are stating the flow cytometry results, which shows that the genome size estimation using flow cytometry may overestimate the genome size of M. beccarii, and the other Musa species, too. We rephrase the sentence to make it clear. Please see lines 470-471.

Furthermore, according to the Karyological observation of Häkkinen et al. (2007) and reference therein, M. beccarii is a diploid species. Following the method of Busche et al. (2020), by mapping the Illumnia WGS reads against the M. beccarii assembly, the allele frequencies of SNV (single nucleotide variant) obtained showing a single peak frequency of 0.5 (please see Supplementary Figure S14), indicating a diploid species of M. beccarii. The large genome size could be attributed to the repetitive sequences as shown in “Genome size” section of Discussion.

Reference:

Busche M, Pucker B, Viehöver P, Weisshaar B, Stracke R. Genome sequencing of Musa acuminata Dwarf Cavendish reveals a duplication of a large segment of Chromosome 2. G3-Genes Genom. Genet. 2020,10 (1):37-42.

Häkkinen M, Suchuánková P, Doleželová M, Hřibová E, Doležel J. Karyological observation in Musa beccarii var. hottana (Musaceae). Acta Phytotax Geobot. 2007,58(2/3):112-118.

24) The flavonoid biosynthesis is a well studied system of many branching reactions (https://doi.org/10.1104/pp.126.2.485; https://doi.org/10.3390/plants9091103, Fig. 1). Differences in single genes could already explain differences between species. Therefore, it is surprising that large differences picked up by enrichment analyses are reported here. Flavonoids are a large group of > 10k different substances. According to the descriptions in the introduction, M. beccarii might be enriched with anthocyanins (red pigments). ABC transporters are part of the flavonoid biosynthesis (https://doi.org/10.3390%2Fijms140714950; https://doi.org/10.3390/plants11070963) thus a connection between duplications could make sense. Again, a single gene difference could cause a huge phenotypical difference. This does not require a systematic increase of the entire gene family.

>>>We have included new sentences to address these and added the four literature citations the reviewer mentioned. The Supplementary Table S9 summarized the gene numbers of different species in the flavonoid biosynthesis which is obtained by functional annotation using the same procedures. In current study, we sampled a M. beccarii seedling and only performed transcriptome sequencing using its leaves. So we did not perform the comparative transcriptome analysis with different tissues and such analysis among species. We now include gene number comparison of different species in anthocyanin biosynthesis and sentences related to anthocyanin studies. Please see lines 568-571, 580-582, 588-596 and Supplementary Table S9.

Minor comments:

line 80: are polyploid triploids

Powered by Editorial Manager® and ProduXion Manager® from Aries Systems Corporation

	>>> We have removed the "polyploidy". Please see line 81.<br><br>line 305: "After removing sequence length short than 1,000 bp" ... please rephrase.<br>>>>We have rephrased the sentences. Please see lines 323-324.<br><br>line 588: -predator > herbivore?<br>>>>We have replaced "predator" with "herbivore". Please see line 611.<br><br>line 651: is > are<br>>>>We have corrected that. Please see line 674.<br><br>Reviewer: 2<br>Wang et al sequenced and assembled a chromosome-level genome assembly for Musa beccarii, a banana species with high ornamental value. The genome assembly is of high completness and continuity. They described the gene strucutre and function annotation, and repeat annotations. They also compared the genome with relative species from the family Musaceae, including phylogenetic construction, gene family clustering and gene family size evolution, genomic synteny and whole genome duplication, chromosome rearrangement and ancestral genome reconstruction. They also focused on the gene families with specific function, such as NBS-LRR genes and biosyntheic gene clusters. Overall, this is a good paper on genome sequencing, with my recommendation of publication on GigaScience. Before publishing, a few issues need to be addressed.<br><br>1) The English writing must be significantly improved. I was confused with not a few sentences.<br>>>> The manuscript has now been carefully edited by Prof. Pat (J.S.) Heslop-Harrison (one of the authors), a native English speaker. The corrected parts are marked in red.<br><br>2) The Discussion section may be shortened. Some contents that are now placed in Discussion should be moved to Results. For examples, Lines 444-446, Lines 478-481, Lines 494-498,<br>>>>We have removed lines 444-446 and its following sentences, removed paragraphs that include the lines 478-481 and 494-498 to shorten the Discussion section.<br><br>3) Lines 638-639. "Dupgen_finder ..... in the genome." Why you mentioned this at this place? I can not see the meaning of stating some DNA repair genes are DSD.<br>>>>We have removed the sentence.<br><br>4) All figures excluding Fig.1 and Fig.3 are unreadable, due to low resolution.<br>>>>We now provided figures with high resolution. Please click the pictures, download and read them.<br><br>Miscellaneous:<br>Because there are supplementary tables, figures and references removed and new ones added, the order of supplementary tables, figures and references in the revised manuscript are changed. We also update and correct some format errors in some references.<br><br>Please let us know if there are any additional concerns about our manuscript after we have offered these corrections and responses, as we are happy to address any continued issues or proposed changes to the manuscript. Thank you for your time.<br><br>Sincerely yours,<br>Xue-Jun Ge<br>South China Botanical Garden<br>Chinese Academy of Sciences<br>Guangzhou, 510650<br>China
**Additional Information:**	
**Question**	**Response**

Are you submitting this manuscript to a special series or article collection?	No
**Experimental design and statistics**<br><br>Full details of the experimental design and statistical methods used should be given in the Methods section, as detailed in our Minimum Standards Reporting Checklist. Information essential to interpreting the data presented should be made available in the figure legends.<br><br>Have you included all the information requested in your manuscript?	Yes
**Resources**<br><br>A description of all resources used, including antibodies, cell lines, animals and software tools, with enough information to allow them to be uniquely identified, should be included in the Methods section. Authors are strongly encouraged to cite Research Resource Identifiers (RRIDs) for antibodies, model organisms and tools, where possible.<br><br>Have you included the information requested as detailed in our Minimum Standards Reporting Checklist?	Yes
**Availability of data and materials**<br><br>All datasets and code on which the conclusions of the paper rely must be either included in your submission or deposited in publicly available repositories (where available and ethically appropriate), referencing such data using a unique identifier in the references and in the "Availability of Data and Materials" section of your manuscript.<br><br>Have you have met the above requirement as detailed in our Minimum	Yes

Standards Reporting Checklist?	

Powered by Editorial Manager® and ProduXion Manager® from Aries Systems Corporation

Manuscript_clean Click here to access/download;Manuscript;ms-revised-clean.docx

Genome assembly of *Musa beccarii* shows extensive chromosomal rearrangements and genome expansion during evolution of Musaceae genomes

Zheng-Feng Wang[1,2,3], Mathieu Rouard[4], Gaetan Droc[5,6], Pat (J.S.) Heslop-Harrison[1,7,8], Xue-Jun Ge[1,7*]

[1] Guangdong Provincial Key Laboratory of Applied Botany, South China Botanical Garden, Chinese Academy of Sciences, Guangzhou, China

[2] Southern Marine Science and Engineering Guangdong Laboratory (Guangzhou), Guangzhou, China

[3] Key Laboratory of Vegetation Restoration and Management of Degraded Ecosystems, Key Laboratory of Carbon Sequestration in Terrestrial Ecosystem, South China Botanical Garden, Chinese Academy of Sciences, Guangzhou, China

[4] Bioversity International, Parc Scientifique Agropolis II, 34397 Montpellier, France

[5] CIRAD, UMR AGAP Institut, F-34398 Montpellier, France

[6] UMR AGAP Institut, Univ Montpellier, CIRAD, INRAE, Institut Agro, Montpellier, France

[7] Key Laboratory of Plant Resources Conservation and Sustainable Utilization, South China Botanical Garden, Chinese Academy of Sciences, Guangzhou, China.

[8] Department of Genetics and Genome Biology, University of Leicester, Leicester LE1 7RH, UK

*Address for correspondence:
Xue-Jun Ge, E-mail: xjge@scbg.ac.cn

Abstract

Background: *Musa beccarii* (Musaceae) is a species of banana native to Borneo, sometimes grown as an ornamental. Other *Musa* species have a basic chromosome number of x=7, 10 or 11, but *M. beccarii* is the only species with x=9 (2n=2x=18) in the genus, a number shared with the sister genera *Ensete* and *Musella*. *M. beccarii* is in section *Callimusa* of the genus, sister to section *Musa* which includes currently assembled genomes. To better understand genome evolution and diversity in Musaceae, we aimed to generate a high-quality chromosome-scale genome assembly of *M. beccarii*.

Findings: The *M. beccarii* genome was assembled by long read and Hi-C sequencing, with gene annotations using both long Iso-Seq and short RNA-seq reads. *M. beccarii* displayed the largest assembly size, ~570 Mbp, among known Musaceae assemblies, attributed to transposable element expansion and amplification of 45S rDNA sites. We detected extensive genome-wide chromosome fusions and fissions between *M. beccarii* and the other *Musa* and *Ensete* species by synteny analysis, far beyond those expected from chromosome number differences. Within Musaceae, *M. beccarii* showed a reduced number of terpenoid synthase genes which are related to chemical defense and enrichment in lipid metabolism genes linked to the physical defense of the cell wall. Furthermore, type III polyketide synthase (T3PKS) was the most abundant biosynthetic gene cluster in *M. beccarii* with no conservation in the other Musaceae genomes.

Conclusions: *M. beccarii*, as the first chromosome scale genome assembly sequenced in the *Callimusa* section in *Musa*, provides an important resource for understanding evolutionary mechanisms in the Musaceae and builds knowledge of the pangenome.

Keyword: ancestral genome reconstruction, biosynthetic gene cluster, comparative genome, gene family, Musaceae, transcription factors, whole genome duplication

Introduction

Banana is one of the most well-known and highly consumed fruits in the world. In the genus *Musa* (family Musaceae). Molecular phylogenics shows *Musa* is subdivided into two sections, sect. *Musa* and sect. *Callimusa* [1-3]. Unlike the consistent chromosome number in sect. *Musa* (c. 33-50 species)

with x=11 (wild accessions are 2n=2x=22), there are three chromosome numbers in sect. *Callimusa*, x=7, x=9 and x=10 [1,4]. Most of the c. 38 species in sect. *Callimusa* are 2n=2x=20, with lower numbers found in *M. ingens* with 2n=2x=14 and *Musa beccarii* with 2n=2x=18 [5]. Although the chromosome number of *M. beccarii* is unique to the genus, x=9 is shared with species in the two sister genera in the family, *Ensete* and *Musella* [5]. *Musa beccarii* clusters to *M. maclayi* and *M. peekelii*, forming a sub-clade sister to the other sub-clade including *M. gracilis*, while *M. ingens* is the sister of these two sub-clades.

Musa beccarii is endemic in Borneo [5,6]. Its leaves are long and narrow, bright green and pest-free, and the inflorescence, held vertically, has large, bright red bracts (Fig. 1). *Musa beccarii* begins flowering after 6-8 months, and with a height ranging between 1 to 3 m. It is more compact than most other Musaceae, and can be grown as an ornamental indoors [7]. The long lasting bright red and attractive flowers [8] can be used as cut flowers. Currently, *M. beccarii* is formally classified as being of "least concern" as a threatened species [9] although some may consider it endangered due to habitat loss [5, 6] and small, isolated population sizes in the wild. It can be propagated by suckers and tissue culture has been successfully developed in *Musa beccarii* [7, 8], helping its conservation.

Until now, twelve fully assembled and annotated genomes for Musaceae species are available, covering the species *E. glaucum*, *M. acuminata*, *M. balbisiana*, *M. itinerans* and *M. schizocarpa* according to "The Banana Genome Hub" [10]. Only half of them are assembled at chromosome scale. *M. acuminata* was the first species assembled with its 'DH Pahang' genome sequence [11] and the genome was updated in 2021 [12]. The assembly shows that three rounds of ancient whole genome duplications (WGD) occurred in *Musa*. After WGD, many genes related to transcription regulation, signal transduction and translational elongation were retained. Genome comparison indicated genes associated to transcription factors, defense-related proteins, enzymes of cell-wall biosynthesis and enzymes of secondary metabolism are *Musa* lineage-specific. After *M. acuminata*, the *M. balbisiana* genome was assembled [13] and subsequently updated using a double haploid [14]. The majority of edible banana cultivars are triploids resulting from *M. acuminata* and *M. balbisiana* ancestors. Compared to *M. acuminata*, *M. balbisiana* showed more genome fractionation (gene loss) but contained more biotic and abiotic stress resistance properties [14]. *Musa itinerans*

was the third species with its genome assembled [15]. *M. itinerans* is a wild banana, native to south-east Asia and one of the cold- and disease-resistance *Musa* species [15]. *Musa schizocarpa*, the fourth genome assembled [16], is native to Papua New Guinea and a small proportion of its genome is introgressed into many cultivated edible bananas [17]. However, this genome is a draft assembly and no in-depth comparative genomics was conducted. Recently, a draft the genome assembly of *Musa textilis* - an important fiber plant - was published [18] but is not appropriate for accurate comparative genomic analyses as it remains fragmented and incomplete (78.2% complete BUSCO genes).

All previously assembled *Musa* genomes belong to section *Musa* of the genus, while none is reported in sect. *Callimusa*. In the *Ensete* sister group, the first chromosome scale genome assembly of *E. glaucum* (x=9) was recently published by Wang et al. [19] revealing the nature of chromosome rearrangements and fusions between the sister genera. With the shared chromosome number as the genus *Ensete*, it has been speculated that *M. beccarii* could have the most conserved genome structure with regard to the common ancestor between *Musa* and *Ensete* [5], making this assembly a good proxy to study genome evolution in Musaceae and extending the knowledge of the pangenome and structural variants to the *Callimusa* section.

Materials and Methods

Sample collection and sequencing

One *M. beccarii* N.W.Simmonds individual planted in the South China Botanical Garden, Guangdong province, China, was used for genome sequencing. The individual was seedling about 50 cm in height, grown in a green house with no special treatments. We sampled it in 2-3 pm on September 21, 2020.

Genomic DNA was extracted from fresh leaves using the CTAB (cetyl trimethylammonium bromide) method and quality control carried out with a NanoDrop 2000 microspectrophotometer (Thermo Fisher Scientific), Qubit fluorometers (Thermo Fisher Scientific) and gel electrophoresis. High-quality DNA was used for two long- (Nanopore and PacBio HiFi) and one short-read (Illumina) whole genome sequencing (WGS) libraries. To perform Hi-C scaffolding, the genomic DNA

following cross-linkage with formaldehyde was extracted for Hi-C library preparation and Illumina sequencing. Additionally, total RNA from *M. beccarii* leaves of the same individual were extracted and reverse-transcribed to cDNA for PacBio full-length RNA transcripts sequencing (Iso-Seq) and short-read RNA transcripts sequencing library construction: both were used for genome annotation. Further details for these libraries and sequencing modes and platforms are given in Supplementary Table S1 and Supplementary File 1.

Data pre-processing

After sequencing, both short WGS and Hi-C reads were quality trimmed using Sickle v1.33 (Sickle, RRID:SCR_006800) [20] by removing the reads with base quality values less than 30 and length shorter than 80 bp. For short WGS reads, they were further error corrected using RECKONER v1.1 [21]. PacBio HiFi reads were processed using the CCS algorithm v6.0.0 (RRID:SCR_021174, https://github.com/PacificBiosciences/ccs) to obtain consensus reads. Using the error-corrected short WGS reads and/or HiFi reads, the genome size of *M. beccarii* was estimated by KmerGenie v1.7044 [22], GenomeScope 2.0 (RRID:SCR_017014) [23], findGSE [24], GCE v1.0.2 [25], MGSE [26] and Gnodes [27]. Both MGSE and Gnodes were mapping-based genome size estimation, while the others *k*-mer-based. PacBio Iso-seq reads were processed using IsoSeq v3.0 (https://github.com/PacificBiosciences/IsoSeq) to get full-length transcripts. The adapters in Nanopore and PacBio consensus long reads were removed using Porchop v0.2.4 (https://github.com/rrwick/Porechop) and HiFiAdapterFilt v1.0.0 [28], respectively.

Genome assembly

Different assemblers were applied to optimize the initial assembly. For Nanopore reads, they included Nextdenovo v2.3.1 (https://github.com/Nextomics/NextDenovo), flye 2.9.1 (RRID:SCR_017016) [29] and canu 2.2 (RRID:SCR_015880) [30]. For PacBio HiFi reads, they included HiFiasm 0.15.2 (RRID:SCR_021069) [31], flye 2.9.1 and HiCanu (using HiFi mode in canu 2.2) [32]. After considering assembly continuity (see results), Nextdenovo and Hifiasm were finally chosen as suitable assemblers for Nanopore and HiFi reads, respectively. A flowchart showing *M. beccarii* genome assembly steps is depicted in Supplementary Fig. S1 and full scripts

(file named "Mbe_genome_assembly_srcipt.txt") can be found in https://doi.org/10.6084/m9.figshare.19165280.v10. Briefly, the genome was assembled using Nanopore long reads by Nextdenovo. After assembly, the assembly was polished by Racon v1.4.21 (RRID:SCR_017642) [33] and Hapo-G v1.0 [34], and duplications in the assembly were removed by Pseudohaploid (https://github.com/schatzlab/pseudohaploid) and Purge_Dups v1.2.5 (RRID:SCR_021173) [35]. Then, the assembly was corrected by PacBio HiFi reads using Inspector (RRID:SCR_004923, https://github.com/Maggi-Chen/Inspector) and RagTag v2.0.1 (https://github.com/malonge/RagTag). The corrected assembly was scaffolded by Hi-C reads using Scaffhic 1.1 (https://github.com/wtsi-hpag/scaffHiC), Juicer pipeline 1.6 (RRID:SCR_017226) [36] and 3d-dna 201008 (RRID:SCR_017227) [37], and finally gap closed using TGS-GapCloser v1.0.1 (RRID:SCR_017633) [38]. To evaluate the quality of assembly, Benchmarking Universal Single-Copy Orthologs (BUSCO, RRID:SCR_015008) v5.2.2 [39] with the database embryophyta_odb10.2020-09-10 was used. The completeness of the assembly was also evaluated by aligning the Illumina WGS reads using BWA v0.7.17 (RRID:SCR_010910) [40] and looking at the percentage of mapped reads utilizing the samtools v1.9 (RRID:SCR_002105) [41] "flagstat" command.

Repeat annotation

Repeat sequences in the *M. beccarii* assembly were identified by EDTA v1.9.9 (RRID:SCR_022063) [42] and RED v2.0 [43] respectively, and their results were combined using the "merge" command in bedtools v2.29.2 (RRID:SCR_006646) [44]. Based on the combined repeated sequences, *M. beccarii* assembly was masked using the "maskfasta" command in bedtools. For comparison, repeat sequences in *Ensete glaucum*, *M. balbisiana*, *M. itinerans*, *M. schizocarpa*, and *M. acuminata* were also tested by EDTA.

To identify possible two types of centromeric repetitive sequences, Nanica (long interspersed element) [11] and Egcen (tandemly repeated satellite) [19] in *M. beccarii* assembly, blastn 2.12.0+ [45] was used to perform the searching with the default setting including "-strand both -task megablast -evalue 10 -use_index false -dust 20 64 1 -soft_masking true -max_target_seqs 500 -off_diagonal_range 0". Currently, both sequences were found in all Musaceae genomes, but Egcen

is only found in the genus *Ensete* and *Musella* but not in *Musa* [19]. The Nanica sequence was derived from https://banana-genome-hub.southgreen.fr/node/50/353, while Egcen sequence was obtained by asking the authors of Wang et al. [19]. Consensus sequences of the tandemly repeated 5S and 45S rDNA monomers in *M. beccarii* were obtained by assembly of Illumina raw reads to monomers sampled from the Nanopore reads.

Gene predicting and annotation

Structural gene prediction was first performed by LoReAn [46], an automated annotation pipeline designed for eukaryotic genome annotation. Besides *ab initio gene prediction*, proposed by the pipeline, both long and short RNA-seq reads and protein sequences from three species, *M. balbisiana*, *M. schizocarpa*, *M. acuminata* (Supplementary Table S2), were included for RNA-seq and protein evidence-based gene prediction. The obtained results were then used as input into funannotate pipeline v1.8.7 (https://github.com/nextgenusfs/funannotate) to obtain final integrated and consensus gene sets using the command of “funannotate train” and “funannotate predict”, and by applying of “-max_intronlen 100,000 -busco_db embryophyta -organism other” parameters.

After gene prediction, the command “funannotate annotate” was used for gene functional annotation. The annotation databases used included dbCAN v9.0 (RRID:SCR_013208) [47], eggNOG v5.0.2 (RRID:SCR_002456) [48], GO (Gene Ontology, RRID:SCR_002811) [49,50], KEGG (RRID:SCR_012773) [51], InterPro v5.52-86 (RRID:SCR_006695) [52], MEROPS v12.2 (RRID:SCR_007777) [53], Pfam v34.0 (RRID:SCR_004726) [54], and UniProt v2021_03 (RRID:SCR_002380) [55].

Because gene annotation using short- and long-read transcripts identified many isoforms in genes by funannotate pipeline, alternative splicing (AS) events in the genes were investigated with SUPPA v2.3 [56], which classified AS into seven types: skipping exon (SE), alternative 3' (A3) splice sites, alternative 5' (A5) splice sites, mutually exclusive exons (MXE), retained intron (RI), alternative first exons (AFE) and alternative last exons (ALE).

For gene function comparison, the protein-coding genes of all the other species used for our phylogeny analysis (see below) were also functionally annotated as the procedures performed in *M. beccarii*. After annotations, only the longest transcript for each gene in all the species was used for

the following analyses if not mentioned otherwise.

Particularly, considering the importance of transcription factor (TF) genes in the genomes, these genes were identified and compared in Musaceae species using iTAK [57]. In addition, MYB transcription factors, the largest transcription factor families in plants and in Musaceae (see results), were further identified by MYB_ annotator [58].

Gene family and comparative genomics

Gene families among *M. beccarii* and other 14 species (Supplementary Table S3) in monocots were identified using OrthoFinder v2.5.4 (RRID:SCR_017118) [59, 60] by comparing their protein-coding gene sequences. After gene family identification, genes in common, and genes specific to the Musaceae, *Musa* and *M. beccarii* were extracted for predicted gene function comparison. A total of 1,125 single copy ortholog sequences were then selected to perform phylogenomic analysis among the species using RAxML-NG v1.0.3 (RRID:SCR_022066) [61] under the model of JTT+I+G4+F estimated by ModelTest-NG v0.1.7 [62]. Based on the inferred phylogenetic tree, MCMCTree [63] was used to estimate divergence times: nine species pairs were used as calibration points and their estimated divergence time was derived from http://timetree.org/ (Supplementary Table S4). The MCMCTree run used a burn-in of 2,000,000; sample frequency of 10; and sample number of 4,000,000. Two runs were performed to ensure convergence of the posterior distribution. Using the dated tree, CAFE v5 (RRID:SCR_018924) [64] was then applied to identify any gene family (i.e., orthologous group) that had potentially undergone expansion or contraction. When running CAFE, it filtered families that were not at the phylogeny root.

For the above gene sets (family-specific, species-specific and expanded/contracted), an abundance and enrichment analysis according to the GO and KEGG databases was conducted using TBtools v1.098669 [65]. In the enrichment analysis, all the predicted genes with their GO/KEGG annotations were used as the background/reference gene set, the query gene set is the genes particularly obtained from above analyses (for example, expanded/contracted genes). The *P*-values were obtained by hypergeometric tests and corrected using the Banjamini-Hochberg method. For significantly enriched GO terms (the name of the term, i.e. biological process, etc), they were further grouped and visualized with a treemap generated in REVIGO (RRID:SCR_005825) [66].

Whole genome duplication (WGD)

Ancient WGD events in *M. beccarii* and the other five species in Musaceae were detected using wgd v1.2 [67]. To investigate the WGD event positions with respect to speciation events between *M. beccarii* and the other *Musa* species, Ksrates v1.1.1 [68] was used. Ksrates is based on wgd package but rescales the synonymous nucleotide substitution (*Ks*) estimation by considering different *Ks* rates among the lineages in a given phylogeny tree and allow more accurately to infer the speciation events. The phylogenic tree used in ksrate analysis was a simplified phylogeny obtained from gene family analysis above by only considering species in Musaceae and *E. glaucum* was used as an outgroup species in the analysis.

DupGen_finder pipeline [69] was further used to examine how many duplications were derived from WGD or others. By searching homologous gene pairs, besides WGD, DupGen_finder also identified possible gene duplications of tandem duplications (TD), proximal duplications (PD), transposed duplications (TRD), and dispersed duplications (DSD). TD is defined as a one next to one duplication (separated by five or fewer genes), PD is 10 or fewer genes separated duplications, TRD corresponds to transposable element mediated duplications, and DSD are random and non-neighboring duplications. For each duplication gene group, enrichment analysis with GO and KEGG databases was conducted using TBtools v1.098669. For significantly enriched GO terms, they were further grouped and visualized with a treemap generated in REVIGO if needed.

Whole genome alignment and synteny analysis

Syntenic blocks within the *M. beccarii* assembly and between the Musaceae assemblies were analyzed with MCScan (RRID:SCR_017650, Python version) implemented in the jcvi package and visualized both in jcvi (https://github.com/tanghaibao/jcvi) and Shinycircos [70]. The default parameter of synteny analysis in MCScan was used except that the parameter of "minimum number of anchors" was set to 10. MCScanX (RRID:SCR_022067, match score 3, match size 10) [71] was also used and results were imported in SynVisio [72] for syntenic block visualization. D-GENIES v1.2.0 (RRID:SCR_018967) [73] was used to generate and visualize dot plot alignments between Musaceae genome assemblies.

Biosynthetic gene clusters (BGCs)

BGCs in Musaceae species were identified by plantiSMASH v1.0 (Plant Secondary Metabolite Analysis Shell) [74]. To enlarge the cluster searching evidence, the cluster libraries used in PhytoClust [75] were combined in plantiSMASH when running plantiSMASH.

Nucleotide-binding site-leucine-rich repeat (NBS-LRR) gene identification

NBS-LRR genes are the major plant resistance genes serving as an active defense against pathogens [76]. The typical NBS-LRR genes are generally included in three types [77], Toll/interleukin-1 receptor NBS-LRR (TNL), N-terminal coiled-coil motif NBS-LRR (CNL), and resistance to powdery mildew8 NBS-LRR (RNL), in which TNL genes are absent in monocots [77, 78]. Based on InterPro/Pfam annotation results conducted in tested Musaceae species, NBS-LRR genes were identified using the following protein domains: IPR03800, PF00931/IPR002182, PF13855/PF00560/IPR032675, PF05659/IPR008808.

NBS-LRR genes were also detected with NLR-Annotator [79] under default settings. Instead of using annotated proteins that are predicted by gene models and transcriptomic data, NLR-Annotator directly uses genomic sequences to screen possible NLR genes which were confirmed to be most efficient in NLR gene identification. After NLR gene detection, NLR-Annotator categorizes NLR genes as ‘complete’, ‘complete (pseudogene)’, ‘partial’, or ‘partial (pseudogene)’ according to the gene properties.

Ancestral genome reconstruction

Ancestral genomes of Musaceae were reconstructed using AnChro [80], using ginger (*Zingiber officinale*, GenBank accession number of GCA_018446385.1) as outgroup. It relied on SynChro [81] to identify conserved syntenic blocks between different pairs of genomes and then used the blocks in two genomes (with the shortest path connecting them in the phylogenetic tree) to infer the ancestral gene order by comparing them to the reference genomes. During the synteny block inferences, the stringency parameter, that determined the reciprocal best hits within a synteny block, was set to three.

Results

KmerGenie inferred the genome size of *M. beccarii* was 554,284,138 bp under the best selected *k*-mer size of 87 after comparing *k*-mer spectrums of different sizes. Using different programs, the estimated genome size ranged from 547,121,747 bp to 746,096,492 bp (Supplementary Table S5). Two mapping-based programs, MGSE and Gnodes, generated results of between 565,341,681 bp and 661,920,459 bp. The level of heterozygosity in the genome estimated by GenomeScope ranged from 0.287% to 0.815%.

The assembly sizes using different assemblers ranged from 607,623,222 bp (Nextdenovo) to 816,255,026 bp (Canu) with Nonopore reads and 636,694,734 bp (Hifiasm) to 1,247,860,321 bp (HiCanu) with HiFi reads (Supplementary Table S6). The Nexdenovo and Hifiasm assemblers displayed superior contig numbers, average and minimum lengths and N50 values for Nonopore and HiFi reads, respectively, and were used for further genome assembling. Details of Nexdenovo and Hifiasm assembly results were shown in Table 1.

The assembly after Hi-C read scaffolding was 569,617,942 bp with 449 scaffolds and N50 of 67,088,101 bp, and 551,683,906 bp (96.85%) of sequences assembled into 9 chromosomes (Table 1, Figure 2A). The largest chromosome (chr2) is 79,885,826 bp and the shortest chr9 is 38,409,407 bp (Table 1).

BUSCO assessment for the final assembled genome indicated that 98.4% of the completeness score for the embryophyta (1,614 core genes) datasets, including 1,510 (93.6%) complete and single-copy and 78 (4.8%) complete and duplicated genes. Another 11 genes (0.7%) were reported as fragmented, and 15 (0.9%) as missing. The assembly integrity assessed by mapping Illumina WGS reads to the assembled genome using BWA indicated 99.83% mapped reads of which 95.26% were correctly paired.

Repeat annotation

Through the results of EDTA and RED, 51.79% (295,005,341 bp) and 51.45% (293,068,842 bp) of the genome assembly was identified as repetitive regions. According to EDTA, the most abundant repetitive sequences were long terminal repeat (LTR) retrotransposons, accounting for 43.47% (247,628,340 bp) of the assembly, followed by terminal inverted repeats (TIRs) with 5.53%

(31,478,316 bp) of the assembly (Supplementary Table S7). In LTR elements, the largest proportion of sequences were Copia-like (144,383,969bp, 25.35%) and Gypsy-like (51,691,527 bp, 9.07%).

By combining EDTA and RED results, a total of 318,946,703 bp (55.99%) of the assembled genome was annotated and masked as repetitive components. The density of repeat sequences in the assembly was shown in Fig. 2B. Comparative analysis indicated *M. beccarii* contained the highest number and longest length of repetitive sequences (Fig. 3, Supplementary Table S7), mainly LTR retrotransposons and a small number of nonTIR, helitrons.

The 'seed' Nanica repetitive sequences from *M. acuminata* is 5,291 bp long. A blast search reported 822 Nanica like sequences in *M. beccarii* with lengths ranging from 55 to 3,891 bp, and 668 of them had the lengths longer than 1,000 bp. These sequences were found not fully concentrated in the centromere (Supplementary Fig. S2A). The seed sequence of Egcen repeats was 134bp: neither blast to the assembly nor short/long WGS read-mapping on the assembly revealed any similar sequences in *M. beccarii*.

Three sites of the 45S rDNA repeat (18S, 5.8S and 26S rRNA genes and intergenic spacers) were found on chr5 (around bp 21,300,000), chr9 (around bp 38,200,000 near the telomere), and chr7 (around bp 44,400,000). The consensus monomer was 10,402 bp long with a GC content of 60%. The consensus included 17 copies of a tandem repeat (MuTR; GenBank AM905874 to AM905898) although the number of MuTR repeats varied between rDNA monomers in the Nanopore long-molecule reads. Excluding MuTR, present at multiple assembled genome sites, the 45S rDNA repeat was represented in 5.0% of the examined short sequence reads. The 5S rDNA monomer was 432 bp long with a 55.8% GC content, represented in 0.11% of the reads, and major sites were located on chr8 at bp 16,100,000 and chr7 at 37,424,000. Peaks associated with the higher GC content of rDNA sequences (assembly average 38.7% GC) are seen in the GC content plot (Fig. 2B).

Gene prediction and annotation

A total of 39,112 genes coding for 45,461 proteins were predicted in *M. beccarii*. Of these genes, 38,756 (85.25%) were functionally annotated (Supplementary Table S8) with a BUSCO score for completeness equal to 94.8% in embryophyta_odb10.

Among all genes, alternative splicing events were detected in 4,602 genes. Skipping exon events occurred 313 times, alternative 3' splice sites 830 times, alternative 5' splice sites 424 times, mutually exclusive exons 8 times, retained intron 2,847 times, alternative first exons 109 times and alternative last exons 103 times.

A total of 3,168 genes were identified as transcription factors (Figure 2C; Supplementary Table S9) with a similar range in *M. acuminata* and *E. glaucum*. Among transcription factor genes, MYB genes were the most abundant in *M. beccarii* and also in the other Musaceae species (Supplementary Table S10). With the MYB_annotator, a total of 292 MYB genes were identified in *M. acuminata*, similar to 294 previously reported [82], and 268 MYB genes were identified in *M. beccarii* (Supplementary Table S10 and S11). According to the functional annotations, MYB genes associated with "axillary meristem, root growth", "cell wall, lignin, seed oil, axillary meristem", "defense, stress response", "repressor phenylpropanoid, sinapate, lignin" and "stress response, hormone signaling" were more abundant than in other species (Supplementary Table S12). Notably, it included three anthocyanin genes, MB_008808-T1, MB_018229-T1, MB_003891-T1 genes in *M. beccarii* that were found orthologous to the *Musa*MYB-α, -β and –γ in *M. acuminate*, associated to transcriptional activation of anthocyanin biosynthesis in banana [83].

Gene and gene family enrichment

A total of 32,123 orthogroups were identified from a set of 495,640 genes from all species in monocots. For *M. beccarii*, 83.90% (32,815/39,112) of genes were assigned to 50.03% (16,070/32,123) of gene families, and 248 gene families composed of 671 genes were specific to *M. beccarii* (Supplementary Table S13).

In addition, 7,810 gene families were identified to be specific to Musaceae, and 3,531 *M. beccarii* genes were in them. GO and KEGG enrichment analysis indicated these Musaceae specific genes in *M. beccarii* were mainly functionally related to the regulation of protein modification, transcription, and cell wall in GO Biological Process (BP) category (Supplementary Table S14, Supplementary Fig. S3), and flavonoid biosynthesis, tryptophan metabolism and phenylpropanoid biosynthesis in KEGG (Supplementary Table S15).

The five *Musa* species shared 22,000 gene families, of which 11,136 occurred in all five *Musa*

species, and were considered core families in *Musa* (Supplementary Fig. S4). There were 25,754 *M. beccarii* genes in the core families. GO annotation indicated these genes were mainly related to cellular and metabolic processes in BP category, and binding and catalytic activity in Molecular Function (MF) category (Supplementary Fig. S5). Transcription regulator and transporter activities were the two other two representative functions in MF category. Besides shared families, there were 1,062 gene families composed of 1,617 genes were *M. beccarii* specific (Supplementary Fig. S4).

The phylogenetic tree (Figure 2C) showed that *M. beccarii* had an estimated divergence time from the other *Musa* species about 25.26 (95% CI: 8.25-54.84) million years ago. A total of 12,211 gene families were retained for the family expansion and contraction analysis. For *M. beccarii,* it showed that 1,518 families expanded, and 885 families contracted, of which 84 were significantly ($P < 0.05$) expanded and 50 were significantly contracted. Enrichment analysis indicated that significantly expanded gene families were mainly functionally related to transcription, carbohydrate metabolism, and membrane transport (Supplementary Table S16 and S17, Supplementary Fig. S6). While significantly contracted gene families, were mainly functionally related to defense response in GO annotation, (mono)terpenoid biosynthesis and translation factors in the KEGG annotation (Supplementary Table S18 and S19).

In addition, enrichment analysis for genes with alternative splicing indicated they were mainly related to mRNA 3'-end processing, amino acid catabolic processes, phosphorus metabolic processes, response to stress (such as DNA repair), and taurine and hypotaurine metabolism (Supplementary Table S20 and S21, Supplementary Fig. S7)

Gene duplicates

All Musaceae species underwent the same three ancient WGD events (Fig. 4A) and based on these events, we infer that the split of the five *Musa* species occurred after the WGD events (Fig. 4B).

Gene duplications revealed 11,244 gene pairs possibly derived from whole genome duplication, 531 pairs derived from tandem duplications, 646 pairs derived from proximal duplications, 2,313 pairs derived from transposed duplications, and 7,690 pairs derived from dispersed duplications in *M. beccarii*. Enrichment analysis indicated that duplicated genes due to whole genome duplications were mainly related to transcription, signaling, defense, environment adaptation, and root

development (Supplementary Table S22 and S23, Supplementary Fig. S8 and S9A). The genes due to tandem duplications were enriched to various metabolic processes related to stress response (such as glutathione, and phenylpropanoid metabolism) and defense (such as cell wall formation and membrane transport) (Supplementary Table S24 and S25, Supplementary Fig. S10 and S9B). The genes associated with proximal duplications were mainly related to benzoxazinoid, terpenoid, and flavonoid biosynthesis, membrane transport and cell wall formation (Supplementary Table S26 and S27, Supplementary Fig. S9C). The genes resulting from transposed duplications were mainly related to ion transport (Supplementary Table S28). The genes deriving from dispersed duplications were mainly related to DNA repair, monosaccharide metabolic process, and prokaryotic defense system (Supplementary Table S29 and S30, Supplementary Fig. S11 and S9D).

Whole genome alignment and synteny analysis

Overall, *M. beccarii* exhibited high syntenic relationships with the other Musaceae assemblies, showing tens of major syntenic blocks of genes with extensive rearrangements including fusions, fissions and translocations (Fig. 4C and Fig. 5). Chr4 of *M. beccarii* showed the highest conserved relationship with chr4 of the other *Musa* species (Fig. 5). Chr5, which was the only one conserved between *E. glaucum* and *M. acuminate*, was divided to chr3 and chr5 in *M. beccarii* (Supplementary Fig. S12), suggesting a fission specific to *M. beccarii*. For the other chromosomes, *M. beccarii* were highly rearranged with for instance chr3 syntenic with 5 chromosomes of both *E. glaucum* and *M. acuminata* (Supplementary Fig. S12).

Analysis of the ancient whole genome duplications within *M. beccarii* by synteny analysis resulted in 233 syntenic blocks containing 9,594 genes and 5,512 gene pairs. The longest syntenic block size was 8,645,195 bp containing 44 gene pairs between chr5 and chr6, and the smallest was 161,642 bp containing 15 gene pairs between chr2 and chr3 (Supplementary Table S31). The syntenic relationship is illustrated in the CIRCOS plot (Fig. 2B).

M. beccarii had 196, 111, 155, and 141 syntenic blocks with *Ensete glaucum*, *M. balbisiana*, *M. acuminata*, and *M. schizocarpa* respectively. Among these blocks, all the largest blocks occurred in the chr4s of *M. beccarii* and the other *Musa* species (Fig. 4C and Fig. 5). These largest blocks contained 1,776, 2,495, 1,675 gene pairs for *M. beccarii* with *M. balbisiana*, *M. acuminata*, and *M.*

schizocarpa respectively, including a total of 2,602 genes in *M. beccarii* chr4.

Biosynthetic gene clusters (BGCs)

PlantiSMASH identified 66 possible BGCs in *M. beccarii* (Table 2 and S32), which was second most abundant in tested Musaceae species and lower than *M. acuminata* which was with 72 clusters. The most abundant BGCs in all Musaceae species are alike to type III polyketide synthase (T3PKS) and tomatine clusters. The BGCs in chr4 of *M. beccarii* and their syntenic genes in the chr4s in the other *Musa* species are shown in Figure 4D, indicating BGCs are not conserved in *Musa*. The BGC *per se* and the genes in them showed substantial gains and losses.

NBS-LRR gene identification

Using annotated protein sequences, the highest number of CNL genes was found in *M. itinerans* (59 genes), and the lowest was found in *M. balbisiana* (14) in Musaceae species. *M. beccarii* displayed 31 CNL genes, which was the third-highest among tested Musaceae species (Fig. 2C). All Musaceae species had only one RNL in each.

NLR-annotator identified the most complete and highest overall number of NBS-LRR genes in *M. beccarii* (Supplementary Fig. S2B, Supplementary Table S33), with the highest abundance in chr6, and absence in chr5 (Supplementary Fig. S2B). However, when identifying the NBS-LRR genes using predicted genes with NLR-annotator, only 74 could be found in *M. beccarii* (Supplementary Table S34), much lower than the 179 genes derived using assembled genome to identification. In the other Musaceae species, *M. balbisiana* also showed low rate of NBS-LRR genes using predicted genes compared to that using genome (43/96). In *M. acuminata*, the most compete assembled genome, the rate was 111/128. In *M. itinerans,* the rate was 149/138, which most likely caused by gene prediction errors and a fragmented draft assembly.

Ancestral genome reconstruction

Ancestor reconstruction revealed 86 contigs for the last common ancestor (LCA) genome and between 19 and 40 contigs for the intermediate ancestors in Musaceae (Fig. 6). Although these ancestral genomes are fragmented, they display the complex chromosomal rearrangements that

occurred between *Ensete*, *Musa* sect. *Musa* and *Musa* sect. *Callimusa*. Considering macro- and micro-rearrangements in these Musaceae species, number of contig rearrangements ranged from 18 for *M. acuminata* to 91 for *M. beccarii*, consistent with the phylogeny.

Discussion

Genome size

The chromosome scale assembly of *Musa beccarii* identified nine pseudomolecules between 38 and 79 Mbp long (Fig. 2, Table 1), more variable than those in other *Musa* species (eg *M. acuminata*, 35 to 51Mbp) [12]. *M. beccarii* has the largest genome size (assembly ≈ 570 Mbp; consistent with various estimates from *k*-mers) in Musaceae [5, 84]. Using DNA flow cytometry, the genome size of *M. beccarii* was estimated between 764 Mb and 804 Mb [5, 85], which might be overestimated compared to the *k*-mer/mapping based genome size estimation. Like other *Musa* species, the genome size estimated by flow cytometry [84-86] was larger than assembled genomes (534-578 vs. ~457 Mbp in *M. balbisiana*, 591-646 vs. ~469 Mbp in *M. acuminata*, 704 vs. 515 Mbp in *M. schizocarpa*). The different genome sizes between different methods were influenced by many factors, including the accuracy of flow cytometry and the reference values used based on chemical measurements [87-89], different samples [86, 90], reference genome staining, and incorrect genome assembly. However, no matter which methods are used to estimate the genome size, the data indicate *M. beccarii* had the largest measured genome size in *Musa* [5, 84]. The relationship between genome sizes of Musaceae species and their biological functions need further investigation. Furthermore, by comparing the structure variations between *M. beccarii* and *M. acuminate*, increased genome sizes in *M. beccarii* arises in different chromosomes (Supplementary Table S35), indicating the increase is not a burst of a single chromosome.

Repetitive sequences, especially transposable elements (TEs), are important elements driving genome expansion [91-93], at least in species with genomes smaller than 5 Gbp [94]. We detected a clear increase of TE number and length in *M. beccarii*, which is about 30 Mbp-106 Mbp larger than *E. glaucum* and the other three *Musa* species (Supplementary Table S7; Fig. 3). Previous studies using low coverage sequencing observed that *M. beccarii* contained the highest repetitive sequence among five tested *Musa* species, including *M. balbisiana* and *M. acuminata* [84], and suggesting

repetitive sequence increase might be one important activity causing the larger genome size of *M. beccarii*. Nevertheless, for each TE, only LTR-unknown and helitron in *M. beccarii* showed consistently higher totals, 51,552,844 bp and 15,898,685 bp respectively, among our studied species (Supplementary Table S7). By using LTR markers, Häkkinen et al. [6] identified rich and distinct LTR in *M. beccarii*, suggesting diversification of LTR resulting in unknown LTRs in *M. beccarii*.

As well as the differences in retrotransposons, the assembly also showed the presence of three pairs of loci of 45S rDNA, compared to only one in the other *Musa* and *Ensete* species assemblies (consistent with the in situ hybridization results of Bartoš et al.) [85]. The 45S rDNA (on three chromosomes) represented 5.0% of the Illumina sequence reads in *M. beccarii*, compared to 1.2% in *Ensete glaucum* (at one chromosome) [19], so increased rDNA copy number is responsible for some of the increase in assembled genome size.

Gene family evolution

Gene family expansion due to duplications in *Musa* including *M. beccarii* was mainly caused by ancient WGD events. Globally, transcriptions factors (TFs) are similarly abundant among Musaceae species and higher than all the other monocots used in our sampling except *Zingiber officinale* (Fig. 2C, Supplementary Table S9), which is a tetraploid species. Enrichment analysis in *M. beccarii* indicated that TFs are one of the main genes retained after WGD events (Supplementary Table S23; Supplementary Fig. S8 and S9A). Because Musaceae species underwent the same WGD events, this result also reflects the whole adaptation by TFs in Musaceae, which are consistent with previous results revealed in *M. acuminata* and *M. itinerans* assembled genomes [11, 15]. To find possible duplications caused by polyploidies or aneuploidies, the mapping results of Illumina WGS reads against *M. beccarii* assembly were examined including the mapping coverage distribution along the chromosomes and allele frequencies of SNV (single nucleotide variant) according to Busche et al. [95]. It showed that the mapping coverages displayed constant along all the chromosomes (Supplementary Figure S13), and one peak allele frequency of 0.5 displayed in all the chromosomes (Supplementary Figure S14), indicating no large segmental duplications occurred in the diploid *M. beccarii*.

Nevertheless, we observed the contraction of gene families related to defense response,

monoterpenoid biosynthesis, and terpenoid backbone biosynthesis (Fig. 2C, with *M. beccarii* having fewer genes than other Musaceae; Supplementary Table S18 and S19). Terpenoids are important natural products [96-98]. They encompass a diverse components and have various applications, particularly for defense [97], acting as toxic compounds against biological stress agents in plants. Except for terpenoid backbone and monoterpenoid biosynthesis, according to KEGG, there are other terpenoid biosynthesis (TB)-related pathways, such as steroid biosynthesis (ko00100), ubiquinone, and other terpenoid-quinone biosynthesis (ko00130), limonene and pinene degradation (ko00903), diterpenoid biosynthesis (ko00904), brassinosteroid biosynthesis (ko00905), carotenoid biosynthesis (ko00906), zeatin biosynthesis (ko00908), sesquiterpenoid and triterpenoid biosynthesis (ko00909). Gene families did not contract in these pathways. Because typical terpenoid synthase genes are characterized by two conserved domains with Pfam ID PF01397 and PF03936 [98], comparing the genes bearing these domains in Musaceae, reveals that the primary terpenoid synthase genes in *M. beccarii* are minimum, but not largely decreased (Supplementary Table S9, Fig. 2C). The significantly expanded gene families in *M. beccarii*, which are mostly related to transcription, carbohydrate metabolism, and membrane transport (Supplementary Table S16, S17, Supplementary Fig. S6), are involved in a wide range of functions for plant growth, development, and defenses [99-102]. Therefore, the expanded genes may help balance the growth, development, and defense in *M. beccarii*. From the defense aspect, we also examined NBS-LRR genes in *M. beccarii* and the others in the phylogeny (Fig. 2C). These genes in *M. beccarii* were more abundant (179 genes by the sensitive NLR-annotator analysis [79] in the assembly), than in other Musaceae (67 to 138 genes), although much lower than most of the species out of Musaceae (Fig 2C, Supplementary Table S9). Therefore, combing Pfam annotation suggested that NBS-LRR genes might not be a priority in disease defense in Musaceae.

Cell wall as defense (lipid metabolism and ABC transporters)

Apart from those directly for defense, genes such as cutin, suberin, and wax (CSW) play a critical role in "physical defense" in plants [103-105]. They are lipids [103, 106] and are formed by fatty acids and glycerol. They consist of the extracellular hydrophobic layer of cell walls in the plant, provide mechanical support and protect the plant from desiccation, extreme temperature, UV, and

pathogen/pest attack [103, 105]. According to KEGG PATHWAY Database (https://www.genome.jp/kegg/pathway.html), the lipid metabolism contains 16 pathways, of which 14 of them are used in our studied species (Supplementary Table S9). Comparative analysis indicates the Musaceae species do not contain more genes in these pathways than the other species, and only a marginal increase in gene numbers occurs in fatty acid elongation (ko00062), cutin, suberine and wax biosynthesis (ko00073), glycerophospholipid metabolism (ko00564) and ether lipid metabolism (ko00565) pathways for *M. beccarii* among Musaceae species (Fig. 2C).

Because cell wall lipids are synthesized in epidermal cells, they need to be exported to the plant surface. ATP-binding cassette (ABC) transporters are essentially required [103, 107]. Among these transporters, the G family is proposed responsible for these lipids secretion [108]. We identified high gene numbers in the ABC transporters G families in all Musaceae species (Supplementary Table S36, both InterPro and eggNOG annotations). For the ABC transporters overall, Musaceae species showed the highest gene numbers with eggNOG annotation but not with other annotation pipelines, and *M. beccarii* had the highest number with InterPro annotation. ABC transporters are one of the largest protein families in nature [109]. They bind, hydrolyze adenosine triphosphate (ATP) and mediate cellular transport processes [109, 110]. The molecules they transport include ions, amino acids, sugars, lipids, peptides, proteins, antibiotics, and so on. ABC transporters expansion has been demonstrated to go along with increasing abiotic and biotic stress defenses in plants, then functionally driven adaptation [109, 111, 112].

Flavonoid biosynthesis as defense

Flavonoids are a ubiquitous group of polyphenolic compounds in plants. As important secondary metabolites, they have been studied extensively from the synthesis to the biological activities [113-116], including in Musaceae species [117, 118], but not reported by comparative genomics in Musaceae. We noticed significant gene enrichment in flavonoid biosynthesis in *M. beccarii* (Supplementary Table S15). When considering the genes involved in this biosynthesis, it is revealed that Musaceae species displayed a high abundance among all compared species, in which *M. beccarii* showed the second largest (Supplementary Table S9, Fig. 2C). The high overall gene numbers in Musaceae species are majorly derived from the naringenin 7-O-methyltransferase

(NOMT, with KEGG Orthology term of K22440) genes in them. NOMT gene can catalyze the methylation of naringenin to produce sakuranetin (Supplementary Fig. S15), a phytoalexin with strong anti-fungal activity [119]. Therefore, the accumulation of NOMT genes in flavonoid biosynthesis indicates flavonoid is functionally important in the disease defenses in Musaceae as well as being a potentially valuable component of the harvested *Musa* crop [120]. It has been reported that ABC transporters are one of the important media during flavonoid transport in the plants [121,122]. Interestingly, both flavonoid biosynthesis and ABC transporters-related genes were enriched in tandem and proximal duplications in *M. beccarii* (Supplementary Table S25 and S27), and these two duplicates are found to evolve strongly for self-defense in plants [69]. In the future, comparison of the presence/absence and expressions of flavonoid biosynthesis related genes in Musaceae and the other species with their biology/phenotype will help increase our understanding both the defense and other functional activities in Musaceae.

M. beccarii has bright red flowers (Fig. 1). Anthocyanins are important substances connect to the flower colors [113, 123], and their biosynthesis pathway is associated with flavonoid biosynthesis. It has also been reported that anthocyanins were involved in the red peel of *Musa AAA Cavendish* cv. Baxi [124] and purple peel of *Musa itinerans* [125] fruits. We compared gene number in the anthocyanins biosynthesis and found Musaceae species did not display a large number variations compared to the other species (Supplementary Table S9). Given that flavonoid/anthocyanin biosynthesis is mostly regulated at the transcriptional level [117; 126; 127], future comparative transcriptome analysis among different tissues and species are required to reveal the formation of red flower color in *M. beccarii*.

Biosynthetic gene clusters (BGCs)

We identified diversified BGCs in Musaceae. BGCs are not randomly ordered genes along chromosomes, which may optimize the synthesis pathways of natural products in living organisms [128, 129]. Among the Musaceae BGCs, one most developed cluster is alike to T3PKSs (Table 2, Supplementary Table S32). T3PKSs are homodimer ketosynthases widely distributed in plants, fungi, and bacteria [130]. They take part in various important biosynthesis of secondary metabolites related to polyketides, produce a broad class of natural products [131, 132], function as defense

response, and development [132-134]. In *Musa*, it has been reported that T3PKS can initiate the phenylphenalenones biosynthesis, while phenylphenalenones are the major phytoalexins against multiple pathogens in *Musa* [134]. Thereafter, the T3PKS BGCs in *Musa* are valuable for further investigation to improve their defense systems.

Tomatine alike clusters are the other highest BGCs identified in *Musa* species. Tomatine is a steroidal glycoalkaloid saponin found in tomatoes and some other *Solanum* species [135,136]. It has anti-pathogen and -herbivore properties serving as natural defenses in the plant [136-138]. In tomatine biosynthesis, the primary genes are glycosyltransferases (GT) [139, 140]. It performs glycosylation of tomatidine, a steroidal alkaloid (SA) and phytotoxic, for the formation of tomatine, and reduces the toxicity of SA metabolites to the plant cell [140-142]. In *Musa*, although a tomatine alike steroidal saponin, which showed highly effective resistance to black Sigatoka, was reported in *Musa acuminata* for years [142], its BGC is still poorly resolved [143]. Therefore, the BGCs observed in the current study will provide valuable references for investigating its biosynthesis in *Musa*.

GTs are ubiquitous enzymes involved in various plant secondary metabolisms [144]. They generally function to glycosylate substrates with sugar moieties attached to the aglycones and then form glycosidic bonds. Their acceptor substrates can be sugars, lipid, protein, nucleic acid, antibiotic, or small molecules [145, 146]. Glycosylation is highly diverse by using various sugar moieties, leading to a wide range of biological functions, important to plant growth, development, and defense responses [147]. In *M. beccarii*, we detected highly abundant GT-related genes (Supplementary Table S37, Fig. 2C), which may also be attributable to its environmental adaptation. Furthermore, among these GTs, the GT family 61 (GT61) is tightly related to the xylan biosynthesis of the cell wall [148, 149]. Xylans are hemicelluloses that can influence cell wall recalcitrance and play a crucial role against herbivores and pathogens [150]. In *M. beccarii*, we also detected the highest number of GT61 genes in Musaceae (Supplementary Table S37, Fig. 2C). DupGen_finder indicated that 32 (66.7%) of these genes derived from ancient WGD, which agrees with the previous study that demonstrated the major duplication in GT families was generated from WGD [149, 151]. Experimental studies are needed to characterize more fully potential BGCs.

Chromosome rearrangement

We observed substantial numbers of chromosomal reorganization events involving chromosomal fusion and fission in *M. beccarii* and the other three *Musa* species (Figures 4C and 6), with only one chromosome remaining largely intact. The extensive fusion/fission events between *M. beccarii* in *Musa* section *Callimusa* and the other *Musa* section *Musa* species, are similar in number to those between *Musa* and *Ensete* (Figures 4C, 5 and 6 and S12), and is not only a consequence of the reduced chromosome number (x=9 vs. x=11). This result strongly supports the division of *M. beccarii*, and the other studied *Musa* species in two different sections. Chromosomal fusion and fission are important mechanisms of speciation [152-154]. However, current ancestral reconstructions did not allow us to infer ancestral chromosome numbers between *Musa* sections or between *Ensete*.

The abundance and expansion of transposable elements, as shown above, may contribute to enabling the evolutionary genome rearrangements in *M. beccarii*, leading to large structural differences from the other studied *Musa* species. Structural rearrangements mediated by various families of TE elements have been reported in other plants [155,156]; chromosome-scale assemblies anchored by long-molecule sequencing will enable further study of association of TEs including retroelements to chromosomal rearrangement.

Our results confirmed that Egcen centromeric tandemly repeated sequence of *Ensete* are absent in *Musa* [19]. Since these repeats are also detected in *Musella*, the third genus in Musaceae, one possible reason for the absence of Egcen repeats in *Musa* could be attributed to ancestral centromeres breakage causing segments loss in *Musa* because centromeres are hotspots of chromosome rearrangements [157, 158].

Alternative splicing related to DNA repair systems

In particular, we observed alternative splicing (AS) happening in at least 11.7% of genes. However, this is underestimated because we did not examine the transcriptomes of different tissues and their different developmental stages. Enrichment analysis in these AS occurring genes revealed they were related to important cellular responses, and DNA repair systems, including such as DNA repair, nucleotide excision repair, and replication and repair (Supplementary Table S20 and S21). A wide

variety of stress conditions can induce DNA damage. DNA repair systems are, therefore, important to keep chromosome stability in eukaryotic cells [159-161]. AS is a post-transcriptional mechanism that produces many functional proteins from a limited number of genes. AS in *M. beccarii* then shows reinforcement in the DNA repaired pathway and the other processes for evolutionary adaptation.

Conclusion

The assembly of a genome from section *Callimusa* of the *Musa* genus is important to enable us to develop a pangenome model of Musaceae. The new data shows the extensive rearrangements and expansions of the genome that have occurred, including new insight into the range of structural chromosome variation present in the Musaceae. Both the details of the genes and transcription factors, and the structural analysis of the genome, are important to identify and conserve the biodiversity present in the genus, and in making novel variation available to use for plant breeding and meeting the challenges in banana crops and more widely.

Data availability

We deposited the sequenced reads to NCBI Sequence Read Archive under the accession number SRR16526886 for the Nanopore, and SRR16526885 for PacBio HiFi reads, SRR16526887 for the Illumina WGS reads, SRR16588090 and SRR16588091 for the Illumina Hi-C reads, SRR16351760 for the Illumina RNA-seq reads, SRR16351759 for the PacBio Iso-seq reads. The high-quality, assembled genome was submitted to GenBank under the accession number JAIWVJ000000000. Genome Assembly, gene annotation data, and transcriptomic data are also available on the Banana Genome Hub (http://banana-genome-hub.southgreen.fr/) for download or exploration via a dedicated Genome Browser (Jbrowse) and syntenic browser (SynVisio).

Ethics Approval and Consent to Participate

No ethical approval/permission is required to obtain the materials and preform the research in this study. Plant material was collected before 1993 and was received at the South China Botanical Garden under an agreement from an European botanical garden.

Competing Interests

The authors declare that they have no competing interests.

Fundings

This work was financially supported by the National Natural Science Foundation of China (No. 32070237, 31261140366), and the Strategic Priority Research Program of Chinese Academy of Sciences (Grant No. XDB31000000).

Authors' contributions

XJG and ZFW designed this experiment. ZFW, MR, GD and PHH conducted genetic work and data analyses. ZFW, XJG, MR and PHH drafted the manuscript. ZFW, MR, GD, PHH, and XJG revised the manuscript. All authors gave final approval of the paper.

References

1. Li L-F, Häkkinen M, Yuan Y-M, Hao G, Ge X-J. Molecular phylogeny and systematics of the banana family (Musaceae) inferred from multiple nuclear and chloroplast DNA fragments, with a special reference to the genus *Musa*. *Mol Phylogenet Evol*. 2010;57(1):1-10.
2. Häkkinen M. Reappraisal of sectional taxonomy in *Musa* (Musaceae). *Taxon*. 2013,62: 809-813.
3. Fu N, Ji M, Rouard M, Yan HF, Ge XJ. Comparative plastome analysis of Musaceae and new insights into phylogenetic relationships. *BMC Genom*. 2022,23(1):223
4. Christelová P, Valárik M, Hřibová E, De Langhe E, Doležel J. A multi gene sequence-based phylogeny of the Musaceae (banana) family. *BMC Evol Biol*. 2011,11:103.

5. Häkkinen M, Suchuánková P, Doleželová M, Hřibová E, Doležel J. Karyological observation in *Musa beccarii* var. *hottana* (Musaceae). *Acta Phytotax Geobot.* 2007,58(2/3):112-118.
6. Häkkinen M, Teo CH, Othman YR. Genome constitution for *Musa beccarii* (Musaceae) varieties. *Acta Phytotaxon Sin.* 2007,45(1):69-74.
7. Natarajan N, Sundararajan S, Ramalingam S, Chellakan, PS. Efficient and rapid in-vitro plantlet regeneration via somatic embryogenesis in ornamental bananas (*Musa* spp.). *Biologia*. 2020,75:317-326.
8. Rashid K, Nezhadahmadi A, Othman RY, Ismail NA, Azhar S, Efzueni S. Micropropagation of ornamental plant *Musa beccarii* through tissue culture technique using suckers and male buds as explants. *Life Sci J*. 2012,9(4):2046-2053.
9. Allen R. *Musa beccarii*. The IUCN Red List of Threatened Species 2019: e.T121033043A121033225. 2019. https://dx.doi.org/10.2305/IUCN.UK.2019-3.RLTS.T121033043A121033225.en. Accessed 13 July 2022.
10. Droc G, Martin G, Guignon V, Summo M, Sempéré G, Durant E, et al. The banana genome hub: a community database for genomics in the Musaceae. *Hortic Res.* 2022:uhac221.
11. D'Hont A, Denoeud F, Aury JM, Baurens FC, Carreel F, Garsmeur O, et al. The banana (*Musa acuminata*) genome and the evolution of monocotyledonous plants. *Nature.* 2012,488(7410): 213-217.
12. Belser C, Baurens FC, Noel B, Martin G, Cruaud C, Istace B, et al. Telomere-to-telomere gapless chromosomes of banana using nanopore sequencing. *Commun Biol.* 2021,4(1):1047.
13. Davey MW, Gudimella R, Harikrishna JA, Sin LW, Khalid N, Keulemans J. (2013). "A draft *Musa balbisiana* genome sequence for molecular genetics in polyploid, inter- and intra-specific *Musa* hybrids". *BMC Genomics*. 2013,14:683.
14. Wang Z, Miao H, Liu J, Xu B, Yao X, Xu C, et al. *Musa balbisiana* genome reveals subgenome evolution and functional divergence. *Nat Plants.* 2019,5:810-821.
15. Wu W, Yang YL, He WM, Rouard M, Li WM, Xu M, et al. Whole genome sequencing of a banana wild relative *Musa itinerans* provides insights into lineage-specific diversification of the *Musa* genus. *Sci Rep*. 2016,6:31586.
16. Belser C, Istace B, Denis E, Dubarry M, Baurens FC, Falentin C, et al. Chromosome-scale

assemblies of plant genomes using nanopore long reads and optical maps. *Nat Plants.* 2018,4(11):879-887.

17. Eyland D, Breton C, Sardos J, Kallow S, Panis B, Swennen R, et al. Filling the gaps in gene banks: Collecting, characterizing, and phenotyping wild banana relatives of Papua New Guinea. *Crop Sci.* 2021,61:137-149.
18. Galvez LC, Koh RBL, Barbosa CFC, Asunto JC, Catalla JL, Atienza RG, et al. Sequencing and de novo assembly of Abaca (*Musa textilis* Nee) var. Abuab Genome. *Genes*. 2021,12(8): 1202.
19. Wang Z, Rouard M, Biswas MK, Droc G, Cui D, Roux N, et al. A chromosome-level reference genome of *Ensete glaucum* gives insight into diversity and chromosomal and repetitive sequence evolution in the Musaceae. *Gigascience*. 2022,11:giac027.
20. Joshi NA, Fass JN. Sickle: A sliding-window, adaptive, quality-based trimming tool for FastQ files (Version 1.33). 2011. https://github.com/najoshi/sickle. Accessed 3 September 2021.
21. Długosz M, Deorowicz S. RECKONER: read error corrector based on KMC. *Bioinformatics*. 2017,33:1086-1089.
22. Chikhi R, Medvedev P. Informed and automated *k*-mer size selection for genome assembly. *Bioinformatics*. 2014,30:31-37.
23. Vurture GW, Sedlazeck FJ, Nattestad M, Underwood CJ, Fang H, Gurtowski J et al. GenomeScope: fast reference-free genome profiling from short reads. *Bioinformatics*. 2017,33: 2202-2204.
24. Sun H, Ding J, Piednoël M, Schneeberger K. findGSE: estimating genome size variation within human and Arabidopsis using k-mer frequencies. *Bioinformatics*. 2018,34:550-557.
25. Liu B, Shi Y, Yuan J, Hu X, Zhang H, Li N, et al. Estimation of genomic characteristics by analyzing k-mer frequency in de novo genome project. arXiv. 2013:1308.2012. https://arxiv.org/abs/1308.2012.
26. Pucker B. Mapping-based genome size estimation. BioRxiv. 2019. https://doi.org/10.1101/607390.
27. Gilbert DG. Genes ruler for genomes, Gnodes, measures assembly accuracy in animals and plants. BioRxiv. 2022. https://doi.org/10.1101/2022.05.13.491861.

28. Sim SB, Corpuz RL, Simmonds TJ, Geib SM. HiFiAdapterFilt, a memory efficient read processing pipeline, prevents occurrence of adapter sequence in PacBio HiFi reads and their negative impacts on genome assembly. *BMC Genom*. 2022,23(1):157.

29. Kolmogorov M, Yuan J, Lin Y, Pevzner PA. Assembly of long, error-prone reads using repeat graphs. *Nat. Biotechnol*. 2019,37:540-546.

30. Koren S, Walenz BP, Berlin K, Miller JR, Phillippy AM. Canu: scalable and accurate long-read assembly via adaptive k-mer weighting and repeat separation. *Genome Res*. 2017,27(5):722-736.

31. Cheng H, Concepcion GT, Feng X, Zhang H, Li Heng. Haplotype-resolved de novo assembly using phased assembly graphs with hifiasm. *Nat. Methods*. 2021,18(2):170-175.

32. Nurk S, Walenz BP, Rhiea A, Vollger MR, Logsdon GA, Grothe R, et al. HiCanu: accurate assembly of segmental duplications, satellites, and allelic variants from high-fidelity long reads. *Genome Res*. 2020,30(9):1291-1305.

33. Vaser R, Sović I, Nagarajan N, Šikić M. Fast and accurate de novo genome assembly from long uncorrected reads. *Genome Res*. 2017,27(5):737-746.

34. Aury JM, Istace B. Hapo-G, haplotype-aware polishing of genome assemblies with accurate reads. *NAR Genom Bioinform*. 2021,3(2):lqab034.

35. Guan, DF, McCarthy SA, Wood J, Howe K, Wang YD. Identifying and removing haplotypic duplication in primary genome assemblies. *Bioinformatics*. 2020,36:2896-2898.

36. Durand NC, Shamim MS, Machol I, Rao SSP, Huntley MH, Lander ES, et al. Juicer provides a one-click system for analyzing loop-resolution Hi-C experiments. *Cell Syst*. 2016,3(1):95-98.

37. Dudchenko O, Batra SS, Omer AD, Nyquist SK, Hoeger M, Durand NC, et al. De novo assembly of the Aedes aegypti genome using Hi-C yields chromosome-length scaffolds. *Science*. 2017,356(6333):92-95.

38. Xu M, Guo L, Gu S, Wang O, Zhang R, Peters BA, et al. TGS-GapCloser: A fast and accurate gap closer for large genomes with low coverage of error-prone long reads. *Gigascience*. 2020,9(9):giaa094.

39. Seppey M, Manni M, Zdobnov EM. BUSCO: Assessing genome assembly and annotation completeness. *Methods Mol Biol*. 2019,1962:227-245.

40. Li H, Durbin R. Fast and accurate short read alignment with Burrows-Wheeler Transform. *Bioinformatics*. 2009,25:1754-1760.

41. Li H, Handsaker B, Wysoker A, Fennell T, Ruan J, Homer N, Marth G, Abecasis G, Durbin R, 1000 Genome Project Data Processing Subgroup. The Sequence Alignment/Map format and SAMtools. Bioinformatics. 2009, 25(16):2078-2079.

42. Ou S, Su W, Liao Y, Chougule K, Agda JRA, Hellinga AJ, et al. Benchmarking transposable element annotation methods for creation of a streamlined, comprehensive pipeline. *Genome Biol.* 2019,20:275.

43. Girgis HZ. Red: an intelligent, rapid, accurate tool for detecting repeats de-novo on the genomic scale. *BMC Bioinform*. 2015,16(1):227.

44. Quinlan AR, Hall IM. BEDTools: a flexible suite of utilities for comparing genomic features. *Bioinformatics*. 2010,26(6):841-842.

45. Camacho C, Coulouris G, Avagyan V, Ma N, Papadopoulos J, Bealer K, et al. BLAST+: architecture and applications. *BMC Bioinform*. 2009,10:421.

46. Cook DE, Valle-Inclan JE, Pajoro A, Rovenich H, Thomma BPHJ, Faino L. Long-Read Annotation: Automated eukaryotic genome annotation based on long-read cDNA sequencing. *Plant Physiol*. 2019,179 (1):38-54.

47. Zhang H, Tanner Y, Huang L, Entwistle S. dbCAN2: a meta server for automated carbohydrate-active enzyme annotation. *Nucleic Acids Res.* 2018,46:W95-W101.

48. Huerta-Cepas J, Forslund K, Coelho LP, Damian PC, Szklarczyk D, Jensen LJ. Fast genome-wide functional annotation through orthology assignment by eggNOG-mapper. *Mol Biol Evol.* 2017,34:2115-2122.

49. The Gene Ontology Consortium. The gene ontology resource: 20 years and still GOing strong. *Nucleic Acids Res.* 2019,47(D1):D330-D338.

50. Ashburner M, Ball CA, Blake JA, Botstein D, Butler H, Cherry JM. Et al. Gene ontology: tool for the unification of biology. *Nat Genet*. 2000,25:25-29.

51. Kanehisa M, Soto Y, Kawashima M, Furumichi M, Tanabe M. KEGG as a reference resource for gene and protein annotation. *Nucleic Acids Res*. 2016,44(D1): D457-D462.

52. Mitchell AL, Attwood TK, Babbitt PC, Blum M, Bork P, Bridge A. et al. InterPro in 2019:

improving coverage, classification and access to protein sequence annotations. *Nucleic Acids Res.* 2019,47(D1):D351-D360.

53. Rawlings ND, Barrett AJ, Thomas PD, Huang XS, Bateman A, Finn RD. The merops database of proteolytic enzymes, their substrates and inhibitors in 2017 and a comparison with peptidases in the PANTHER database. *Nucleic Acids Res*. 2018,46(D1):D624-D632.

54. El-Gebali S, Mistry J, Bateman A, Eddy SR, Luciani A, Potter SC, et al.. The Pfam protein families database in 2019. *Nucleic Acids Res.* 2019,47(D1):D427-D432.

55. The UniProt Consortium. UniProt: a worldwide hub of protein knowledge. *Nucleic Acids Res.* 2019,47(D1):D506-D515.

56. Trincado JL, Entizne JC, Hysenaj G, Singh B, Skalic M, Elliott DJ, Eyras E. SUPPA2: fast, accurate, and uncertainty-aware differential splicing analysis across multiple conditions. *Genome Biol.* 2018,19:40.

57. Zheng Y, Jiao C, Sun H, Rosli HG, Pombo MA, Zhang P, et al. iTAK: a program for genome-wide prediction and classification of plant transcription factors, transcriptional regulators, and protein kinases. *Mol Plant.* 2016,9:1667-1670.

58. Pucker B. Automatic identification and annotation of MYB gene family members in plants. *BMC genom.* 2022,23:220

59. Emms DM, Kelly S. OrthoFinder: solving fundamental biases in whole genome comparisons dramatically improves orthogroup inference accuracy. *Genome Biol.* 2015,16:157.

60. Emms DM, Kelly S. OrthoFinder: phylogenetic orthology inference for comparative genomics. *Genome Biol*. 2019,20:238.

61. Kozlov AM, Darriba D, Flouri T, Morel B, Stamatakis A. RAxML-NG: a fast, scalable and user-friendly tool for maximum likelihood phylogenetic inference. *Bioinformatics*. 2019,35(21):4453-4455.

62. Darriba D, Posada D, Kozlov AM, Stamatakis A, Morel B, Flouri T. ModelTest-NG: A new and scalable tool for the selection of DNA and protein evolutionary models. *Mol Biol Evol*. 2020,37(1):291-294.

63. dos Reis M, Zhu T, Yang Z. The impact of the rate prior on Bayesian estimation of divergence times with multiple Loci. *System Biol*. 2014,63:555-565

64. Han MV, Thomas GWC, Jose LM, Hahn MW. Estimating gene gain and loss rates in the presence of error in genome assembly and annotation using cafe 3. *Mol Biol Evol.* 2013,30(8):1987-1997.

65. Chen CJ, Chen H, Zhang Y, Thomas HR, Frank MH, He YH, Xia R. TBtools- an integrative toolkit developed for interactive analyses of big biological data. *Mol Plant.* 2020,13(8): 1194-1202.

66. Supek F, Bošnjak M, Škunca N, Šmuc T. REVIGO summarizes and visualizes long lists of gene ontology terms. *PLoS One.* 2011,6(7):e21800.

67. Zwaenepoel A, de Peer YV. wgd-simple command line tools for the analysis of ancient whole-genome duplications. *Bioinformatics*. 2019,35:2153-2155.

68. Sensalari C, Maere S, Lohaus R. *Ksrates*: positioning whole-genome duplications relative to speciation events in K_S distributions. *Bioinformatics*. 2022,38(2):530-532.

69. Qiao X, Li QH, Yin H, Qi K, Li L, Wang R, Zhang S, Paterson AH (2019). Gene duplication and evolution in recurring polyploidization–diploidization cycles in plants. *Genome Biol.* 2019,20:38.

70. Yu Y, Ouyang Y, Yao W. shinyCircos: an R/Shiny application for interactive creation of Circos plot. *Bioinformatics*. 2018,34(7):1229-1231.

71. Wang Y, Tang H, Debarry JD, Tan X, Li J, Wang X, et al. MCScanX: a toolkit for detection and evolutionary analysis of gene synteny and collinearity. *Nucleic Acids Res.* 2012,40(7):e49

72. Bandi V, Gutwin C. Interactive exploration of genomic conservation. In Proceedings of the 46th Graphics Interface Conference on Proceedings of Graphics Interface 2020 (GI'20). Canadian Human-Computer Communications Society, Waterloo, CAN. 2020

73. Cabanettes F, Klopp C. D-GENIES: dot plot large genomes in an interactive, efficient and simple way. *PeerJ*. 2018,6:e4958-e4958.

74. Kautsar SA, Duran HGS, Blin K, Osbourn A, Medema MH. plantiSMASH: automated identification, annotation and expression analysis of plant biosynthetic gene clusters. *Nucleic Acids Res*. 2017,45(W1):W55-W63.

75. Töpfer N, Fuchs LM, Aharoni A. (2017) The PhytoClust tool for metabolic gene clusters discovery in plant genomes. *Nucleic Acids Res.* 2017,45(12):7049-7063

76. McHale L, Tan X, Koehl P, Michelmore RW. Plant NBS-LRR proteins: adaptable guards. *Genome Biol.* 2006,7(4):212.

77. Shao Z-Q, Xue J-Y, Wu P, Zhang Y-M, Wu Y, Hang Y-Y, et al. Large-scale analyses of angiosperm Nucleotide-Binding Site-Leucine-Rich Repeat genes reveal three anciently diverged classes with distinct evolutionary patterns. *Plant Physiol.* 2016,170(4):2095-2109.

78. Guo X, Fang D, Sahu SK, Yang S, Guang X, Folk, R. et al. Chloranthus genome provides insights into the early diversification of angiosperms. *Nat Commun.* 2021,12(1): 6930.

79. Steuernagel B, Witek K, Krattinger SG, Ramirez-Gonzalez RH, Schoonbeek HJ, Yu G, et al. The NLR-annotator tool enables annotation of the intracellular immune receptor repertoire. *Plant Physiol.* 2020,183:468-482.

80. Vakirlis N, Sarilar V, Drillon G, Fleiss A, Agier N, Meyniel JP, et al. Reconstruction of ancestral chromosome architecture and gene repertoire reveals principles of genome evolution in a model yeast genus. *Genome Res.* 2016,26(7):918-932.

81. Drillon G, Carbone A, Fischer G. SynChro: a fast and easy tool to reconstruct and visualize synteny blocks along eukaryotic chromosomes. *PLoS One.* 2014,9 (3):e92621.

82. Pucker B, Pandey A, Weisshaar B, Stracke R. The R2R3-MYB gene family in banana (*Musa acuminata*): Genome-wide identification, classification and expression patterns. *PLoS One.* 2020,15(10):e0239275.

83. Busche M, Pucker B, Weisshaar B, Stracke R. Three R2R3-MYB transcription factors from banana (*Musa* spp.) activate structural anthocyanin biosynthesis genes as part of an MBW complex. *BioRxiv*. 2022. https://doi.org/10.1101/2022.08.15.503939.

84. Novák P, Hřibová E, Neumann P, Koblížková A, Doleřel J, Macas J. Genome-wide analysis of repeat diversity across the family Musaceae. *PLoS One*. 2014,9(6):e98918.

85. Bartoš J, Alkhimova O, Doleželová M, De Langhe E, Doležel J. Nuclear genome size and genomic distribution of ribosomal DNA in *Musa* and *Ensete* (Musaceae): taxonomic implications. *Cytogenet Genome Res.* 2005,109(1-3):50-57.

86. Lysák MA, Doleželová M, Horry JP, Swennen R, Doležel J. Flow cytometric analysis of nuclear DNA content in *Musa*. *Theo Appl Genet.* 1999,98:1344-1350.

87. Doležel J, Bartoš J. Plant DNA flow cytometry and estimation of nuclear genome size. *Ann*

Bot. 2005,95(1):99-110.

88. Pellicer J, Powell RF, Leitch IJ. The application of flow cytometry for estimating genome size, ploidy level endopolyploidy, and reproductive modes in plants. *Methods Mol Biol*. 2021,2222:325-361.

89. Van't Ho, J, Sparrow AH. A relationship between DNA content, nuclear volume, and minimum mitotic cycle time. *P Natl Acad Sci USA.* 1963,49:897-902.

90. Šmarda P, Horová L, Bureš P, Hralová I, Marková M. Stabilizing selection on genome size in a population of *Festuca pallens* under conditions of intensive intraspecific competition. *New Phytol.* 2010,187(4):1195-1204.

91. Piegu B, Guyot R, Picault N, Roulin A, Sanyal A, Kim H,et al. Doubling genome size without polyploidization: dynamics of retrotransposition-driven genomic expansions in *Oryza australiensis*, a wild relative of rice. *Genome Res.* 2006,16(10):1262-1269.

92. Macas J, Novák P, Pellicer J, Čížková J, Koblížková A, Neumann P, et al. In depth characterization of repetitive DNA in 23 plant genomes reveals sources of genome size variation in the Legume Tribe Fabeae. *PLoS One.* 2015,10(11):e0143424.

93. Wicker T, Gundlach H, Spannagl M, Uauy C, Borrill P, Ramírez-González RH, et al. Impact of transposable elements on genome structure and evolution in bread wheat. *Genome Biol.* 2018,19(1):103.

94. Novák P, Guignard MS, Neumann P, Kelly LJ, Mlinarec J, Koblížková A, et al. Repeat-sequence turnover shifts fundamentally in species with large genomes. *Nat Plants.* 2020,6(11): 1325-1329.

95. Busche M, Pucker B, Viehöver P, Weisshaar B, Stracke R. Genome sequencing of *Musa acuminata* Dwarf Cavendish reveals a duplication of a large segment of Chromosome 2. *G3-Genes Genom. Genet.* 2020,10 (1):37-42.

96. Chen F, Tholl D, Bohlmann J, Pichersky E. The family of terpene synthases in plants: a mid-size family of genes for specialized metabolism that is highly diversified throughout the kingdom. *Plant J.* 2011,66(1):212-229.

97. Pichersky E, Raguso RA. Why do plants produce so many terpenoid compounds? *New Phytol.* 2016,220(3):655-658.

98. Jiang SY, Jin JJ, Sarojam R, Ramachandran S. A comprehensive survey on the terpene synthase gene family provides new insight into its evolutionary patterns. *Genome Biol Evol.* 2019,11(8):2078-2098.

99. Rojas CM, Senthil-Kumar M, Tzin V, Mysore KS. Regulation of primary plant metabolism during plant-pathogen interactions and its contribution to plant defense. *Front Plant Sci.* 2014,5:17.

100. Meshi T, Iwabuchi M. Plant transcription factors. *Plant Cell Physiol.* 1995,36(8):1405-1420.

101. Amorim LLB, da Fonseca DSR, Neto JPB, Guida-Santos M, Crovella S, Benko-Iseppon AM. Transcription factors involved in plant resistance to pathogens. *Curr Protein Pept Sci.* 2017,18(4):335-351.

102. Gani U, Vishwakarma RA, Misra P. Membrane transporters: the key drivers of transport of secondary metabolites in plants. *Plant Cell Rep.* 2021,40(1):1-18.

103. Pollard M, Beisson F, Li Y, Ohlrogge JB. Building lipid barriers: biosynthesis of cutin and suberin. *Trends Plant Sci.* 2008,13(5):236-246.

104. Wang A, Zha Z, Yin D, Shu X, Ma L, Wang L, et al. Comparative transcriptome analysis of *Tilletia horrida* infection in resistant and susceptible rice (*Oryza sativa* L.) male sterile lines reveals potential candidate genes and resistance mechanisms. *Genomics*, 2020,112(6): 5214-5226.

105. Ziv C, Zhao Z, Gao YG. Xia Y. Multifunctional roles of plant cuticle during plant-pathogen interactions. *Front Plant Sci.* 2018,9:1088.

106. Baales J, Zeisler-Diehl VV, Schreiber L. Analysis of extracellular cell wall lipids: wax, cutin, and suberin in leaves, roots, fruits, and seeds. *Methods Mol Biol.* 2021,2295:275-293.

107. Pighin JA, Zheng H, Balakshin LJ, Goodman IP, Western TL, Jetter, R, et al. Plant cuticular lipid export requires an ABC transporter. *Science*. 2004,306(5696):702-704.

108. Elejalde-Palmett C, Segundo IMS, Garroum I, Charrier L, De Bellis D, Mucciolo A, et al. ABCG transporters export cutin precursors for the formation of the plant cuticle. *Curr Biol.* 2021,31(10):2111-2123.e9

109. Kang J, Park J, Choi H, Burla B, Kretzschmar T, Lee Y, Martinoia E. Plant ABC Transporters. *Arabidopsis Book*, 2011,9:e0153.

110. Bailly, A. Structure-function of plant ABC-Transporters. In: Geisler M editor. Plant ABC Transporters. Cham: Springer; 2014. p. 219-240.

111. Do THT, Martinoia E, Lee Y. Functions of ABC transporters in plant growth and development. *Curr Opin Plant Biol.* 2018,4:32-38.

112. Banasiak J, Jasiński M. (2022). ATP-binding cassette transporters in nonmodel plants. *New Phytol*. 2022,233:1597-1612.

113. Winkel-Shirley B. Flavonoid biosynthesis. A colorful model for genetics, biochemistry, cell biology, and biotechnology. *Plant Physiol*. 2001,126(2):485-93.

114. Pucker B, Reiher F, Schilbert HM. Automatic identification of players in the flavonoid biosynthesis with application on the biomedicinal plant *Croton tiglium*. *Plants (Basel)*. 2020,9(9):1103.

115. Liu W, Feng Y, Yu S, Fan Z, Li X, Li J, et al. The flavonoid biosynthesis network in plants. *Int J Mol Sci*. 2021,22:12824.

116. Shen N, Wang T, Gan Q, Liu S, Wang Li, Jin B. Plant flavonoids: Classification, distribution, biosynthesis, and antioxidant activity. *Food Chem*. 2022,383:132531.

117. Pandey A, Alok A, Lakhwani D, Singh J, Asif MH, Trivedi PK. Genome-wide expression analysis and metabolite profiling elucidate transcriptional regulation of flavonoid biosynthesis and modulation under abiotic stresses in Banana. *Sci Rep*. 2016,6:31361.

118. Sun X, Gao P, Zhang J, Xu B, Jin Z, Liu J (2018) Characteristics of flavonoids biosynthesis and the differential expression analysis of the key enzyme genes in *Musa* AAA Group cv Brazilian fruit pulp. *Mol Plant Breeding*. 2018,16(7):2116-2123.

119. Murata K, Kitano T, Yoshimoto R, Takata R, Ube N, Ueno K, et al. Natural variation in the expression and catalytic activity of a naringenin 7-O-methyltransferase influences antifungal defenses in diverse rice cultivars. *Plant J*. 2019,101(5):1103-1117.

120. Busche M, Acatay C, Martens S, Weisshaar B, Stracke R. Functional characterisation of Banana (*Musa* spp.) 2-Oxoglutarate-Dependent Dioxygenases involved in flavonoid biosynthesis. *Front Plant Sci*. 2021,12:701780.

121. Petrussa E, Braidot E, Zancani M, Peresson C, Bertolini A, Patui S, Vianello A. Plant flavonoids—biosynthesis, transport and involvement in stress responses. *Int J Mol Sci* 2013,14:14950-14973.

122. Pucker B, Selmar D. Biochemistry and molecular basis of intracellular flavonoid transport in plants. *Plants*. 2022,11:963.

123. Pazmiño-Durán EA, Giusti MM, Wrolstad RE, Glória MBA. Anthocyanins from banana bracts (*Musa* X *paradisiaca*) as potential food colorants. *Food Chem*. 2001,73(3):327-332.

124. Fu X, Cheng S, Liao Y, Huang B, Du B, Zeng W, et al. Comparative analysis of pigments in red and yellow banana fruit. *Food Chem*. 2018,239:1009-1018.

125. Deng S, Cheng C, Liu Z, Chen Y, Zhang Z, Huang Y, et al. Comparative transcriptome analysis reveals a role for anthocyanin biosynthesis genes in the formation of purple peel in Minhou wild banana (*Musa itinerans* Cheesman). *J Hortic Sci Biotechnol*. 2019,94(2):184-200.

126. Jiao F, Zhao L, Wu X, Song Z, Li Y. Metabolome and transcriptome analyses of the molecular mechanisms of flower color mutation in tobacco. *BMC Genom*. 2020,21:611.

127. Zhang X, Lin S, Peng D, Wu Q, Liao X, Xiang K, et al. Integrated multi-omic data and analyses reveal the pathways underlying key ornamental traits in carnation flowers. *Plant Biotechnol. J*. 2022,20(6):1182-1196.

128. Nützmann H-W, Huang A, Osbourn A. (2016). Plant metabolic clusters – from genetics to genomics. *New Phytol*. 2016,211(3):771-789.

129. Polturak G, Osbourn A. The emerging role of biosynthetic gene clusters in plant defense and plant interactions. *PLoS Pathog*. 2021,17(7):e1009698.

130. Yu D, Xu F, Zeng J, Zhan J. Type III polyketide synthases in natural product biosynthesis. *IUBMB Life*, 2012,64(4):285-295.

131. Flores-Sanchez IJ, Verpoorte R. Plant polyketide synthases: A fascinating group of enzymes. *Plant Physiol Biochem*. 2009,47(3):167-174.

132. Mhlanga M. Plant polyketides. *Nat Biotechnol*. 1999,17:9.

133. Rajesh T, Tiwari MK, Thiagarajan S, Nair PS, Jeya M. Type III polyketide synthases: Current state and perspectives. In: Arora P, editor. Microbial Technology for the Welfare of Society. Singapore: Springer; 2019. p. 183-200

134. Pothiraj R, Ravikumar MJ, Suthanthiram B, Subbaraya U, Krishnamurthy P. Genome-scale analyses of polyketide synthases in banana: Phylogenetics and expression profiling forecast their candidacy in specialized metabolism. *Gene.* 2021,778:145472.

135. Gröger D. Terpenoid and steroid alkaloids. In: Constabel F, Vasil IK editors. Phytochemicals in Plant Cell Cultures. Academic Press; 1988. p. 435-448.

136. Piasecka A, Jedrzejczak-Rey N, Bednarek P. Secondary metabolites in plant innate immunity: conserved function of divergent chemicals. *New Phytol.* 2015,206(3):948-964.

137. Hoagland RE. Toxicity of tomatine and tomatidine on weeds, crops and phytopathogens fungi. *Allelopathy J.* 2009,23(2):425-435.

138. Nakayasu M, Akiyama R, Kobayashi M, Lee HJ, Kawasaki T, Watanabe B, et al. Identification of α-Tomatine 23-Hydroxylase involved in the detoxification of a bitter glycoalkaloid. *Plant Cell Physiol.* 2020,61(1):21-28.

139. Itkin M, Rogachev I, Rogachev I, Alkan N, Rosenberg T, Malitsky S, et al. GLYCOALKALOID METABOLISM1 is required for steroidal alkaloid glycosylation and prevention of phytotoxicity in tomato. *Plant Cell.* 2011,23(12):4507-4525.

140. Itkin M, Heinig U, Tzfadia O, Bhide AJ, Shinde B, Cardenas PD, et al. Biosynthesis of antinutritional alkaloids in solanaceous crops is mediated by clustered genes. *Science.* 2013,341(6142):175-179.

141. You Y, van Kan JAL. Bitter and sweet make tomato hard to (b)eat. *New Phytol.* 2020,230(1):90-100.

142. Cruz-Cruz CA, Ramírez-Tec G, García-Sosa K, Escalante-Erosa F, Hill L, Osbourn AE, et al. Phytoanticipins from banana (*Musa acuminata* cv. Grande Naine) plants, with antifungal activity against *Mycosphaerella fijiensis*, the causal agent of black Sigatoka. *Eur J Plant Pathol.* 2010,126(4):459-463.

143. Soares JMS, Rocha AJ, Nascimento FS, Santos AS, Miller RNG, Ferreira CF, et al. Genetic improvement for resistance to Black Sigatoka in Bananas: a systematic review. *Front Plant Sci.* 2021,12:657916.

144. Gachon CM, Langlois-Meurinne M, Saindrenan P. Plant secondary metabolism glycosyltransferases: the emerging functional analysis. *Trends Plant Sci.* 2005,10(11):542-549.

145. Lairson LL, Henrissat B, Davies GJ, Withers SG. Glycosyltransferases: structures, functions, and mechanisms. *Annu Rev Biochem.* 2008,77:521-555.

146. He B, Bai X, Tan Y, Xie W, Feng Y, Yang G-Y. Glycosyltransferases: Mining, engineering and applications in biosynthesis of glycosylated plant natural products. *Synth Syst Biotechnol.* 2022,7:602-620.

147. Wang J, Hou B-K. Glycosyltransferases: key players involved in the modificationof plant secondary metabolites. *Front Biol China.* 2009,4(1):39-46.

148. Phan JL, Tucker MR, Khor SF, Shirley N, Lahnstein J, Beahan C, et al. Differences in glycosyltransferase family 61 accompany variation in seed coat mucilage composition in *Plantago* spp. *J Exp Bot.* 2016,67(22):6481-6495.

149. Cenci A, Chantret N, Rouard M. Glycosyltransferase family 61 in Liliopsida (Monocot): The story of a gene family expansion. *Front Plant Sci.* 2018,9:1843.

150. Rennie EA, Scheller HV. Xylan biosynthesis. *Curr Opin Biotech.* 2014,26:100-107.

151. Yu J, Hu F, Dossa K, Wang Z, Ke T. Genome-wide analysis of UDP-glycosyltransferase super family in *Brassica rapa* and *Brassica oleracea* reveals its evolutionary history and functional characterization. *BMC Genom.* 2017,18(1):474.

152. Hou J, Ye N, Dong ZY, Lu MZ, Li LG, Yin TM. Major chromosomal rearrangements distinguish willow and poplar after the ancestral "Salicoid" genome duplication. *Genome Biol Evol.* 2016,8:1868-1875.

153. Susek K, Bielski WK, Hasterok R, Naganowska B, Wolko B. A first glimpse of wild lupin karyotype variation as revealed by comparative cytogenetic mapping. *Front Plant Sci.* 2016,7:1152.

154. Ma X, Vaistij FE, Li Y, van Rensburg WSJ, Harvey S, Bairu MW, et al. A chromosome-level Amaranthus cruentus genome assembly highlights gene family evolution and biosynthetic gene clusters that may underpin the nutritional value of this traditional crop. *Plant J.* 2021,107:613-628.

155. Bennetzen JL. Transposable elements, gene creation and genome rearrangement in flowering plants. *Curr Opin Genet Dev.* 2005,15(6):621-627.

156. Kalendar R, Sabot F, Rodriguez F, Karlov GI, Natali L, Alix K. Editorial: Mobile elements and

plant genome evolution, comparative analyzes and computational tools. *Front. Plant Sci.* 2021,12:735134.

157. Lysák MA, Schubert I. Mechanisms of chromosome rearrangements. In: Greilhuber J, Dolezel J, Wendel J, editors. Plant Genome Diversity Volume 2. Vienna: Springer; 2013. p. 137-147

158. Barra V, Fachinetti D. The dark side of centromeres: types, causes and consequences of structural abnormalities implicating centromeric DNA. *Nat Commun.* 2018,9(1):4340.

159. Manova V, Gruszka D. DNA damage and repair in plants –from models to crops. *Front Plant Sci.* 2015,6:885.

160. Wood RD. DNA repaired in eukaryotes. *Annu Rev Biochem*. 1996,65:135-167.

161. Nisa M-U, Huang Y, Benhamed M, Raynaud C. The plant DNA damage response: signaling pathways leading to growth inhibition and putative role in response to stress conditions. *Front Plant Sci*. 2019,10:653.

Table 1. Statistics of genome assembly for *Musa beccarii*

Contig statistics of initial assembly using Nanopore reads		Contig statistics of initial assembly using PacBio HiFi reads		Scaffold statistics after Hi-C scaffolding		Chromosome	Length
The length of sequence (bp)	The order of sequence length	The length of sequence (bp)	The order of sequence length	The length of sequence (bp)	The order of sequence length		
N10=48080317	L10=2	N10=8700004	L10=7	N10=79885826	L10=1	chr1	79367759
N20=39700570	L20=3	N20=5180184	L20=17	N20=79367759	L20=2	chr2	79885826
N30=27992656	L30=5	N30=3933706	L30=31	N30=73517995	L30=3	chr3	67088101
N40=21895089	L40=7	N40=3192498	L40=48	N40=73517995	L40=3	chr4	57442642
N50=18949966	L50=11	N50=2546178	L50=70	N50=67088101	L50=4	chr5	73517995
N60=15652116	L60=14	N60=2007927	L60=99	N60=60040564	L60=5	chr6	60040564
N70=12145816	L70=18	N70=1507786	L70=136	N70=57442642	L70=6	chr7	53040366
N80=8091256	L80=25	N80=1059843	L80=186	N80=53040366	L80=7	chr8	42891246
N90=1849914	L90=40	N90=527812	L90=271	N90=42891246	L90=8	chr9	38409407
N100=21817	L100=306	N100=12368	L100=811	N100=1000	L100=449		
Total length	607623222bp	636694734bp		569617942bp			
Average length	1985696.80bp	785073.65bp		1268636.84bp			
Largest length	52524701bp	11573678bp		79885826bp			
Minimum length	21817bp	12368bp		1000bp			

Table 2. Possible biosynthetic gene clusters identified in Musaceae species. Tomatine 1 and 2 are tomatine clusters that locate in different chromosomes when previously identified

Cluster	*M. beccarii*	*E. glaucum*	*M. balbisiana*	*M. acuminata*	*M. schizocarpa*
Saccharide	2	3	3	2	3
Solanum l tomatine 1	11	7	10	13	9
Solanum l tomatine 1- Saccharide	0	1	0	1	1
Solanum l tomatine 1-Tomatine 2	2	1	2	2	3
T3PKS	21	17	18	22	14
T3PKS-Saccharide	2	1	0	1	1
T3PKS-Solanum l tomatine 1	6	8	9	9	11
T3PKS-Solanum l tomatine 1-Tomatine 2	3	1	0	2	3
T3PKS-Terpene	0	0	1	1	0
T3PKS-Terpene-Solanum l tomatine 1	1	2	1	1	1
T3PKS-Tomatine 2	2	2	3	1	0
Terpene	3	6	8	8	7
Terpene-Solanum l tomatine 1	3	5	2	2	2
Terpene-Solanum l tomatine 1-Tomatine 2	1	0	1	0	0
Tomatine 2	8	4	6	6	6
Other	1	0	0	1	0
Other-Solanum l tomatine 1-Tomatine 2	0	1	0	0	1
Total	66	59	64	72	62

Figure legends

Figure 1. Picture showing *Musa beccarii* flower

Figure 2. A) Hi-C interaction heat map (bin length 10,000 bp) for the *Musa beccarii* genome; B) Genome features across the chromosomes of *M. beccarii*. C) Inferred phylogenetic tree and contracted (–) and expanded (+) gene family in *M. beccarii* and other species in Liliopsida. The total gene families within the most recent common ancestor (MRCA) are denoted at the root. Numbers following each species are the statistics of different genes

Figure 3. A) Number and B) length of transposable elements in Musaceae species. LTR: long terminal repeats; TIR: terminal inverted repeats; nonTIR: non-terminal inverted repeats

Figure 4. A) Density distribution of synonymous nucleotide substitutions (*Ks*) in whole genome duplication analysis; B) Speciation event (red line) detection using rate-adjusted *Ks* distribution for *Musa beccarii* applied by *ksrate* package. The background is whole-paranome *Ks* distribution (light gray histogram and KDE curve) and anchor-pair *Ks* distribution (dark gray histogram and KDE curve) for *M. beccarii*. The shared number in the red circle indicates the same speciation event between *M. beccarii* and the other *Musa* species. The numbers and arrows in the parentheses of four *Musa* species in panel legend indicate *Ks* value shifts after *ksrates*' substitution rate adjustments; C) Synteny blocks between Musaceae species. The largest blocks in *Musa* are highlighted orange color; D) Biosynthetic gene clusters in chr4 in *M. beccarii* and the gene synteny with the other threes *Musa* species in their chr4s. Note in the BGCs, the regulatory genes are not shown

Figure 5. Dot plots of *Musa beccarii* and the other four species in Musaceae, visualized using D-GENIES. The dot colors correspond to similarity values that are binned in four groups. Highly conserved chromosomes between *M. beccarii* and the other *Musa* genomes are highlighted in red boxes

Figure 6. Chromosomal history of the Musaceae genomes shows genome structure changing from the last common ancestor (LCA, the most above genome in the picture) to three intermediate ancestors and five studied species. The genes in the LCA are represented with lines and the same colors if they are in the same contigs except for the genes in the contigs contained gene numbers smaller than 100, which are stacked into one super contig and all colored with black. The genes in the intermediate ancestors and studied species are colored with respect to the LCA orthologous genes (determined by reciprocal best hits in SynChro analysis) and otherwise colored with white to reflect the lack of homology to LCA genes

Additional Files

Supplementary Table S1. Libraries for genome assembly and annotation for *Musa beccari*

Supplementary Table S2. Protein sequences of three species used for gene prediction

Supplementary Table S3. Species used for comparative genomics

Supplementary Table S4. Species pairs and their estimated divergence times used for time calibration points to infer time-calibrated phylogeny of *Musa beccarii*

Supplementary Table S5. Genome size (bp) estimation using different programs

Supplementary Table S6. Assembly results using different assemblers

Supplementary Table S7. Repeat content of assemblies of Musaceae

Supplementary Table S8. Summary of gene functional annotations of the *Musa beccarii* genome using different databases

Supplementary Table S9. Summary of genes in Musaceae and the other compared species

Supplementary Table S10. Summary of MYB transcription factors identified by MYB_annotator, and their comparison with OrthoFinder results

Supplementary Table S11. MYB genes identified by MYB_annotator in *Musa beccarii*

Supplementary Table S12. Summer of MYB gene functions

Supplementary Table S13. Statistics of gene families in different species

Supplementary Table S14. GO enrichment results for Musaceae specific gene families in *Musa beccarii*

Supplementary Table S15. KEGG enrichment results for Musaceae specific gene families in *Musa beccarii*

Supplementary Table S16. GO enrichment results for significantly expanded gene families

Supplementary Table S17. KEGG enrichment results for signifcantly expanded gene families

Supplementary Table S18. GO enrichment results for significantly contracted gene families

Supplementary Table S19. KEGG enrichment results for signifcantly contracted gene families

Supplementary Table S20. GO enrichment results for alternative splicing genes

Supplementary Table S21. KEGG enrichment results for alternative splicing genes

Supplementary Table S22. GO enrichment results for WGD type genes

Supplementary Table S23. KEGG enrichment results for WGD type genes

Supplementary Table S24. GO enrichment results for TD type genes

Supplementary Table S25. KEGG enrichment results for TD type genes

Supplementary Table S26. GO enrichment results for PD type genes

Supplementary Table S27. KEGG enrichment results for PD type genes

Supplementary Table S28. GO enrichment results for TRD type genes

Supplementary Table S29. GO enrichment results for DSD type genes

Supplementary Table S30. KEGG enrichment results for DSD type genes

Supplementary Table S31. Results of the syntenic block analysis

Supplementary Table S32. Biosynthetic gene clusters in Musaceae

Supplementary Table S33. NLR-Annotator results in *Musa beccarii*

Supplementary Table S34. NLR-Annotator results in Musaceae using their predicted genes and comparison of the results using the genomes

Supplementary Table S35. Structure variations (SVs) detected by SVanalyzer between *Musa beccarii* and *M. acuminata*

Supplementary Table S36. Summary of ABC transporter related genes

Supplementary Table S37. Summary of glycosyl transferases genes

Supplementary Figure S1. A scheme showing *Musa beccarii* genome assembly

Supplementary Figure S2. A) Nanica repetitive sequences and B) NBS-LRR genes identified by

NLR-annotator across the *Musa beccarii* genome

Supplementary Figure S3. Treemap showing hierarchy for GO terms enriched for Musaceae-specific gene families of *Musa beccarii* in biological process

Supplementary Figure S4. Upset plot showing the intersection of the gene family in *Musa*. *Musa* species are presented in rows and the bar beside each species shows its total gene number. Black circles and vertical lines between the rows represent the intersection of gene families between species. Barplot indicates the total gene family count in each intersection

Supplementary Figure S5. Summary of GO annotations of Musaceae shared genes

Supplementary Figure S6. Treemap showing hierarchy for GO terms enriched with significantly expanded gene families in biological process

Supplementary Figure S7. Treemap showing hierarchy for GO terms enriched with alternative splicing genes in biological process

Supplementary Figure S8. Treemap showing hierarchy for GO terms enriched with genes related to WGD in biological process

Supplementary Figure S9. KEGG enrichment for genes related to A) whole genome duplication; B) tandem duplications; C) proximal duplications; D) dispersed duplications tested by DupGen_Finder

Supplementary Figure S10. Treemap showing hierarchy for GO terms enriched with genes related to tandem duplications in biological process

Supplementary Figure S11. Treemap showing hierarchy for GO terms enriched with genes related to dispersed duplications in biological process

Supplementary Figure S12. Synteny plot (Synvisio) between *Ensete glaucum* (egxx) and *Musa acuminata* (mpxx), *Musa beccarii* (bexx) genomes. Syntenic blocks of high homology are indicated by uniformly colored areas in the graphs

Supplementary Figure S13. Coverage distributions of Illumina WGS reads mapping against *Musa beccarii* assembly

Supplementary Figure S14. Histograms showing allele frequencies of SNVs (single nucleotide variants) obtained from the Illumina WGS reads mapping against *Musa beccarii* assembly

Supplementary Figure S15. KEGG pathway of flavonoid biosynthesis. In the pathway, the genes

identified in *Musa beccarii* are boxed in green or pink colors.

Figure_1

Click here to access/download;Figure;Figure_1.tif

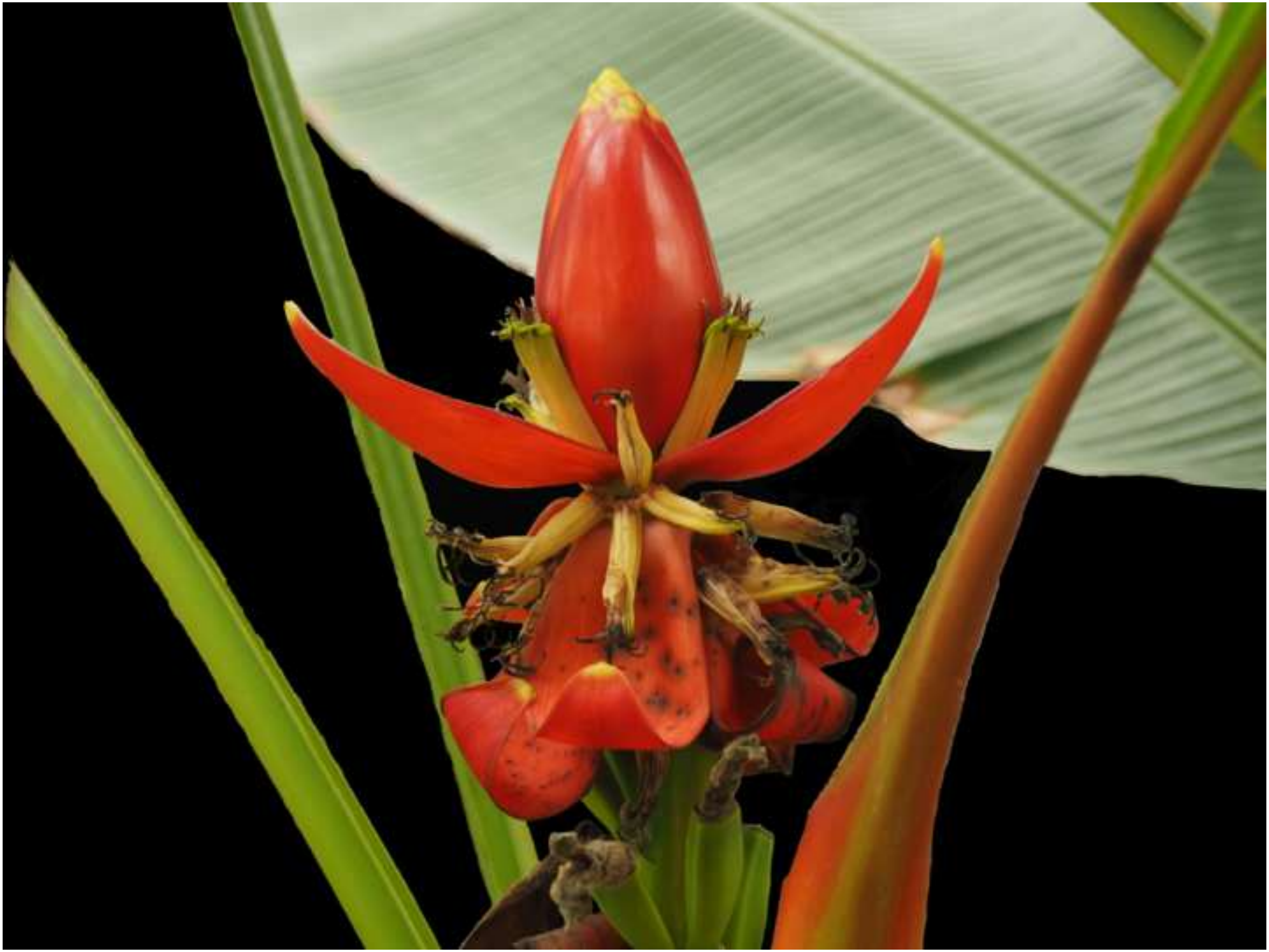

Figure_2

Click here to access/download;Figure;Figure_2.tif

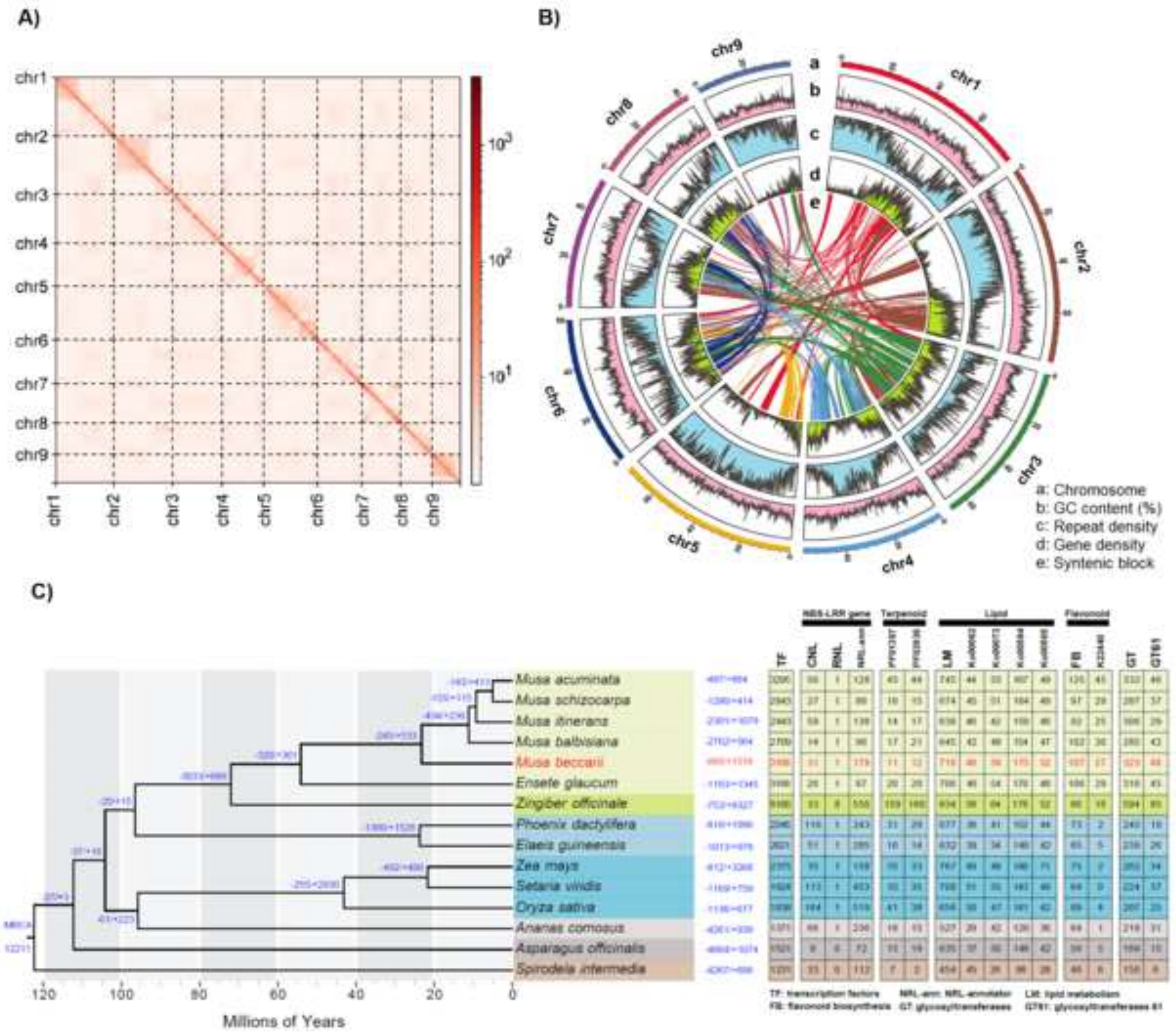

Figure_3

Click here to access/download;Figure;Figure_3.tif

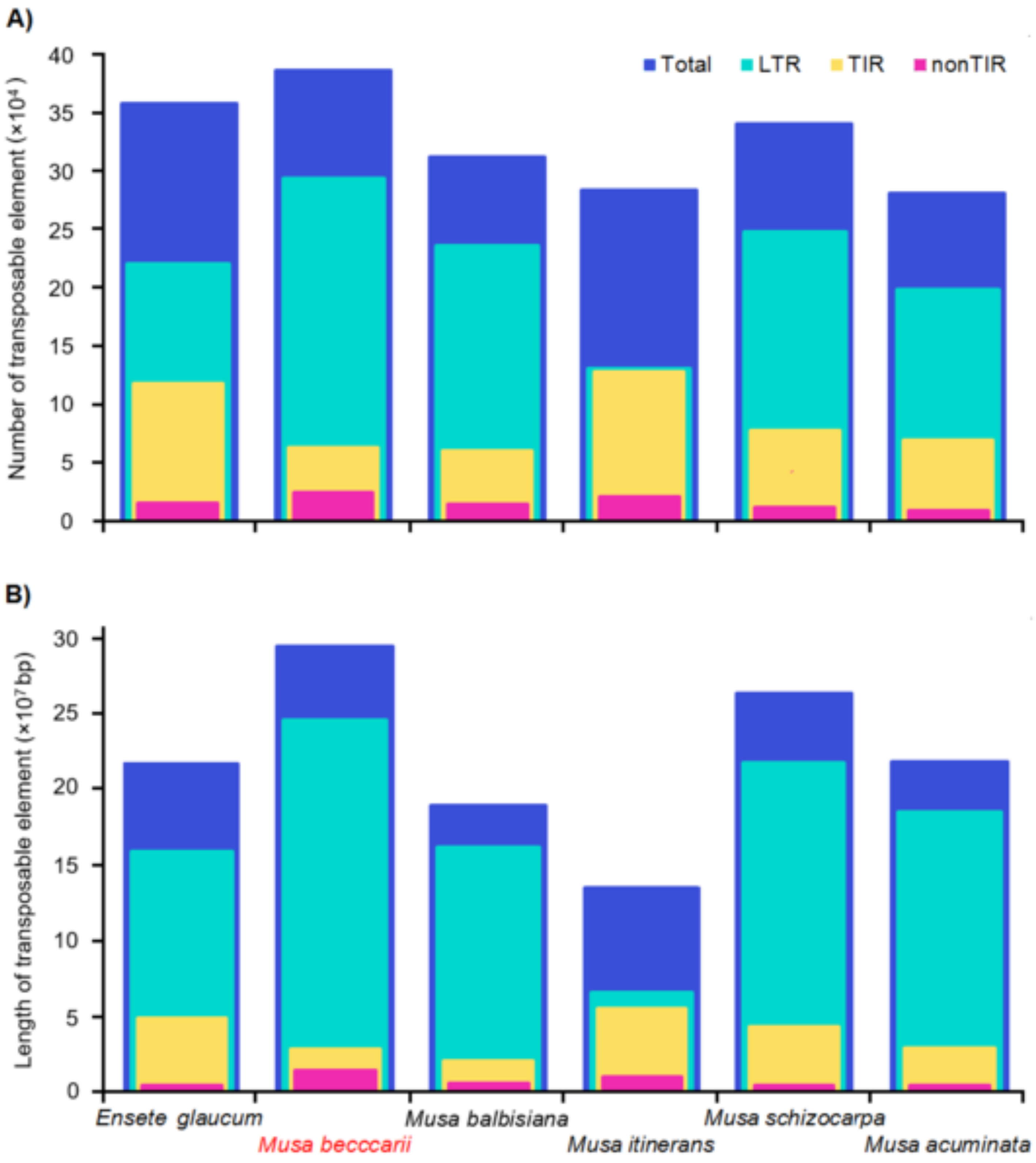

Figure_4

Click here to access/download;Figure;Figure_4.tif

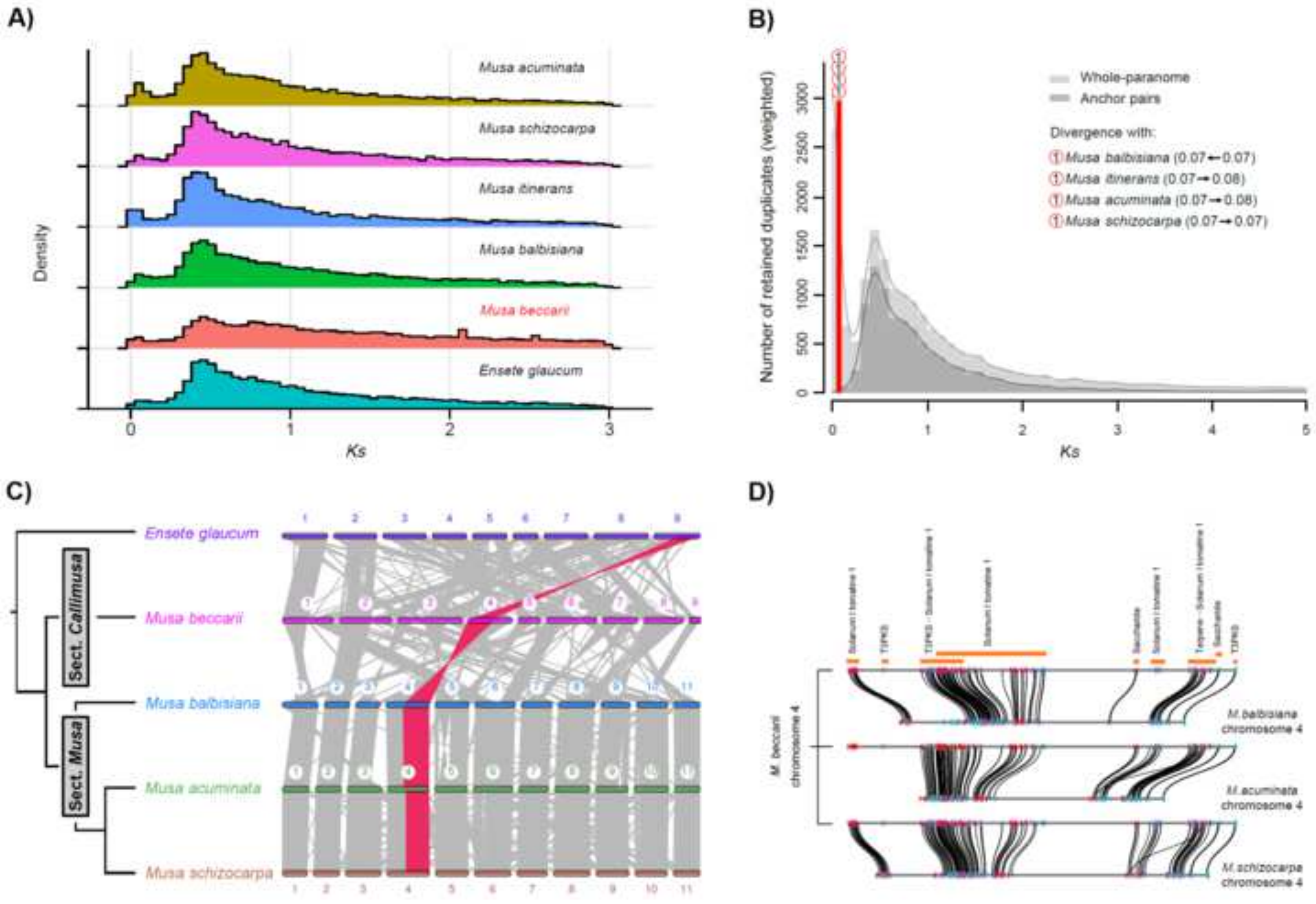

Figure_5

Click here to access/download;Figure;Figure_5.tif

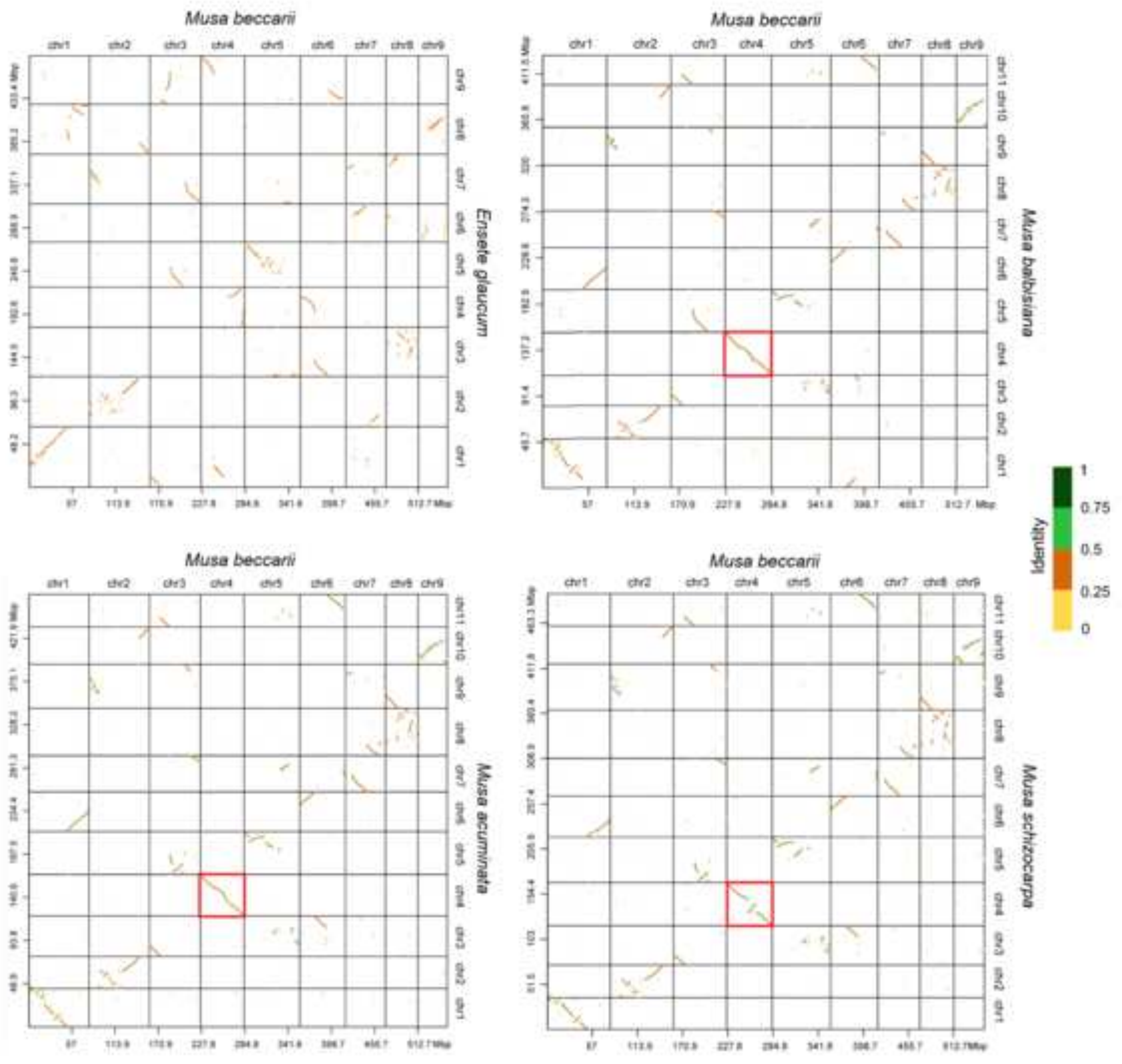

Figure_6

Click here to access/download;Figure;Figure_6.tif

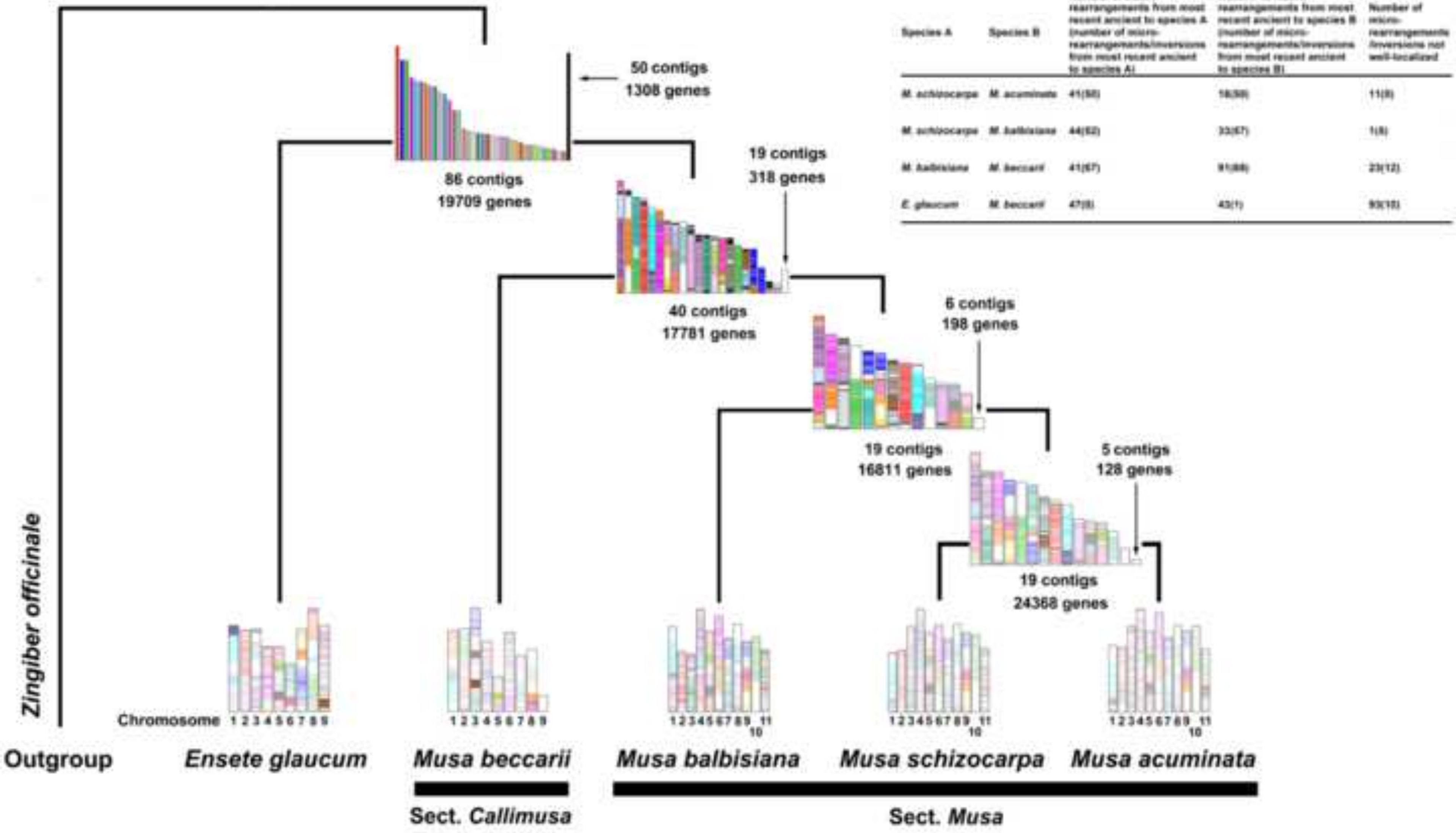

Species A	Species B	Number of macro-rearrangements from most recent ancient to species A (number of micro-rearrangements/inversions from most recent ancient to species A)	Number of macro-rearrangements from most recent ancient to species B (number of micro-rearrangements/inversions from most recent ancient to species B)	Number of micro-rearrangements /inversions not well-localized
M. schizocarpa	*M. acuminata*	41(50)	18(30)	11(8)
M. schizocarpa	*M. balbisiana*	44(52)	33(67)	1(8)
M. balbisiana	*M. beccarii*	41(67)	91(88)	23(12)
E. glaucum	*M. beccarii*	47(8)	43(1)	93(10)

Click here to access/download
Supplementary Material
ms-revised-tracked.docx

Figure_S1

Click here to access/download
Supplementary Material
Figure_S1.tif

Figure_S2

Click here to access/download
Supplementary Material
Figure_S2.tif

Figure_S3

Click here to access/download
Supplementary Material
Figure_S3.pdf

Figure_S4

Click here to access/download
Supplementary Material
Figure_S4.tiff

Figure_S5

Click here to access/download
Supplementary Material
Figure_S5.tif

Figure_S6

Click here to access/download
Supplementary Material
Figure_S6.pdf

Figure_S7

Click here to access/download
Supplementary Material
Figure_S7.pdf

Figure_S8

Click here to access/download
Supplementary Material
Figure_S8.pdf

Figure_S9

Click here to access/download
Supplementary Material
Figure_S9.tiff

Figure_S10

Click here to access/download
Supplementary Material
Figure_S10.pdf

Figure_S11

Click here to access/download
Supplementary Material
Figure_S11.pdf

Figure_S12

Click here to access/download
Supplementary Material
Figure_S12.tif

Figure_S13

Click here to access/download
Supplementary Material
Figure_S13.tif

Figure_S14

Click here to access/download
Supplementary Material
Figure_S14.tif

Figure_S15

Click here to access/download
Supplementary Material
Figure_S15.png

Supplymentary_file_1

Click here to access/download
Supplementary Material
Supplymentary_file_1.docx

Supplymentary_tableS1-S36

Click here to access/download
Supplementary Material
supplymentary_tableS1-S36.xlsx
