# Supplementary material for: Genome assembly of Musa beccarii shows extensive chromosomal rearrangements and genome expansion during evolution of Musaceae genomes
Source: Gigascience. 2023 Feb 21;12:giad005. doi: 10.1093/gigascience/giad005 (PMC9941839; doi:10.1093/gigascience/giad005)
Supplement: giad005_GIGA-D-22-00219_Revision_2 [file giad005_giga-d-22-00219_revision_2.pdf]

GigaScience

Genome assembly of Musa beccarii shows extensive chromosomal rearrangements and genome expansion during evolution of Musaceae genomes

--Manuscript Draft--

**Manuscript Number:**	GIGA-D-22-00219R2
**Full Title:**	Genome assembly of Musa beccarii shows extensive chromosomal rearrangements and genome expansion during evolution of Musaceae genomes
**Article Type:**	Data Note
**Funding Information:**	
**Abstract:**	Background: Musa beccarii (Musaceae) is a banana species native to Borneo, and it is sometimes grown as an ornamental plant. The basic chromosome number of Musa species is x=7, 10, or 11; however, M. beccarii has a basic chromosome number of x=9 (2n=2x=18), which is the same basic chromosome number of species in the sister genera Ensete and Musella. M. beccarii is in the section Callimusa, which is sister to the section Musa. We generated a high-quality chromosome-scale genome assembly of M. beccarii to better understand the evolution and diversity of genomes within the family Musaceae<br>Findings: The M. beccarii genome was assembled by long-read and Hi-C sequencing, and genes were annotationed using both long Iso-Seq and short RNA-seq reads. The size of the M. beccarii was the largest mong all known Musaceae assemblies (~570 Mbp), and this stems from the expansion of transposable elements and increases in the number of 45S rDNA sites. We detected extensive genome-wide chromosome fusions and fissions between M. beccarii and the other Musa and Ensete species by synteny analysis, far beyond those expected from differences in chromosome number. Within Musaceae, M. beccarii showed a reduced number of terpenoid synthase genes which are related to chemical defense and enrichment in lipid metabolism genes linked to the physical defense of the cell wall. Furthermore, type III polyketide synthase (T3PKS) was the most abundant biosynthetic gene cluster (BGCs) in M. beccarii. BGCs were not conserved in Musaceae genomes.<br>Conclusions: The genome assembly of M. beccarii is the first chromosome-scale genome assembly in the Callimusa section in Musa, which provides an important genetic resource that aids our understanding of the evolution of Musaceae genomes and enhances our knowledge of the pangenome.
**Corresponding Author:**	Xue-Jun Ge<br>South China Botanical Garden<br>Guangzhou, Guangdong CHINA
**Corresponding Author Secondary Information:**	
**Corresponding Author's Institution:**	South China Botanical Garden
**Corresponding Author's Secondary Institution:**	
**First Author:**	Zheng-Feng Wang
**First Author Secondary Information:**	
**Order of Authors:**	Zheng-Feng Wang
	Mathieu Rouard
	Gaetan Droc
	Pat (J.S.) Heslop-Harrison
	Xue-Jun Ge
**Order of Authors Secondary Information:**	
**Response to Reviewers:**	November 19, 2022<br>GigaScience

Powered by Editorial Manager® and ProduXion Manager® from Aries Systems Corporation

Dear editor,

Thank you very much for your decision letter about our manuscript entitled “Genome assembly of Musa beccarii shows extensive chromosomal rearrangements and genome expansion during evolution of Musaceae genomes”(No. GIGA-D-22-00219R1), including the comments.
We are now sending our responses to comments with our revised manuscript (both tracked and clean versions). All the modified parts in the revised manuscript are marked in red.

Our specific responses are as follows:

Reviewer(s)' Comments to Author:

Reviewer #1:

The authors addressed almost all of my comments very well. I see an overall improvement of the manuscript. There are only a few remaining points:

1) "assembled genome" > "genome sequence"
>>>We have corrected them throughout the text.

3) Unfortunately, this was not clarified through the changes and is not correct yet:
"PacBio full-length RNA transcripts sequencing" > "PacBio full-length cDNA sequencing"
"short-read RNA transcripts sequencing" > "short-read cDNA fragment sequencing"
>>>We have corrected them. Please see lines 127-128.

4) What about the growth conditions (temperature, humidity, soil, ...)? As many details as possible should be provided to make the data sets suitable for re-use.
>>>We have enlarge the paragraph to include this information. Please see lines 109-119.

5) It would be important to include additional information about the ONT basecalling. I would also recommend to include all these sequencing method descriptions in the main document and not in a supplementary file. There are different font sizes/types used in this document (can be ignored if moved to the main document).
>>>We have included the ONT basecalling information, please see lines 185-187. The methods are now in the text and the supplymentary file 1 is deleted, please see lines 129-172.

after examination, the RNA was synthesized into cDNA > after examination, the RNA was reverse transcribed into cDNA
>>>We have rephrased that. Please see line 166.

7) I am not able to find the expression "properly mapped" in that sentence. It seems that only the percentage of mapped reads was considered. Was no filtering for multi-mapped reads and no filtering based on alignment scores applied? This sentence could benefit from some additional explanations.
>>>We removed “properly mapped” from our initial version of the manuscript because of its wrong expression. The followed question that the reviewer mentioned could not be obtained by the results from “flagstat” command of SAMtools. We now run SQUAT (Yang et al. 2019), a sequence assembly quality assessment tool, to include this information. Through the mapped reads, SQUAT categorizes reads into groups of uniquely mapped reads, multiply mapped, unmapped reads. For uniquely mapped reads, it further categorizes them into perfectly matched, with substitutions, containing clips, and the others. Please see lines 213-220, 379-384.

Reference:
Yang L-A, Chang Y-J, Chen S-H, Lin C-Y. Ho J-M. SQUAT: a Sequencing Quality Assessment Tool for data quality assessments of genome assemblies. BMC Genom. 2019,19(Suppl 9):238

9) Thanks for the update. Unfortunately, I cannot find the BUSCO config file there. It would be interesting to know the e-value cutoffs and the number of genes to consider etc. These settings can influence the completeness score. My initial impression was

that BUSCO was used to assess the completeness of the annotation. However, the log file suggest that BUSCO was run in the genome mode i.e. ignored the annotation and started with a gene prediction from scratch.

>>>We used BUSCO v5.2.2 and installed it via CONDA. When running the program, we did not use a config file and the program was then run under its default parameters setting. The config file is also not generated after running the program, but the settings were shown in a running log file. The e-value in the default setting is 0.001. We now upload the log file for reviewing (please see https://doi.org/10.6084/m9.figshare.19165280.v13).
We run BUSCO in two models, genome and protein model ("--mode prot" in which we used longest transcripts, 39,112 protein sequences). In the text, we provide the BUSCO gene model result (please see lines 419-421), but not indicated it in "Materials and Methods", we now include the information in the text. Please see lines 248-250. We also upload the gene model results into the website of "figshare" for reviewing (please see https://doi.org/10.6084/m9.figshare.19165280.v13).

11) The last part about LASTAL is not clear, but it seems that the authors have considered synteny.

>>>It should be the program "LAST" (https://gitlab.com/mcfrith/last). The command usage is "lastal …", we mislead that.

12) This addition is helpful. What was the specific p-value cutoff or any other cutoff used to define 'significantly enriched'?

>>>We have included this information. Please see lines 291-292.

X1) Supplementary Fig1: Illustrating the process is very important, but I have some questions about some aspects:

a) Why was the removal of duplicates performed multiple times in the HiFi branch of the process? Duplicates removed at the contig level should not become an issue after the scaffolding.

>>>Thank you. We actually did not compare the scaffolding results between removing and non-removing the duplications in the contigs. In our study, we used Pseudohaploid (https://github.com/schatzlab/pseudohaploid) and Purge_Dups (Guan et al. 2020) (Please see lines 201-203) to reduce the duplications caused by heterozygosity in both Nanopore and Hifi read based assemblies (please see Figure S1). According to Pseudohaploid manual and Guan et al. (2020), removing heterozygous duplications should not only improve scaffolding but also gene annotation. However, because such duplication could not be completely removed (please see Figure 1 in Guan et al. 2020 and Purge_Dups website discussion issue https://github.com/dfguan/purge_dups/issues/74), we therefore run the deduplication multiple times (please see the "Mbe_genome_assembly_srcipt.txt" file uploaded to https://doi.org/10.6084/m9.figshare.19165280.v13), which is also supported in the Purge_Dups website discussion issue (https://github.com/dfguan/purge_dups/issues/66). We now add words to make the deduplication step clear, please see line 203.

Reference:
Guan, DF, McCarthy SA, Wood J, Howe K, Wang YD. Identifying and removing haplotypic duplication in primary genome assemblies. Bioinformatics. 2020,36:2896-2898.

b) Combining ONT and HiFi assembled contigs does not make sense to me. Most regions must be present in both assembly. The motivation for this approach could be explained. Was it considered to run a combined assembly with both read sets?

>>>We agree with the reviewer. In the process, we used program RagTag (https://github.com/malonge/RagTag) to correct the Nanopore assembly using HiFi assembly (please see step 17 in "Mbe_genome_assembly_srcipt.txt" uploaded to https://doi.org/10.6084/m9.figshare.19165280.v13). The command is "ragtag.py correct …". The "correct" module in RagTag program only breaks the query sequence at points of putative misassembly but not merge/combine two assemblies (please see https://github.com/malonge/RagTag/wiki/correct).

X2) While it is possible to understand the manuscript, an improvement of the writing could help. This is not about removing typos or grammatical errors, but to improve the

	readability.<br>>>>We have now improved the writing by English language editing company TopEdit (www.topeditsci.com). All revised parts are marked in red.<br><br>Minor comment:<br>line453: compete > complete<br>>>>We have corrected that. Please see line 533.<br><br>--<br>Please also take a moment to check our website at for any additional comments that were saved as attachments. Please note that as GigaScience has a policy of open peer review, you will be able to see the names of the reviewers.<br>>>>We find no attached file(s) that contain additional comments.<br><br>Miscellaneous:<br>Because there are new references added, the order of references in the revised manuscript are changed.<br><br>Please let us know if there are any additional concerns about our manuscript after we have offered these corrections and responses, as we are happy to address any continued issues or proposed changes to the manuscript. Thank you for your time.<br><br>Sincerely yours,<br><br>Xue-Jun Ge<br>South China Botanical Garden<br>Chinese Academy of Sciences<br>Guangzhou, 510650<br>China
**Additional Information:**	
**Question**	**Response**
Are you submitting this manuscript to a special series or article collection?	No
**Experimental design and statistics**<br><br>Full details of the experimental design and statistical methods used should be given in the Methods section, as detailed in our Minimum Standards Reporting Checklist. Information essential to interpreting the data presented should be made available in the figure legends.<br><br>Have you included all the information requested in your manuscript?	Yes
**Resources**<br><br>A description of all resources used, including antibodies, cell lines, animals and software tools, with enough information to allow them to be uniquely	Yes

identified, should be included in the Methods section. Authors are strongly encouraged to cite Research Resource Identifiers (RRIDs) for antibodies, model organisms and tools, where possible.<br><br>Have you included the information requested as detailed in our Minimum Standards Reporting Checklist?	
**Availability of data and materials**<br><br>All datasets and code on which the conclusions of the paper rely must be either included in your submission or deposited in publicly available repositories (where available and ethically appropriate), referencing such data using a unique identifier in the references and in the “Availability of Data and Materials” section of your manuscript.<br><br>Have you have met the above requirement as detailed in our Minimum Standards Reporting Checklist?	Yes

Powered by Editorial Manager® and ProduXion Manager® from Aries Systems Corporation

Manuscript

Click here to access/download;Manuscript;Musa genome-Jan 15, 2023-revised - EditedSV.docx

Genome assembly of *Musa beccarii* shows extensive chromosomal rearrangements and genome expansion during evolution of Musaceae genomes

Zheng-Feng Wang[1,2,3], Mathieu Rouard[4], Gaetan Droc[5,6], Pat (J.S.) Heslop-Harrison[1,7,8], Xue-Jun Ge[1,7*]

[1] Guangdong Provincial Key Laboratory of Applied Botany, South China Botanical Garden, Chinese Academy of Sciences, Guangzhou, China

[2] Southern Marine Science and Engineering Guangdong Laboratory (Guangzhou), Guangzhou, China

[3] Key Laboratory of Vegetation Restoration and Management of Degraded Ecosystems, Key Laboratory of Carbon Sequestration in Terrestrial Ecosystem, South China Botanical Garden, Chinese Academy of Sciences, Guangzhou, China

[4] Bioversity International, Parc Scientifique Agropolis II, 34397 Montpellier, France

[5] CIRAD, UMR AGAP Institut, F-34398 Montpellier, France

[6] UMR AGAP Institut, Univ Montpellier, CIRAD, INRAE, Institut Agro, Montpellier, France

[7] Key Laboratory of Plant Resources Conservation and Sustainable Utilization, South China Botanical Garden, Chinese Academy of Sciences, Guangzhou, China.

[8] Department of Genetics and Genome Biology, University of Leicester, Leicester LE1 7RH, UK

*Address for correspondence:
Xue-Jun Ge, E-mail: xjge@scbg.ac.cn

Zheng-Feng Wang [0000-0002-7599-7891];

Mathieu Rouard [0000-0003-0284-1885];

Gaetan Droc [0000-0003-1849-1269];
Pat (J.S.) Heslop-Harrison [0000-0002-3105-2167];
Xue-Jun Ge [0000-0002-5008-9475]

Abstract

Background: *Musa beccarii* (Musaceae) is a banana species native to Borneo, sometimes grown as an ornamental plant. The basic chromosome number of *Musa* species is x=7, 10, or 11; however, *M. beccarii* has a basic chromosome number of x=9 (2n=2x=18), which is the same basic chromosome number of species in the sister genera *Ensete* and *Musella*. *M. beccarii* is in the section *Callimusa*, which is sister to the section *Musa*. We generated a high-quality chromosome-scale genome assembly of *M. beccarii* to better understand the evolution and diversity of genomes within the family Musaceae

Findings: The *M. beccarii* genome was assembled by long-read and Hi-C sequencing, and genes were annotated using both long Iso-Seq and short RNA-seq reads. The size of *M. beccarii* was the largest among all known Musaceae assemblies (~570 Mbp) due to the expansion of transposable elements and increased 45S rDNA sites. By synteny analysis, we detected extensive genome-wide chromosome fusions and fissions between *M. beccarii* and the other *Musa* and *Ensete* species, far beyond those expected from differences in chromosome number. Within Musaceae, *M. beccarii* showed a reduced number of terpenoid synthase genes, which are related to chemical defense, and enrichment in lipid metabolism genes linked to the physical defense of the cell wall. Furthermore, type III polyketide synthase (T3PKS) was the most abundant biosynthetic gene cluster (BGCs) in *M. beccarii*. BGCs were not conserved in Musaceae genomes.

Conclusions: The genome assembly of *M. beccarii* is the first chromosome-scale genome assembly in the *Callimusa* section in *Musa*, which provides an important genetic resource that aids our understanding of the evolution of Musaceae genomes and enhances our knowledge of the pangenome.

Keywords: ancestral genome reconstruction, biosynthetic gene cluster, comparative genome, gene family, Musaceae, transcription factors, whole genome duplication

Introduction

Bananas are one of the most well-known and highly consumed fruits in the world. Phylogenetic studies of the genus *Musa* (family Musaceae) have shown that the genus comprises two sections: sect. *Musa* and sect. *Callimusa* [1-3]. The basic number of chromosomes in sect. *Musa* (c. 33-50 species) is x=11 (wild accessions are 2n=2x=22) while the basic number of chromosomes in members of sect. *Callimusa* can be x=7, x=9, and x=10 [1, 4]. The approximately 38 species in sect. *Callimusa* is 2n=2x=20, and lower numbers are found in *M. ingens* (2n=2x=14) and *Musa beccarii* (2n=2x=18) [5]. Although the basic number of chromosomes of *M. beccarii* is unique among members within the genus *Musa*, x=9 is shared among species in the two sister genera in the family: *Ensete* and *Musella* [5]. *Musa beccarii* is closely related to *M. maclayi* and *M. peekelii*, and these three taxa form a sub-clade sister to another sub-clade including *M. gracilis*, while *M. ingens* is sister to these two sub-clades.

Musa beccarii (NCBI:txid574481) is endemic in Borneo [5, 6]. Its leaves are long, narrow, bright green, and pest-free, and the vertical inflorescence has large, bright red bracts (Fig. 1). *Musa beccarii* begins flowering after 6 to 8 months, and the height of the plants ranges from 1 to 3 m. It is more compact than most other members of the family Musaceae, and it can be grown indoors as an ornamental plant [7]. The long-lasting bright red and attractive flowers [8] can be used as cut flowers. The conservation status of *M. beccarii* is currently “least concern” [9]; however, some have considered this species to be endangered because of habitat loss [5, 6] and its small, isolated wild populations in the wild. It can be propagated by suckers; tissue culture has also been used in *M. beccarii* [7, 8], and this has aided its conservation.

There are currently 12 fully assembled and annotated Musaceae genomes, including *E. glaucum*, *M. acuminata*, *M. balbisiana*, *M. itinerans*, and *M. schizocarpa,* according to "The Banana Genome Hub" [10]. Only half of these genomes have been assembled at the chromosome scale. The genome of *M. acuminata* was the first to be assembled with its ‘DH Pahang’ genome sequence [11], and its genome was updated in 2021 [12]. The assembly revealed that three rounds of ancient whole genome duplications (WGDs) have occurred in *Musa*. Following WGD, many genes involved in transcription regulation, signal transduction, and translational elongation were retained.

Comparisons of published genomes have revealed that genes associated with transcription factors (TFs), defense-related proteins, enzymes involved in cell wall biosynthesis, and enzymes involved in secondary metabolism are *Musa* lineage-specific. Subsequently, the genome of *M. balbisiana* was assembled [13], and it was updated using a double haploid [14]. Most edible banana cultivars are triploids derived from *M. acuminata* and *M. balbisiana* ancestors. Compared to *M. acuminata*, *M. balbisiana* shows more genome fractionation (gene loss) but contains more biotic and abiotic stress resistance properties [14]. *Musa itinerans* was the third species with its genome assembled [15]. *M. itinerans* is a wild banana native to south-east Asia and one of the cold- and disease-resistance *Musa* species [15]. *Musa schizocarpa* was the fourth species in the genus *Musa* to have its genome assembled [16]. It is native to Papua New Guinea, and a small proportion of its genome has introgressed into many cultivated edible bananas [17]. However, this genome is a draft assembly, and no in-depth comparative genomics was conducted to data. Recently, a draft genome assembly of *Musa textilis*, an important fiber plant, was published [18]. However, this genome assembly is not appropriate for accurate comparative genomic analyses because it is fragmented and incomplete (e.g., only 78.2% of complete Benchmarking Universal Single-Copy Orthologs (BUSCO) genes were retrieved).

All previously assembled *Musa* genomes were from members of the section *Musa*; no genome assemblies have been generated from members of the sect. *Callimusa*. In the *Ensete* sister group, the first chromosome-scale genome assembly of *E. glaucum* (x=9) was recently published by Wang et al. [19]. This genome assembly provided insights into the chromosome rearrangements and fusions that occurred between sister genera. Given that it has the same number of chromosomes as members of the genus *Ensete*, *M. beccarii* might have the most conserved genome structure with respect to the common ancestor between *Musa* and *Ensete* [5]; thus, a genome assembly of *M. beccarii* would provide an excellent resource for studies of genome evolution in the family Musaceae and enhance our knowledge of the pangenome and structural variants in the *Callimusa* section.

Materials and Methods

Sample collection and sequencing

One *M. beccarii* N.W.Simmonds individual planted in a greenhouse in the South China Botanical Garden, Guangdong Province, China, was used for genome sequencing. The orientation of the greenhouse was from north to south. A fan was installed on the southern wall to lower the temperature in summer. The indoor temperature was maintained between 10 and 35°C. The individual was a seedling approximately 50 cm in height and was cultivated in a plastic pot (diameter: 37cm; height: 30cm). The soil in the pot was a 1:1 mixture of Jiffy's TPS fine peat substrate (made from Estonian peat moss, pH 5.8) and sands; no fertilization was applied. The plant was automatically irrigated two times daily at 09:00 and 15:00. A sunshade net was placed on the roof of the greenhouse during the growing period. No other special treatments were applied. The plant was collected for genome sequencing between 14:00 and 15:00 on September 21, 2020.

Briefly, genomic DNA was extracted from fresh leaves using the cetyl trimethylammonium bromide method. Quality control was carried out using a NanoDrop 2000 microspectrophotometer (Thermo Fisher Scientific, USA), Qubit fluorometers (Thermo Fisher Scientific, USA), and gel electrophoresis. High-quality DNA was used to build one short-read (Illumina) and two long-read (Nanopore and PacBio HiFi) whole genome sequencing (WGS) libraries. To perform Hi-C scaffolding, the genomic DNA was cross-linked with formaldehyde and extracted for Hi-C library preparation. Additionally, total RNA from *M. beccarii* leaves of the same individual was extracted, and reverse-transcribed to cDNA for the construction of PacBio full-length cDNA sequencing (Iso-Seq) and short-read cDNA fragment sequencing libraries; both libraries were used for genome annotation. HiFi WGS library construction and sequencing were conducted by Annoroad Gene Technology (AGT, China), and the rest of the sequencing was conducted by GrandOmics Biosciences (GB, China).

The DNA sample was used to prepare a whole-genome shotgun paired-end (2 × 150 bp) Illumina library using the Truseq Nano DNA HT Sample Preparation Kit (Illumina, USA). The library was sequenced on the Illumina HiSeq X Ten platform (RRID:SCR_016385). For Nanopore WGS library construction and sequencing, the DNA fragments were size-selected using the BluePippin system (Sage Science, USA). A sequencing library was prepared using size-selected fragments with the SQK-LSK109 Ligation Sequencing Kit (Oxford Nanopore Technologies, UK), and sequencing was conducted using the Nanopore PromethION sequencer. The Hi-C library was

prepared following the procedure described in a previous study [20] with some modifications. Briefly, fresh *M. beccarii* leaves were cut into 2 cm pieces and immersed in a nuclei isolation buffer with 2% formaldehyde for fixation. Vacuum infiltration was conducted for 20 min in this step. Glycine was added to stop fixation, and vacuum infiltration was conducted for another 15 min. Fixed tissue was rinsed in chilled water, dried on paper, and frozen in liquid nitrogen until nuclei were isolated. The isolated nuclei were digested with 100 units of DpnII (New England Biolabs, USA), and then biotin was marked with biotin-14-dCTP. Extra Biotin-14-dCTP was removed, and ligation was conducted using T4 DNA polymerase (New England Biolabs, USA). The ligated DNA was sheared into 300-600 bp fragments, blunt-end repaired, A-tailed, and purified using biotin-streptavidin-mediated pulldown. Finally, the Hi-C libraries were paired-end sequenced (2 × 150 bp) using the Illumina HiSeq X Ten or MGI DNBSEQ-T7 (RRID:SCR_017981) (MGI Tech, China) sequencing platforms. For HiFi WGS library construction and sequencing, a total of 50 μg of extracted genomic DNA was sheared to approximately 10 kb using Covaris g-Tubes (Covaris, USA). The sheared DNA was purified and concentrated using AMPure PB magnetic beads (Cultek, Spain). HiFi sequencing libraries were then prepared using Pacific Biosciences SMRTbell Template Prep Kit 1.0 (Pacific Bioscience, USA). The constructed library was further size-selected electrophoretically using SageELF systems from SAGE Science, USA. Primer annealing was then performed using the constructed library, and SMRTbell templates were bound to polymerases using the Sequel Binding Kit. Finally, the Pacific Bioscience Sequel II platform (RRID:SCR_017990) was used for sequencing.

For RNA-seq library construction and sequencing, total RNA was extracted from *M. beccarii* leaves using the TRNzol Universal RNA Extraction Kit (Tiangen, China). A NanoDrop™ One UV-Vis spectrophotometer (Thermo Fisher Scientific, USA), Qubit Fluorometer (Thermo Fisher Scientific, USA), and Agilent 2100 Bioanalyzer (Agilent Technologies, USA) were used to evaluate the quality and integrity of RNA. A TruSeq RNA Library Preparation Kit (Illumina, USA) was then used to generate sequencing libraries following the manufacturer's instructions. The Illumina HiSeq X Ten platform was used with paired-end sequence (2 × 150 bp) cDNA libraries. For PacBio Iso-Seq library construction and sequencing, total RNA was extracted, and the quality of the RNA was assessed using the method described above. RNA was then reverse transcribed into cDNA using the

NEBNext® Single Cell/Low Input cDNA Synthesis & Amplification Module (New England Biolabs, USA) and the Iso-Seq Express Oligo Kit (Pacific Bioscience, USA). The cDNA was then purified using the ProNex Beads; the SMRTbell Express Template Prep Kit 2.0 (Pacific Bioscience, USA) was used to prepare the cDNA library. The Sequel Binding Kit was used to anneal sequencing primers to the SMRTbell templates and promote binding to polymerases. Sequencing was performed on the Pacific Bioscience Sequel II platform.

The libraries, sequencing, platforms, and data are summarized in Supplementary Table S1.

Data pre-processing

After sequencing, Sickle v1.33 (Sickle, RRID:SCR_006800) [21] was used to quality trim both short WGS and Hi-C reads by removing reads with base quality values less than 30 and lengths shorter than 80 bp. RECKONER v1.1 [22] was used to further error-correct short WGS reads. The CCS algorithm v6.0.0 (RRID:SCR_021174) [23] was used to process PacBio HiFi reads and obtain consensus reads. The error-corrected short WGS reads and/or HiFi reads were used to estimate the genome size of *M. beccarii* via KmerGenie v1.7044 [24], GenomeScope 2.0 (RRID:SCR_017014) [25], findGSE [26], GCE v1.0.2 [27], MGSE [28], and Gnodes [29]. Both MGSE and Gnodes provide mapping-based genome size estimations, while the others are *k*-mer-based. PacBio Iso-seq reads were processed using IsoSeq v3.0 [30] to obtain full-length transcripts. Basecalling of raw Nanopore sequencing data (FAST5 format) was conducted using Guppy 3.2.10 (Oxford Nanopore Technologies, UK) with default parameters to convert them to the FASTQ format. Porchop v0.2.4 [31] and HiFiAdapterFilt v1.0.0 [32] were used to remove the adapters in Nanopore and PacBio consensus long reads.

Genome assembly

The initial assembly was optimized using different assemblers. NextDenovo v2.3.1 [33], Flye 2.9.1 (RRID:SCR_017016) [34], and Canu 2.2 (RRID:SCR_015880) [35] were used for Nanopore reads. HiFiasm 0.15.2 (RRID:SCR_021069) [36], Flye 2.9.1, and HiCanu (using HiFi mode in Canu 2.2) [37] were used for PacBio HiFi reads. NextDenovo and HiFiasm were finally used for Nanopore and HiFi reads, respectively, based on assembly continuity (see results). Supplementary

Fig. S1 shows the steps used to assemble the *M. beccarii* genome, and the full scripts (file named "Mbe_genome_assembly_srcipt.txt") are provided on the figshare website [38]. Briefly, NextDenovo was used to assemble the genome with Nanopore long reads. The assembly was then polished using Racon v1.4.21 (RRID:SCR_017642) [39] and Hapo-G v1.0 [40]; Pseudohaploid [41] and Purge_Dups v1.2.5 (RRID:SCR_021173) [42] were used to remove duplications caused by heterozygosity in the assembly. PacBio HiFi reads were used to correct the assembly with Inspector (RRID:SCR_004923) [43] and RagTag v2.0.1 [44]. The corrected assembly was scaffolded with Hi-C reads using Scaffhic 1.1 [45], Juicer pipeline 1.6 (RRID:SCR_017226) [46], and 3d-dna 201008 (RRID:SCR_017227) [47]. Gap-filling was performed using TGS-GapCloser v1.0.1 (RRID:SCR_017633) [48]. BUSCO (RRID:SCR_015008) v5.2.2 [49] with the database embryophyta_odb10.2020-09-10 was used to evaluate the quality of the assembly. The completeness of the assembly was also assessed by aligning the Illumina WGS reads using BWA v0.7.17 (RRID:SCR_010910) [50]; the percentage of mapped reads was determined using the "flagstat" command in SAMtools v1.9 (RRID:SCR_002105) [51]. The quality of the sequence assembly was finally evaluated based on the trimmed and error-corrected Illumina WGS reads in SQUAT v1.0 [52] through read mapping quality analytics using the parameter "-sample-size 10000000". SQUAT uses two alignment algorithms, BWA-MEM and BWA-backtrack, to evaluate the mapping quality and calculate percentages of uniquely mapped, multi-mapped, and unmapped reads. It further classifies uniquely mapped reads into those that are perfect matches, those containing substitutions, those with mismatches at the ends (i.e., clips), and others. Perfectly matched and multi-mapped reads are considered highly mapped, and unmapped reads are considered poorly-mapped [43].

Repeat annotation

EDTA v1.9.9 (RRID:SCR_022063) [53] and RED v2.0 [54] were used to identify repeat sequences in the *M. beccarii* assembly, and the results of both analyses were combined using the "merge" command in BEDtools v2.29.2 (RRID:SCR_006646) [55]. The *M. beccarii* assembly was masked using the "maskfasta" command in BEDtools according to the combined repeat sequences. For comparison, repeat sequences in *E. glaucum*, *M. balbisiana*, *M. itinerans*, *M. schizocarpa*, and *M.*

acuminata were also tested using EDTA.

To identify two possible types of centromeric repeat sequences (i.e., Nanica, a long interspersed element [11], and Egcen, tandemly repeated satellite [19] sequences) in the *M. beccarii* assembly, blastn 2.12.0+ [56] was used to conduct searches with default settings, including "-strand both -task megablast -evalue 10 -use_index false -dust 20 64 1 -soft_masking true -max_target_seqs 500 -off_diagonal_range 0". Both types of sequences were detected in all Musaceae genomes, but Egcen sequences were only detected in the genera *Ensete* and *Musella* but not *Musa* [19]. Nanica sequences were obtained from Banana Genome Hub [57], and Egcen sequences were obtained from Wang et al. [19]. Consensus sequences of the tandemly repeated 5S and 45S rDNA monomers in *M. beccarii* were obtained via assembly of the Illumina raw reads into monomers sampled from the Nanopore reads.

Gene prediction and annotation

LoReAn [58], an automated annotation pipeline designed for eukaryotic genome annotation, was used for structural gene prediction. In addition to *ab initio* gene prediction, both long and short RNA-seq reads and protein sequences from three species, *M. balbisiana*, *M. schizocarpa*, and *M. acuminata* (Supplementary Table S2), were used for RNA-seq and protein evidence-based gene prediction in LoReAn. The results were then input into the Funannotate pipeline v1.8.7 [59] to obtain final integrated and consensus gene sets using the commands "funannotate train" and "funannotate predict" and the parameters "-max_intronlen 100,000 -busco_db embryophyta -organism other". Gene prediction completeness was evaluated by BUSCO using the database embryophyta_odb10.2020-09-10 with the parameter "--mode prot", and only the longest transcripts were used.

After gene prediction, the command "funannotate annotate" was used to functionally annotate genes. The following databases were used to annotate genes: dbCAN v9.0 (RRID:SCR_013208) [60], eggNOG v5.0.2 (RRID:SCR_002456) [61], Gene Ontology (GO, RRID:SCR_002811) [62,63], Kyoto Encylopedia of Genes and Genomes (KEGG, RRID:SCR_012773) [64], InterPro v5.52-86 (RRID:SCR_006695) [65], MEROPS v12.2 (RRID:SCR_007777) [66], Pfam v34.0 (RRID:SCR_004726) [67], and UniProt v2021_03 (RRID:SCR_002380) [68].

Because many isoforms in genes were identified via the Funannotate pipeline when short- and long-read transcripts were used for gene annotation, SUPPA v2.3 [69] was used to investigate alternative splicing (AS) events. AS events were classified into seven types: skipping exon (SE), alternative 3' (A3) splice sites, alternative 5' (A5) splice sites, mutually exclusive exons (MXEs), retained intron (RI), alternative first exons (AFEs), and alternative last exons (ALEs).

For gene function comparison, the protein-coding genes of all other species used in our phylogenetic analysis (see below) were also functionally annotated using the same procedures used for *M. beccarii*. After annotation, only the longest transcript for each gene in all the species was used in subsequent analyses unless mentioned otherwise.

Given the importance of TF genes in the genomes, these genes were identified and compared in Musaceae species using iTAK [70]. In addition, MYB TFs, the largest TF family in plants and Musaceae (see results), were further identified using MYB_ annotator [71].

Gene families and comparative genomics

Gene families in *M. beccarii* and 14 other species (Supplementary Table S3) in monocots were identified using OrthoFinder v2.5.4 (RRID:SCR_017118) [72, 73] through comparison of their protein-coding gene sequences. Following the gene family identification, genes specific to Musaceae *Musa* and *M. beccarii* were extracted to compare predicted gene functions. A total of 1,125 single-copy ortholog sequences from all species were then used to conduct a phylogenomic analysis using RAxML-NG v1.0.3 (RRID:SCR_022066) [74] with the model JTT+I+G4+F, which was determined to be the optimal model according to ModelTest-NG v0.1.7 [75]. Based on the inferred phylogenetic tree, MCMCTree [76] was used to estimate divergence times; nine species pairs were used as calibration points, and their estimated divergence times were obtained from TimeTree (RRID:SCR_021162)(Supplementary Table S4). MCMCTree runs were conducted with the following parameters: burn-in of 2,000,000, sample frequency of 10, and sample number of 4,000,000. Two runs were performed to ensure the convergence of the posterior distribution. Using the dated tree, CAFE v5 (RRID:SCR_018924) [77] was used to identify gene families (i.e., orthologous groups) that had potentially undergone expansions or contractions. When running CAFE, families that were not at the phylogenetic root were filtered.

For the above gene sets (family/genus/species-specific and expanded/contracted), GO and KEGG enrichment analyses were performed using TBtools v1.098669 [78]. In the enrichment analysis, all the predicted genes with their GO/KEGG annotations were used as the background/reference gene set; the query gene set comprised the genes obtained from the above analyses (e.g., expanded/contracted genes). *P*-values were obtained from hypergeometric tests and corrected using the Banjamini-Hochberg (BH) method. Significantly enriched (*P*-value <0.05 following BH adjustment) GO terms (the name of the term, i.e., biological process, among others) were further grouped and visualized using a treemap generated in REVIGO (RRID:SCR_005825) [79].

Whole genome duplication (WGD)

Ancient WGD events in *M. beccarii* and the other five species in Musaceae were detected using wgd v1.2 [80]. Ksrates v1.1.1 [81] was used to characterize the timing of WGD events with respect to speciation events between *M. beccarii* and the other *Musa* species. Ksrates is based on the wgd package, but it rescales the synonymous nucleotide substitution (*Ks*) estimation by considering different *Ks* rates among lineages in a given phylogenetic tree, which permits more accurate inferences of speciation events. A simplified phylogeny obtained from the gene family analysis above was used in the ksrates analysis; only species in the family Musaceae were considered, and *E. glaucum* was used as an outgroup species.

The DupGen_finder pipeline [82] was used to determine the number of duplications derived from WGD and other types of duplication events. Through searches of homologous gene pairs, DupGen_finder also identified possible tandem duplications (TDs), proximal duplications (PDs), transposed duplications (TRDs), and dispersed duplications (DSDs). TDs are defined as duplications in which the duplicated sequence is next to the original sequence (separated by five or fewer genes), PDs are defined as duplications in which the duplicated sequence is ten or fewer genes away from the original sequence, TRDs are defined as transposable element mediated duplications, and DSDs are random non-neighboring duplications. GO and KEGG enrichment analyses were conducted using TBtools v1.098669 for genes derived from each type of duplication event. Significantly enriched GO terms were further grouped and visualized using a treemap generated in

REVIGO if needed.

Whole genome alignment and synteny analysis

Syntenic blocks within the *M. beccarii* assembly and between the Musaceae assemblies were analyzed using MCScan (RRID:SCR_017650, Python version) in the jcvi package and visualized using the jcvi v1.1.19 [83] and Shinycircos [84] packages. The default parameters for the synteny analysis in MCScan were used, with the exception that the parameter "minimum number of anchors" was set to 10. MCScanX (RRID:SCR_022067, match score 3 and match size 10) [85] was also used, and results were visualized using SynVisio [86]. Dot plot alignments between Musaceae genome assemblies were generated and visualized using D-GENIES v1.2.0 (RRID:SCR_018967) [87]

Biosynthetic gene clusters (BGCs)

BGCs in Musaceae species were identified by plantiSMASH v1.0 (Plant Secondary Metabolite Analysis Shell) [88]. The libraries used in PhytoClust [89] were also used when running the plantiSMASH tool to enhance BGC identifications.

Nucleotide-binding site-leucine-rich repeat (NBS-LRR) gene identification

NBS-LRR genes are the major plant resistance genes serving as an active defense against pathogens [90]. There are generally three main types of NBS-LRR genes [91]: Toll/interleukin-1 receptor NBS-LRR (TNL), N-terminal coiled-coil motif NBS-LRR (CNL), and resistance to powdery mildew8 NBS-LRR (RNL) genes; TNL genes are absent in monocots [91, 92]. According to the results of InterPro/Pfam annotation in the tested Musaceae species, NBS-LRR genes were identified using the following protein domains: IPR03800, PF00931/IPR002182, PF13855/PF00560/IPR032675, and PF05659/IPR008808.

NBS-LRR genes were also detected using NLR-Annotator [93] with default settings. Rather than using annotated proteins predicted by gene models and transcriptomic data, NLR-Annotator directly uses genomic sequences to identify possible NLR genes, which were confirmed to be most efficient in NLR gene identification. After NLR genes were detected, NLR-Annotator categorized NLR genes as 'complete', 'complete (pseudogene)', 'partial', or 'partial (pseudogene)' according

to the properties of each gene.

Ancestral genome reconstruction

AnChro [94] was used to reconstruct ancestral genomes of Musaceae members with ginger (*Zingiber officinale*, GenBank accession number of GCA_018446385.1) as an outgroup. SynChro [95] was used to identify conserved syntenic blocks between different pairs of genomes; the blocks in the two genomes (with the shortest path connecting them in the phylogenetic tree) were then used to infer ancestral gene order by comparing them with the reference genomes. During syntenic block inferences, the stringency parameter, which determines the number of reciprocal best hits within a syntenic block, was set to three.

Results

The size of the *M. beccarii* genome was inferred to be 554,284,138 bp by KmerGenie under the best-selected *k*-mer size of 87 following comparisons of different *k*-mer spectra. Using different programs, the estimated genome size of *M. beccarii* ranged from 547,121,747 bp to 746,096,492 bp (Supplementary Table S5); the size of the genome was predicted to be between 565,341,681 bp and 661,920,459 bp according to the two mapping-based genome estimation programs MGSE and Gnodes. The level of heterozygosity in the genome estimated by GenomeScope ranged from 0.287% to 0.815%.

The assembly sizes using different assemblers ranged from 607,623,222 bp (NextDenovo) to 816,255,026 bp (Canu) with Nanopore reads, and 636,694,734 bp (Hifiasm) to 1,247,860,321 bp (HiCanu) with HiFi reads (Supplementary Table S6). The Nexdenovo and Hifiasm assemblers displayed superior contig numbers, average and minimum lengths, and N50 values for Nanopore and HiFi reads, respectively; these two assemblies were used in subsequent steps of the genome assembly process. Details of the Nexdenovo and Hifiasm assembly results are shown in Table 1.

After Hi-C read scaffolding, the assembly size was 569,617,942 bp with 449 scaffolds; the N50 value was 67,088,101 bp; and 551,683,906 bp (96.85%) of the sequences were assembled into nine chromosomes (Table 1, Figure 2A). The largest chromosome (chr2) was 79,885,826 bp, and the shortest chromosome (chr9) was approximately two times smaller (38,409,407 bp) (Table 1).

BUSCO assessment of the final genome sequence assembly yielded a completeness score of 98.4% for the Embryophyta (1,614 core genes) datasets, including 1,510 (93.6%) complete and single-copy genes and 78 (4.8%) complete and duplicated genes. Another 11 genes (0.7%) were reported as fragmented, and 15 (0.9%) were reported as missing. The integrity of the genome sequence assembly was evaluated by mapping the Illumina WGS reads using BWA: 99.83% of the reads were mapped reads and 95.26% of the reads were correctly paired.

The mapping quality metrics generated by SQUAT revealed 81.5% uniquely mapped reads (62.1% perfectly-matched, 14.5% with substitution errors, 2.3% containing clips, and 2.6% with other errors), 18.3% multiply mapped reads, and 0.2% unmapped reads in the BWA-MEM mode; 79% uniquely mapped reads (62.5% perfectly-matched, 14.9% with substitution errors, and 1.6% with other errors), 16.1% multiply mapped reads, and 4.9% unmapped reads were identified in the BWA-backtrack mode. The overall percentage of poorly-mapped reads in the two modes was 2.5%.

Annotation of repeat sequences

Repetitive regions comprised 51.79% (295,005,341 bp), and 51.45% (293,068,842 bp) of the genome assembly was identified according to EDTA and RED software. EDTA indicated that the most abundant repetitive sequences were long terminal repeat (LTR) retrotransposons, which accounted for 43.47% (247,628,340 bp) of the assembly, followed by terminal inverted repeats (TIRs), which accounted for 5.53% (31,478,316 bp) of the assembly (Supplementary Table S7). The largest proportion of LTR elements were Copia-like (144,383,969 bp, 25.35%) and Gypsy-like (51,691,527 bp, 9.07%) sequences.

A total of 318,946,703 bp (55.99%) of the genome sequence was annotated and masked as repetitive components when the results of EDTA and RED software were combined. The density of repeat sequences in the assembly is shown in Fig. 2B. Comparative analysis indicated that *M. beccarii* contained the highest number of repetitive sequences and the longest repetitive sequences (Fig. 3, Supplementary Table S7), which were mainly LTR retrotransposons and a small number of nonTIR, helitrons.

The 'seed' Nanica repetitive sequence from *M. acuminata* is 5,291 bp long. A BLAST search revealed 822 Nanica-like sequences in *M. beccarii* with lengths ranging from 55 to 3,891 bp, and

668 of them were longer than 1,000 bp. However, these sequences were not only concentrated in the centromere (Supplementary Fig. S2A). The ‘seed’ sequence of Egcen repeats from *E. glaucum* was 134 bp; no similar sequences were detected in *M. beccarii* via BLAST searches or short/long WGS read-mapping of the assembly.

Three 45S rDNA repeats (18S, 5.8S, and 26S rRNA genes as well as intergenic spacers) were detected on chr5 (around bp 21,300,000), chr9 (around bp 38,200,000 near the telomere), and chr7 (around bp 44,400,000). The consensus monomer sequence was 10,402 bp long with a GC content of 60%. The consensus sequence included 17 copies of a tandem repeat (MuTR; GenBank AM905874 to AM905898), although the number of MuTR repeats varied between rDNA monomers in the Nanopore long-molecule reads. Excluding MuTR repeats, present at multiple genome sequence sites, the 45S rDNA repeat was present in 5.0% of the examined short sequence reads. The 5S rDNA monomer was 432 bp long with a GC content of 55.8%, and it was present in 0.11% of the reads; the major sites were located on chr8 at bp 16,100,000 and chr7 at 37,424,000. Peaks associated with the higher GC content of rDNA sequences (assembly average 38.7% GC) are shown in the GC content plot (Fig. 2B).

Gene prediction and annotation

A total of 39,112 genes encoding 45,461 proteins were predicted in *M. beccarii*. Of these genes, 38,756 (85.25%) were functionally annotated (Supplementary Table S8) with a completeness score of 94.8% in the embryophyta_odb10 database, according to the BUSCO assessment.

Alternative splicing events were detected in 4,602 genes. Retained introns were the most frequent events (2,847), followed by alternative 3' splice sites events (830). Other detected events include skipping exon events (313), alternative 5' splice sites (424), mutually exclusive exons (8), alternative first exons (109), and alternative last exons (103).

A total of 3,168 genes encoding TF in *M. beccarii* were identified, and this was similar to the number of TF genes identified in the genomes of *M. acuminata* and *E. glaucum* (Figure 2C; Supplementary Table S9). Among these genes, MYB genes were the most abundant in *M. beccarii* and in the other Musaceae species (Supplementary Table S10). Using the MYB_annotator, a total of 292 MYB genes were identified in *M. acuminata*; this is similar to 294 previously reported [96];

268 MYB genes were identified in the *M. beccarii* genome (Supplementary Table S10 and S11). MYB genes associated with "axillary meristem, root growth", "cell wall, lignin, seed oil, axillary meristem", "defense, stress response", "repressor phenylpropanoid, sinapate, lignin" and "stress response, hormone signaling" were more abundant in the Musaceae genomes than in the genomes of other species (Supplementary Table S12). The three anthocyanin genes, MB_008808-T1, MB_018229-T1, MB_003891-T1 in *M. beccarii* were orthologous to *Musa*MYB-α, -β, and –γ in *M. acuminata*, and this is associated with the transcriptional activation of anthocyanin biosynthesis in banana [97].

Orthogroup identification and gene enrichment

A total of 32,123 orthogroups were identified from a set of 495,640 genes from selected monocots species. In the *M. beccarii* genome, 83.90% (32,815/39,112) of the genes were assigned to 50.03% (16,070/32,123) of the gene families, and 248 gene families composed of 671 genes were specific to *M. beccarii* (Supplementary Table S13).

A total of 7,810 gene families, which included 3,531 genes, were specific to Musaceae. GO and KEGG enrichment analysis revealed that these Musaceae-specific genes in *M. beccarii* were involved in the regulation of protein modification, transcription, and cell wall in the GO Biological Process (BP) category (Supplementary Table S14, Supplementary Fig. S3), and flavonoid biosynthesis, tryptophan metabolism, and phenylpropanoid biosynthesis in KEGG (Supplementary Table S15).

The five *Musa* species shared 22,000 gene families; 11,136 of these genes were shared among all five *Musa* species, and these genes were considered to be core families (Supplementary Fig. S4). There were 25,754 *M. beccarii* genes in the core families. The GO analysis revealed that these genes were mainly involved in cellular and metabolic processes in the BP category, and binding and catalytic activity in the Molecular Function (MF) category (Supplementary Fig. S5). Transcription regulator and transporter activities were the other two main functions in the MF category. A total of 1,062 gene families comprising 1,617 genes were specific to the *M. beccarii* genome (Supplementary Fig. S4).

A phylogenetic tree (Figure 2C) revealed that *M. beccarii* had an estimated divergence time

from the other *Musa* species of approximately 25.26 (95% CI: 8.25-54.84) million years ago. A total of 12,211 gene families were used in the gene family expansion and contraction analysis. In the *M. beccarii* genome, 1,518 gene families have expanded, and 885 gene families have contracted; 84 of these expansions were significant ($P < 0.05$) and 50 of these contractions were significant. Significantly expanded gene families were mainly involved in transcription, carbohydrate metabolism, and membrane transport (Supplementary Table S16 and S17, Supplementary Fig. S6). Significantly contracted gene families were mainly involved in defense response according to the GO analysis and (mono)terpenoid biosynthesis and translation factors according to the KEGG analysis (Supplementary Table S18 and S19).

Genes with alternative splicing were mainly involved in mRNA 3'-end processing, amino acid catabolic processes, phosphorus metabolic processes, response to stress (such as DNA repair), and taurine and hypotaurine metabolism according to the GO and KEGG analyses (Supplementary Table S20 and S21, Supplementary Fig. S7)

Gene duplications

All Musaceae species have undergone the same three ancient WGD events (Fig. 4A), and the five *Musa* species diverged following the WGD events (Fig. 4B).

Gene duplication analyses in *M. beccarii* revealed 11,244 gene pairs that possibly derived from WGDs, 531 pairs derived from tandem duplications, 646 pairs derived from proximal duplications, 2,313 pairs derived from transposed duplications, and 7,690 pairs derived from dispersed duplications. The enrichment analysis revealed that duplicated genes derived from WGDs were mainly involved in transcription, signaling, defense, environment adaptation, and root development (Supplementary Table S22 and S23, Supplementary Fig. S8 and S9A). Genes derived from tandem duplications were mainly involved in various metabolic processes related to stress responses (e.g., glutathione and phenylpropanoid metabolism) and defense (e.g., cell wall formation and membrane transport) (Supplementary Table S24 and S25, Supplementary Fig. S10 and S9B). Genes derived from proximal duplications were mainly involved in benzoxazinoid, terpenoid, and flavonoid biosynthesis, membrane transport, and cell wall formation (Supplementary Table S26 and S27, Supplementary Fig. S9C). Genes derived from transposed duplications were mainly involved in ion

transport (Supplementary Table S28). Genes derived from dispersed duplications were mainly involved in DNA repair, monosaccharide metabolic process, and prokaryotic defense system (Supplementary Table S29 and S30, Supplementary Fig. S11 and S9D).

Whole genome alignment and synteny analysis

Overall, syntenic relationships of *M. beccarii* with the other Musaceae assemblies were observed, including several major syntenic blocks of genes with extensive rearrangements, including fusions, fissions, and translocations (Fig. 4C and Fig. 5). Chr4 of *M. beccarii* showed the highest conserved relationship with chr4 of other *Musa* species (Fig. 5). Chr5, which was the only one conserved between *E. glaucum* and *M. acuminata*, was divided into chr3 and chr5 in *M. beccarii* (Supplementary Fig. S12); this indicates that this fission is specific to *M. beccarii*. The other chromosomes of *M. beccarii* have undergone various rearrangements; for example, chr3 is syntenic with five chromosomes of both *E. glaucum* and *M. acuminata* (Supplementary Fig. S12).

Synteny analysis of the ancient WGDs within *M. beccarii* revealed 233 syntenic blocks containing 9,594 genes and 5,512 gene pairs. The size of the longest syntenic block was 8,645,195 bp, and it contained 44 gene pairs between chr5 and chr6; the smallest syntenic block was 161,642 bp, and it contained 15 gene pairs between chr2 and chr3 (Supplementary Table S31). The syntenic relationships are shown in the CIRCOS plot (Fig. 2B).

A total of 196, 111, 155, and 141 syntenic blocks in *M. beccarii* were shared with *Ensete glaucum*, *M. balbisiana*, *M. acuminata*, and *M. schizocarpa*, respectively. The largest blocks occurred in the chr4s of *M. beccarii* and the other *Musa* species (Fig. 4C and Fig. 5). These largest blocks contained 1,776, 2,495, and 1,675 gene pairs between *M. beccarii* and *M. balbisiana*, *M. acuminata* and *M. schizocarpa*, respectively, including a total of 2,602 genes in *M. beccarii* chr4.

Biosynthetic gene clusters (BGCs)

M. beccarii contained 66 possible BGCs (Table 2 and S32), the second largest number of BGCs in sequenced genomes of Musaceae species after *M. acuminata* (72 clusters). The most abundant BGCs in all Musaceae species were similar to type III polyketide synthase (T3PKS) and tomatine clusters. The BGCs in chr4 of *M. beccarii* and their syntenic genes in the chr4s in the other *Musa*

species are shown in Figure 4D; these findings indicate that BGCs are not conserved in *Musa*. Substantial gains and losses of BGC and genes were observed.

Identification of NBS-LRR genes

Using the annotated protein sequences in Musaceae species, the highest number of CNL genes was detected in *M. itinerans* (59 genes), and the lowest number of CNL genes was detected in *M. balbisiana* (14). A total of 31 CNL genes were detected in *M. beccarii* (Fig. 2C). Only one RNL gene was detected in each Musaceae species.

NLR-annotator was used to identify the most complete and highest overall number of NBS-LRR genes in *M. beccarii* (Supplementary Fig. S2B, Supplementary Table S33); the most NBS-LRR genes were observed in chr6, and no NBS-LRR genes were identified in chr5 (Supplementary Fig. S2B). Only 74 NBS-LRR genes were identified in *M. beccarii* when the genes were detected using predicted genes (Supplementary Table S34), much less than the 179 genes obtained from the genome sequence. The number of NBS-LRR genes detected using predicted genes was also low in *M. balbisiana* compared with the number of genes identified from its genome sequence (43/96). In *M. acuminata*, which has the most complete genome sequence, the numbers of NBS-LRR genes identified from predicted genes and the genome sequence were 111 and 128. In *M. itinerans*, the numbers were 149 and 138, most likely stems from the fragmented draft assembly.

Ancestral genome reconstruction

The ancestral genome reconstruction revealed 86 contigs in the genome of the last common ancestor (LCA) and between 19 and 40 contigs in the genome of the intermediate ancestors in Musaceae (Fig. 6). Although these ancestral genomes are fragmented, the complex chromosomal rearrangements that occurred between *Ensete*, *Musa* sect. *Musa*, and *Musa* sect. *Callimusa* are evident in these genomes. The number of macro- and micro-rearrangements in these Musaceae species was high; the number of contig rearrangements ranged from 18 for *M. acuminata* to 91 for *M. beccarii*, which is consistent with the phylogeny.

Discussion

Genome size

The chromosome-scale assembly of *Musa beccarii* identified nine pseudomolecules between 38 and 79 Mbp long (Fig. 2, Table 1), which is more variable than those in other *Musa* species (e.g., between 35 and 51 Mbp in *M. acuminata*,) [12]. *M. beccarii* has the largest genome (assembly size ≈ 570 Mbp, consistent with various estimates from *k*-mers) among species within the family Musaceae [5, 98]. The genome size of *M. beccarii* was estimated to be between 764 Mb and 804 Mb according to DNA flow cytometry [5, 99], which might be an overestimation compared with the *k*-mer/mapping-based genome size estimation. Similar to other *Musa* species, the size of the genome estimated by flow cytometry [98-100] was larger than the genome assembly (534-578 vs. ~457 Mbp in *M. balbisiana*, 591-646 vs. ~469 Mbp in *M. acuminata*, and 704 vs. 515 Mbp in *M. schizocarpa*). Variation in the size of the genome estimated among methods might be affected by many factors, including the accuracy of flow cytometry, reference values used based on chemical measurements [101-103], variation among samples [100, 104], reference genome staining, and errors in genome assembly. Regardless of the method used to estimate the genome size, the data indicate that *M. beccarii* has the largest genome size among *Musa* species [5, 98]. The relationship between genome sizes in Musaceae species and their biological functions requires further study. Furthermore, variation in the structure of the genome between *M. beccarii* and *M. acuminata* indicates that the large genome of *M. beccarii* stemmed from changes in several chromosomes (Supplementary Table S35).

Repetitive sequences, especially TEs, are important elements driving genome expansion [105-107], at least in species with genomes smaller than 5 Gbp [108]. We detected a clear increase in the number and length of TEs in *M. beccarii*, which is approximately 30 Mbp-106 Mbp larger than those of *E. glaucum* and the other three *Musa* species (Supplementary Table S7; Fig. 3). Previous studies using low coverage sequencing have shown that *M. beccarii* contained the highest repetitive sequences among the five tested *Musa* species, including *M. balbisiana* and *M. acuminata* [98]; this suggests that increases in repetitive sequences might contribute to the larger genome size of *M. beccarii*. Nevertheless, for each TE, only LTR-unknown and helitron were consistently more abundant in the *M. beccarii* genome (51,552,844 bp and 15,898,685 bp, respectively) than in all genomes examined (Supplementary Table S7). Using LTR markers, Häkkinen et al. [6] identified

rich and distinct LTR in *M. beccarii*, suggesting diversification of LTR, resulting in unknown LTRs in *M. beccarii*.

The assembly revealed the presence of three pairs of 45S rDNA loci, compared with only one in the other *Musa* and *Ensete* species assemblies, which is consistent with the *in situ* hybridization results of Bartoš et al. [99]. The 45S rDNA on three chromosomes accounted for 5.0% of the Illumina sequence reads in *M. beccarii* compared with 1.2% in *E. glaucum* on one chromosome [19], so increased rDNA copy number is responsible for some of the increase in genome sequence size.

Gene family evolution

Gene family expansion due to duplications in *Musa*, including *M. beccarii*, was mainly caused by ancient WGD events. TFs are abundant in Musaceae species and higher than all the other monocots used in our dataset, with the exception of *Zingiber officinale* (Fig. 2C, Supplementary Table S9), which is a tetraploid species. The enrichment analysis of *M. beccarii* revealed that genes encoding TFs are some of the main genes retained following WGD events (Supplementary Table S23; Supplementary Fig. S8 and S9A). The fact that Musaceae species have experienced the same WGD events indicates that the retained genes encoding TFs play key roles in mediating adaptation to stress by TFs in Musaceae, which is consistent with results of previous studies of the genomes of *M. acuminata* and *M. itinerans* [11, 15]. To identify possible duplications caused by polyploidies or aneuploidies, the mapping results of Illumina WGS reads in the *M. beccarii* assembly were examined, including the mapping coverage distribution along the chromosomes and the allele frequencies of single nucleotide variants according to Busche et al. [109]. The mapping coverages were constant along all chromosomes (Supplementary Figure S13), and a peak allele frequency of 0.5 was observed in all the chromosomes (Supplementary Figure S14), suggesting that no large segmental duplications have occurred in diploid *M. beccarii*.

We detected a contraction of gene families containing genes involved in defense response, monoterpenoid biosynthesis, and terpenoid backbone biosynthesis (Fig. 2C, and *M. beccarii* had fewer genes than other Musaceae species; Supplementary Table S18 and S19). Terpenoids are important natural products [110-112]. They comprise diverse components and have various

applications, particularly for defense [111], acting as toxic compounds against biological stress agents in plants. Aside from terpenoid backbone and monoterpenoid biosynthesis, according to KEGG, there are other terpenoid biosynthesis (TB)-related pathways, such as steroid biosynthesis (ko00100), ubiquinone and other terpenoid-quinone biosynthesis (ko00130), limonene and pinene degradation (ko00903), diterpenoid biosynthesis (ko00904), brassinosteroid biosynthesis (ko00905), carotenoid biosynthesis (ko00906), zeatin biosynthesis (ko00908), and sesquiterpenoid and triterpenoid biosynthesis (ko00909). No gene families in these pathways have undergone contraction. Because typical terpenoid synthase genes are characterized by two conserved domains with Pfam ID PF01397 and PF03936 [112], the comparison of the genes with these domains in Musaceae revealed that the number of primary terpenoid synthase genes in *M. beccarii* is low (Supplementary Table S9, Fig. 2C). Gene families that have undergone significant expansion in *M. beccarii*, which are mostly involved in transcription, carbohydrate metabolism, and membrane transport (Supplementary Table S16, S17, Supplementary Fig. S6), mediate various processes related to plant growth, development, and defenses [113-116]. Therefore, genes in expanded families might help balance the growth, development, and defense in *M. beccarii*. We also examined NBS-LRR genes in *M. beccarii* and other species in the phylogeny (Fig. 2C). These genes in *M. beccarii* were more abundant (179 genes according to sensitive NLR-annotator analysis [93] in the assembly) than in other Musaceae (67 to 138 genes), although they were less abundant than in most species out of Musaceae (Fig 2C, Supplementary Table S9). Therefore, combing Pfam annotation indicated that NBS-LRR genes might not be a priority in disease defense in the family Musaceae.

The cell wall as a defense (lipid metabolism and adenosine triphosphate (ATP)-binding cassette (ABC) transporters)

Although they do not play a direct role in defense, genes encoding proteins that mediate the synthesis of substances such as cutin, suberin, and wax play a critical role in “physical defense” in plants [117-119]. They are lipids [117, 120] formed by fatty acids and glycerol. They comprise the extracellular hydrophobic layer of cell walls in plants, provide mechanical support, and protect plants from desiccation, extreme temperatures, UV, and attack by pathogens and pests [117, 119]. According to the KEGG PATHWAY database [121], lipid metabolism comprises 16 pathways, and

genes involved in 14 of these pathways were identified in our study species (Supplementary Table S9). Comparative analyses revealed that Musaceae species do not contain more genes involved in these pathways than other species, and the numbers of genes involved in fatty acid elongation (ko00062), cutin, suberine, and wax biosynthesis (ko00073), glycerophospholipid metabolism (ko00564), and ether lipid metabolism (ko00565) pathways was only slightly higher in the genome of *M. beccarii* than in the genomes of other Musaceae species (Fig. 2C).

Cell wall lipids need to be exported to the plant surface to be synthesized in epidermal cells. ABC transporters are essentially required [117, 122]. G family ABC transporters are responsible for the secretion of lipids [123]. We identified a large number of genes in the G family of ABC transporters in all Musaceae species (Supplementary Table S36, both InterPro and eggNOG annotations). The number of ABC transporter genes was highest in Musaceae species according to the eggNOG annotation; however, this was not observed when other annotation pipelines were used; *M. beccarii* had the highest number of ABC transporter genes according to the InterPro annotation. ABC transporters are one of the largest protein families in nature [124]. They bind and hydrolyze ATP and mediate cellular transport processes [124, 125]. They transport molecules such as ions, amino acids, sugars, lipids, peptides, proteins, and antibiotics. The expansion of ABC transporters has been shown to be associated with increases in defenses against abiotic and biotic stress in plants, which promotes adaptation [124, 126, 127].

Flavonoid biosynthesis as a defense

Flavonoids are a ubiquitous group of polyphenolic compounds in plants. They are important secondary metabolites that have been studied extensively from their biosynthesis to their biological activities [128-131], also in Musaceae species [132, 133]. However, no comparative genomic studies of flavonoids have been conducted among Musaceae species. We noticed significant gene enrichment in flavonoid biosynthesis in *M. beccarii* (Supplementary Table S15). Genes involved in flavonoid biosynthesis are high in Musaceae species, and the genome of *M. beccarii* contained the second highest number of flavonoid biosynthesis genes (Supplementary Table S9, Fig. 2C). The high number of flavonoid biosynthesis genes in Musaceae species are majorly derived from the genes encoding naringenin 7-O-methyltransferase (NOMT, KEGG Orthology term K22440).

NOMT can catalyze the methylation of naringenin to produce sakuranetin (Supplementary Fig. S15), a phytoalexin with strong anti-fungal activity [134]. Therefore, the accumulation of NOMT genes involved in flavonoid biosynthesis suggests that flavonoids are functionally important in the resistance to disease in Musaceae and are potentially valuable components of harvested *Musa* crop [135]. ABC transporters are key mediators of flavonoid transport in plants [136, 137]. Both flavonoid biosynthesis and ABC transporters-related genes were enriched in tandem and proximal duplications in *M. beccarii* (Supplementary Table S25 and S27). These two duplicates promote the evolution of self-defense in plants [82]. Future studies are needed to evaluate the presence/absence of flavonoid biosynthesis-related genes among Musaceae and other species, as well as the expression patterns and the biological/phenotypic effects of these genes, as such studies will enhance our understanding of defense mechanisms and other metabolic processes in Musaceae.

M. beccarii has bright red flowers (Fig. 1). Anthocyanins are important substances that affect flower colors [128, 138], and their biosynthesis is closely associated with the flavonoid biosynthesis pathway. Anthocyanins have been shown to affect the red peel of *Musa AAA Cavendish* cv. Baxi [139] and the purple peel of *Musa itinerans* [140] fruits. We found that Musaceae species show little variation in the number of genes involved in anthocyanin biosynthesis compared with the other species (Supplementary Table S9). Given that flavonoid/anthocyanin biosynthesis is mostly regulated at the transcriptional level [132, 141, 142], transcriptome comparisons among different tissues and species are needed to clarify the formation of the red color in the flowers of *M. beccarii*.

Biosynthetic gene clusters (BGCs)

We identified diverse BGCs in Musaceae. BGCs are non-randomly ordered genes along chromosomes that may optimize the synthesis of natural products in living organisms [143, 144]. The most developed cluster in Musaceae BGCs comprises T3PKSs (Table 2, Supplementary Table S32). T3PKSs are homodimer ketosynthases widely distributed in plants, fungi, and bacteria [145]. They take part in various important biosynthesis of secondary metabolites related to polyketides, facilitate the production of various natural products [146, 147], and play a role in defense responses and development [147-149]. In *Musa*, T3PKSs can initiate phenylphenalenones biosynthesis; phenylphenalenones are major phytoalexins involved in the defense against multiple pathogens in

Musa [149]. The T3PKS BGCs in *Musa* merit further investigation because of their potential for enhancing defense systems.

Tomatine alike clusters were also abundant BGCs in *Musa* species. Tomatine is a steroidal glycoalkaloid saponin in tomatoes and other *Solanum* species [150, 151]. It has anti-pathogen and anti-herbivore properties and serves as a natural defense in plants [151-153]. The primary genes involved in tomatine biosynthesis are glycosyltransferases (GTs) [154, 155]. GTs mediate the glycosylation of tomatidine, which is phytotoxic and a steroidal alkaloid (SA), to promote tomatine formation, and reduces the toxicity of SA metabolites to the plant cell [155-157]. Although a tomatine alike steroidal saponin that has been shown to promote resistance to black Sigatoka has been reported in *M. acuminata* [157], the BGC containing the genes that mediate its synthesis remains poorly resolved [158]. The BGCs characterized in the current study provide valuable resources for future studies of the biosynthesis of this saponin in *Musa*.

GTs are ubiquitous enzymes that are involved in the synthesis of various secondary metabolites in plants [159]. They generally function by glycosylating substrates with sugar moieties attached to aglycones, which then form glycosidic bonds. Their acceptor substrates can be sugars, lipids, proteins, nucleic acids, antibiotics, or small molecules [160, 161]. Glycosylation is rendered highly diverse by using various sugar moieties, and it can play diverse roles in plant growth, development, and defense responses [162]. In *M. beccarii*, we detected highly abundant GT-related genes (Supplementary Table S37, Fig. 2C), which might underlie its ability to adapt to environmental conditions. Furthermore, GT family 61 (GT61) genes encode proteins involved in xylan biosynthesis for the cell wall [163, 164]. Xylans are hemicelluloses that can affect cell wall recalcitrance and aid defense against herbivores and pathogens [165]. The number of GT61 genes was highest in *M. beccarii* among Musaceae species (Supplementary Table S37, Fig. 2C). DupGen_finder revealed that 32 (66.7%) of these genes were derived from ancient WGDs; this finding is consistent with the results of a previous study showing that the major duplication of GT families stemmed from WGD events [164, 166]. Experimental studies are needed to fully characterize BGCs.

Chromosomal rearrangements

We detected substantial numbers of chromosomal reorganization events involving chromosomal fusion and fission in *M. beccarii* and the other three *Musa* species (Figures 4C and 6), and only one chromosome remained largely intact. The extensive fusion/fission events between *M. beccarii* in the *Musa* section *Callimusa* and the other *Musa* section *Musa* species are similar in number to those detected between *Musa* and *Ensete* (Figures 4C, 5 and 6 and S12), and are not only a consequence of the reduced number of chromosomes (x=9 vs. x=11). This finding strongly supports the division of *M. beccarii* and the other studied *Musa* species into two different sections. Chromosomal fusion and fission are important mechanisms of speciation [167-169]. However, our current ancestral reconstructions did not permit the numbers of ancestral chromosomes between *Musa* sections or between *Ensete* species to be inferred.

The high abundance and expansion of TEs might facilitate evolutionary genome rearrangements in *M. beccarii* and be responsible for the large structural differences between *M. beccarii* and other studied *Musa* species. Structural rearrangements mediated by various families of TE elements have been reported in other plants [170, 171]; chromosome-scale assemblies anchored by long-molecule sequencing will enable further study of the association of TEs to chromosomal rearrangements.

Our findings indicated that Egcen centromeric tandemly repeated sequences of *Enset*e are absent in *Musa* [19]. Because these repeats were also detected in *Musella*, which is the third genus in the family Musaceae, one possible reason for the absence of Egcen repeats in *Musa* might be the loss of segments stemming from ancestral centromere breakage; this is plausible given that centromeres are hotspots of chromosome rearrangements [172, 173].

AS related to DNA repair systems

We detected AS in at least 11.7% of all the genes in *M. beccarii*. However, this is underestimated because we did not examine the transcriptomes of different tissues and different developmental stages. The enrichment analysis of these genes with AS revealed that they were involved in important cellular responses and DNA repair systems, including DNA repair, nucleotide excision repair, and replication and repair (Supplementary Table S20 and S21). A wide variety of stress conditions can induce DNA damage. DNA repair systems are therefore important for maintaining

the stability of chromosomes in eukaryotic cells [174-176]. AS is a post-transcriptional mechanism that produces many functional proteins from a limited number of genes. AS in *M. beccarii* plays a key role in DNA repair pathways and other processes that mediate evolutionary adaptation.

Conclusion

The assembly of a genome from a member of the section *Callimusa* in the genus *Musa* is important for the development of a pangenome model of Musaceae. The new data reveals extensive rearrangements and expansions, and provide new insights into the range of structural chromosome variation within the family Musaceae. The genes and TFs identified and our structural analysis of the genome are important for conserving biodiversity within the genus *Musa*. Our findings also have implications for breeding novel variants and addressing some of the major challenges faced by banana crop production.

Data availability

We deposited the sequenced reads to NCBI Sequence Read Archive under the accession number SRR16526886 for the Nanopore, and SRR16526885 for PacBio HiFi reads, SRR16526887 for the Illumina WGS reads, SRR16588090 and SRR16588091 for the Illumina Hi-C reads, SRR16351760 for the Illumina RNA-seq reads, SRR16351759 for the PacBio Iso-seq reads. The high-quality genome sequence was submitted to GenBank under the accession number JAIWVJ000000000. Genome Assembly, gene annotation data, and transcriptomic data are also available on the Banana Genome Hub [177] for download or exploration via a dedicated Genome Browser (Jbrowse) and syntenic browser (SynVisio). All supporting data are available in figshare [38] and the *GigaScience* GigaDB database [178].

Ethics Approval and Consent to Participate

No ethical approval/permission is required to obtain the materials and perform the research in this study. Plant material was collected before 1993 and was received at the South China Botanical Garden under an agreement from a European botanical garden.

Competing Interests

The authors declare that they have no competing interests.

Fundings

This work was financially supported by the National Natural Science Foundation of China (No. 32070237, 31261140366), and the Strategic Priority Research Program of Chinese Academy of Sciences (Grant No. XDB31000000).

Authors' contributions

XJG and ZFW designed this experiment. ZFW, MR, GD, and PHH conducted genetic work and data analyses. ZFW, XJG, MR, and PHH drafted the manuscript. ZFW, MR, GD, PHH, and XJG revised the manuscript. All authors gave final approval of the paper.

References

1. Li L-F, Häkkinen M, Yuan Y-M, Hao G, Ge X-J. Molecular phylogeny and systematics of the banana family (Musaceae) inferred from multiple nuclear and chloroplast DNA fragments, with a special reference to the genus *Musa*. *Mol Phylogenet Evol.* 2010;57(1):1-10.
2. Häkkinen M. Reappraisal of sectional taxonomy in *Musa* (Musaceae). *Taxon.* 2013,62: 809-813.
3. Fu N, Ji M, Rouard M, Yan HF, Ge XJ. Comparative plastome analysis of Musaceae and new insights into phylogenetic relationships. *BMC Genom*. 2022,23(1):223
4. Christelová P, Valárik M, Hřibová E, De Langhe E, Doležel J. A multi gene sequence-based phylogeny of the Musaceae (banana) family. *BMC Evol Biol*. 2011,11:103.

5. Häkkinen M, Suchuánková P, Doleželová M, Hřibová E, Doležel J. Karyological observation in *Musa beccarii* var. *hottana* (Musaceae). *Acta Phytotax Geobot.* 2007,58(2/3):112-118.
6. Häkkinen M, Teo CH, Othman YR. Genome constitution for *Musa beccarii* (Musaceae) varieties. *Acta Phytotaxon Sin.* 2007,45(1):69-74.
7. Natarajan N, Sundararajan S, Ramalingam S, Chellakan, PS. Efficient and rapid in-vitro plantlet regeneration via somatic embryogenesis in ornamental bananas (*Musa* spp.). *Biologia*. 2020,75:317-326.
8. Rashid K, Nezhadahmadi A, Othman RY, Ismail NA, Azhar S, Efzueni S. Micropropagation of ornamental plant *Musa beccarii* through tissue culture technique using suckers and male buds as explants. *Life Sci J*. 2012,9(4):2046-2053.
9. Allen R. *Musa beccarii*. The IUCN Red List of Threatened Species 2019: e.T121033043A121033225. 2019. https://dx.doi.org/10.2305/IUCN.UK.2019-3.RLTS.T121033043A121033225.en. Accessed 13 July 2022.
10. Droc G, Martin G, Guignon V, Summo M, Sempéré G, Durant E, et al. The banana genome hub: a community database for genomics in the Musaceae. *Hortic Res.* 2022:uhac221.
11. D'Hont A, Denoeud F, Aury JM, Baurens FC, Carreel F, Garsmeur O, et al. The banana (*Musa acuminata*) genome and the evolution of monocotyledonous plants. *Nature.* 2012,488(7410): 213-217.
12. Belser C, Baurens FC, Noel B, Martin G, Cruaud C, Istace B, et al. Telomere-to-telomere gapless chromosomes of banana using nanopore sequencing. *Commun Biol.* 2021,4(1):1047.
13. Davey MW, Gudimella R, Harikrishna JA, Sin LW, Khalid N, Keulemans J. (2013). "A draft *Musa balbisiana* genome sequence for molecular genetics in polyploid, inter- and intra-specific *Musa* hybrids". *BMC Genomics*. 2013,14:683.
14. Wang Z, Miao H, Liu J, Xu B, Yao X, Xu C, et al. *Musa balbisiana* genome reveals subgenome evolution and functional divergence. *Nat Plants.* 2019,5:810-821.
15. Wu W, Yang YL, He WM, Rouard M, Li WM, Xu M, et al. Whole genome sequencing of a banana wild relative *Musa itinerans* provides insights into lineage-specific diversification of the *Musa* genus. *Sci Rep*. 2016,6:31586.
16. Belser C, Istace B, Denis E, Dubarry M, Baurens FC, Falentin C, et al. Chromosome-scale

assemblies of plant genomes using nanopore long reads and optical maps. *Nat Plants.* 2018,4(11):879-887.

17. Eyland D, Breton C, Sardos J, Kallow S, Panis B, Swennen R, et al. Filling the gaps in gene banks: Collecting, characterizing, and phenotyping wild banana relatives of Papua New Guinea. *Crop Sci.* 2021,61:137-149.
18. Galvez LC, Koh RBL, Barbosa CFC, Asunto JC, Catalla JL, Atienza RG, et al. Sequencing and de novo assembly of Abaca (*Musa textilis* Nee) var. Abuab Genome. *Genes*. 2021,12(8): 1202.
19. Wang Z, Rouard M, Biswas MK, Droc G, Cui D, Roux N, et al. A chromosome-level reference genome of *Ensete glaucum* gives insight into diversity and chromosomal and repetitive sequence evolution in the Musaceae. *Gigascience*. 2022,11:giac027.
20. Belton JM, McCord RP, Gibcus JH, Naumova N, Zhan Y, Dekker J. Hi-C: A comprehensive technique to capture the conformation of genomes. *Methods*. 2012,58(3):268-276.
21. Joshi NA, Fass JN. Sickle: A sliding-window, adaptive, quality-based trimming tool for FastQ files (Version 1.33). 2011. https://github.com/najoshi/sickle. Accessed 3 September 2021.
22. Długosz M, Deorowicz S. RECKONER: read error corrector based on KMC. *Bioinformatics*. 2017,33:1086-1089.
23. CCS (Version 6.0.0). https://github.com/PacificBiosciences/ccs/releases/tag/v6.0.0. Accessed 12 February 2021.
24. Chikhi R, Medvedev P. Informed and automated *k*-mer size selection for genome assembly. *Bioinformatics*. 2014,30:31-37.
25. Vurture GW, Sedlazeck FJ, Nattestad M, Underwood CJ, Fang H, Gurtowski J, et al. GenomeScope: fast reference-free genome profiling from short reads. *Bioinformatics*. 2017,33: 2202-2204.
26. Sun H, Ding J, Piednoël M, Schneeberger K. findGSE: estimating genome size variation within human and Arabidopsis using k-mer frequencies. *Bioinformatics*. 2018,34:550-557.
27. Liu B, Shi Y, Yuan J, Hu X, Zhang H, Li N, et al. Estimation of genomic characteristics by analyzing k-mer frequency in de novo genome project. arXiv. 2013:1308.2012. https://arxiv.org/abs/1308.2012.

28. Pucker B. Mapping-based genome size estimation. BioRxiv. 2019. https://doi.org/10.1101/607390.

29. Gilbert DG. Genes ruler for genomes, Gnodes, measures assembly accuracy in animals and plants. BioRxiv. 2022. https://doi.org/10.1101/2022.05.13.491861.

30. IsoSeq (Version 3.0). https://github.com/ben-lerch/IsoSeq-3.0. Accessed 12 October 2019.

31. Porechop (Version 0.2.4). https://github.com/rrwick/Porechop/releases/tag/v0.2.4. Accessed 8 January 2019.

32. Sim SB, Corpuz RL, Simmonds TJ, Geib SM. HiFiAdapterFilt, a memory efficient read processing pipeline, prevents occurrence of adapter sequence in PacBio HiFi reads and their negative impacts on genome assembly. *BMC Genom*. 2022,23(1):157.

33. NextDenovo (Version 2.3.1). https://github.com/Nextomics/NextDenovo/releases/tag/v2.3.1. Accessed 22 September 2020.

34. Kolmogorov M, Yuan J, Lin Y, Pevzner PA. Assembly of long, error-prone reads using repeat graphs. *Nat. Biotechnol.* 2019,37:540-546.

35. Koren S, Walenz BP, Berlin K, Miller JR, Phillippy AM. Canu: scalable and accurate long-read assembly via adaptive k-mer weighting and repeat separation. *Genome Res.* 2017,27(5):722-736.

36. Cheng H, Concepcion GT, Feng X, Zhang H, Li Heng. Haplotype-resolved de novo assembly using phased assembly graphs with hifiasm. *Nat. Methods*. 2021,18(2):170-175.

37. Nurk S, Walenz BP, Rhiea A, Vollger MR, Logsdon GA, Grothe R, et al. HiCanu: accurate assembly of segmental duplications, satellites, and allelic variants from high-fidelity long reads. *Genome Res.* 2020,30(9):1291-1305.

38. Wang Z-F. Genome assembly of *Musa beccarii*. figshare. Dataset. 2022. https://doi.org/10.6084/m9.figshare.19165280.v13.

39. Vaser R, Sović I, Nagarajan N, Šikić M. Fast and accurate de novo genome assembly from long uncorrected reads. *Genome Res*. 2017,27(5):737-746.

40. Aury JM, Istace B. Hapo-G, haplotype-aware polishing of genome assemblies with accurate reads. *NAR Genom Bioinform.* 2021,3(2):lqab034.

41. Pseudohaploid. https://github.com/schatzlab/pseudohaploid. Accessed 28 August 2020.

42. Guan DF, McCarthy SA, Wood J, Howe K, Wang YD. Identifying and removing haplotypic duplication in primary genome assemblies. *Bioinformatics*. 2020,36:2896-2898.
43. Chen Y, Zhang Y, Wang AY, Gao M, Chong Z. Accurate long-read de novo assembly evaluation with Inspector. *Genome Biol.* 2021,22:312.
44. Alonge M, Lebeigle L, Kirsche M. Jenike K, Ou S, Aganezov S, et al. Automated assembly scaffolding using RagTag elevates a new tomato system for high-throughput genome editing. *Genome Biol.* 2022,23:258.
45. Scaffhic (Version 1.1). https://github.com/wtsi-hpag/scaffHiC. Accessed 28 August 2020.
46. Durand NC, Shamim MS, Machol I, Rao SSP, Huntley MH, Lander ES, et al. Juicer provides a one-click system for analyzing loop-resolution Hi-C experiments. *Cell Syst*. 2016,3(1):95-98.
47. Dudchenko O, Batra SS, Omer AD, Nyquist SK, Hoeger M, Durand NC, et al. De novo assembly of the Aedes aegypti genome using Hi-C yields chromosome-length scaffolds. *Science*. 2017,356(6333):92-95.
48. Xu M, Guo L, Gu S, Wang O, Zhang R, Peters BA, et al. TGS-GapCloser: A fast and accurate gap closer for large genomes with low coverage of error-prone long reads. *Gigascience*. 2020,9(9):giaa094.
49. Seppey M, Manni M, Zdobnov EM. BUSCO: Assessing genome assembly and annotation completeness. *Methods Mol Biol.* 2019,1962:227-245.
50. Li H, Durbin R. Fast and accurate short read alignment with Burrows-Wheeler Transform. *Bioinformatics*. 2009,25:1754-1760.
51. Danecek P, Bonfield JK, Liddle J, Marshall J, Ohan V, Pollard MO, et al. Twelve years of SAMtools and BCFtools. *Gigascience*. 2021,10(2):giab008. doi: 10.1093/gigascience/giab008.
52. Yang LA, Chang YJ, Chen SH, Lin CY, Ho JM. SQUAT: a sequencing quality assessment tool for data quality assessments of genome assemblies, *BMC Genom.* 2019,19:238.
53. Ou S, Su W, Liao Y, Chougule K, Agda JRA, Hellinga AJ, et al. Benchmarking transposable element annotation methods for creation of a streamlined, comprehensive pipeline. *Genome Biol.* 2019,20:275.
54. Girgis HZ. Red: an intelligent, rapid, accurate tool for detecting repeats de-novo on the genomic scale. *BMC Bioinform*. 2015,16(1):227.

55. Quinlan AR, Hall IM. BEDTools: a flexible suite of utilities for comparing genomic features. *Bioinformatics*. 2010,26(6):841-842.

56. Camacho C, Coulouris G, Avagyan V, Ma N, Papadopoulos J, Bealer K, et al. BLAST+: architecture and applications. *BMC Bioinform*. 2009,10:421.

57. Nanica sequences. *Banana Genome Hub*. 2022 https://banana-genome-hub.southgreen.fr/node/50/353. Accessed 15 April 2022.

58. Cook DE, Valle-Inclan JE, Pajoro A, Rovenich H, Thomma BPHJ, Faino L. Long-Read Annotation: Automated eukaryotic genome annotation based on long-read cDNA sequencing. *Plant Physiol*. 2019,179 (1):38-54.

59. Funannotate (Version 1.8.7). https://github.com/nextgenusfs/funannotate/releases/tag/v1.8.7. Accessed 28 April 2021.

60. Zhang H, Tanner Y, Huang L, Entwistle S. dbCAN2: a meta server for automated carbohydrate-active enzyme annotation. *Nucleic Acids Res.* 2018,46:W95-W101.

61. Huerta-Cepas J, Forslund K, Coelho LP, Damian PC, Szklarczyk D, Jensen LJ. Fast genome-wide functional annotation through orthology assignment by eggNOG-mapper. *Mol Biol Evol.* 2017,34:2115-2122.

62. The Gene Ontology Consortium. The gene ontology resource: 20 years and still GOing strong. *Nucleic Acids Res.* 2019,47(D1):D330-D338.

63. Ashburner M, Ball CA, Blake JA, Botstein D, Butler H, Cherry JM. Et al. Gene ontology: tool for the unification of biology. *Nat Genet*. 2000,25:25-29.

64. Kanehisa M, Soto Y, Kawashima M, Furumichi M, Tanabe M. KEGG as a reference resource for gene and protein annotation. *Nucleic Acids Res*. 2016,44(D1): D457-D462.

65. Mitchell AL, Attwood TK, Babbitt PC, Blum M, Bork P, Bridge A., et al. InterPro in 2019: improving coverage, classification and access to protein sequence annotations. *Nucleic Acids Res*. 2019,47(D1):D351-D360.

66. Rawlings ND, Barrett AJ, Thomas PD, Huang XS, Bateman A, Finn RD. The merops database of proteolytic enzymes, their substrates and inhibitors in 2017 and a comparison with peptidases in the PANTHER database. *Nucleic Acids Res*. 2018,46(D1):D624-D632.

67. El-Gebali S, Mistry J, Bateman A, Eddy SR, Luciani A, Potter SC, et al.. The Pfam protein

families database in 2019. *Nucleic Acids Res.* 2019,47(D1):D427-D432.

68. The UniProt Consortium. UniProt: a worldwide hub of protein knowledge. *Nucleic Acids Res.* 2019,47(D1):D506-D515.

69. Trincado JL, Entizne JC, Hysenaj G, Singh B, Skalic M, Elliott DJ, Eyras E. SUPPA2: fast, accurate, and uncertainty-aware differential splicing analysis across multiple conditions. *Genome Biol.* 2018,19:40.

70. Zheng Y, Jiao C, Sun H, Rosli HG, Pombo MA, Zhang P, et al. iTAK: a program for genome-wide prediction and classification of plant transcription factors, transcriptional regulators, and protein kinases. *Mol Plant.* 2016,9:1667-1670.

71. Pucker B. Automatic identification and annotation of MYB gene family members in plants. *BMC genom.* 2022,23:220

72. Emms DM, Kelly S. OrthoFinder: solving fundamental biases in whole genome comparisons dramatically improves orthogroup inference accuracy. *Genome Biol.* 2015,16:157.

73. Emms DM, Kelly S. OrthoFinder: phylogenetic orthology inference for comparative genomics. *Genome Biol*. 2019,20:238.

74. Kozlov AM, Darriba D, Flouri T, Morel B, Stamatakis A. RAxML-NG: a fast, scalable and user-friendly tool for maximum likelihood phylogenetic inference. *Bioinformatics*. 2019,35(21):4453-4455.

75. Darriba D, Posada D, Kozlov AM, Stamatakis A, Morel B, Flouri T. ModelTest-NG: A new and scalable tool for the selection of DNA and protein evolutionary models. *Mol Biol Evol*. 2020,37(1):291-294.

76. dos Reis M, Zhu T, Yang Z. The impact of the rate prior on Bayesian estimation of divergence times with multiple Loci. *System Biol*. 2014,63:555-565

77. Han MV, Thomas GWC, Jose LM, Hahn MW. Estimating gene gain and loss rates in the presence of error in genome assembly and annotation using cafe 3. *Mol Biol Evol.* 2013,30(8):1987-1997.

78. Chen CJ, Chen H, Zhang Y, Thomas HR, Frank MH, He YH, Xia R. TBtools- an integrative toolkit developed for interactive analyses of big biological data. *Mol Plant.* 2020,13(8): 1194-1202.

79. Supek F, Bošnjak M, Škunca N, Šmuc T. REVIGO summarizes and visualizes long lists of gene ontology terms. *PLoS One*. 2011,6(7):e21800.

80. Zwaenepoel A, de Peer YV. wgd-simple command line tools for the analysis of ancient whole-genome duplications. *Bioinformatics*. 2019,35:2153-2155.

81. Sensalari C, Maere S, Lohaus R. *Ksrates*: positioning whole-genome duplications relative to speciation events in K_S distributions. *Bioinformatics*. 2022,38(2):530-532.

82. Qiao X, Li QH, Yin H, Qi K, Li L, Wang R, Zhang S, Paterson AH (2019). Gene duplication and evolution in recurring polyploidization–diploidization cycles in plants. *Genome Biol.* 2019,20:38.

83. JCVI (Version 1.1.19). https://github.com/tanghaibao/jcvi/releases/tag/v1.1.19. Accessed 8 November 2021.

84. Yu Y, Ouyang Y, Yao W. shinyCircos: an R/Shiny application for interactive creation of Circos plot. *Bioinformatics*. 2018,34(7):1229-1231.

85. Wang Y, Tang H, Debarry JD, Tan X, Li J, Wang X, et al. MCScanX: a toolkit for detection and evolutionary analysis of gene synteny and collinearity. *Nucleic Acids Res.* 2012,40(7):e49

86. Bandi V, Gutwin C. Interactive exploration of genomic conservation. In Proceedings of the 46th Graphics Interface Conference on Proceedings of Graphics Interface 2020 (GI'20). Canadian Human-Computer Communications Society, Waterloo, CAN. 2020

87. Cabanettes F, Klopp C. D-GENIES: dot plot large genomes in an interactive, efficient and simple way. *PeerJ*. 2018,6:e4958-e4958.

88. Kautsar SA, Duran HGS, Blin K, Osbourn A, Medema MH. plantiSMASH: automated identification, annotation and expression analysis of plant biosynthetic gene clusters. *Nucleic Acids Res*. 2017,45(W1):W55-W63.

89. Töpfer N, Fuchs LM, Aharoni A. (2017) The PhytoClust tool for metabolic gene clusters discovery in plant genomes. *Nucleic Acids Res.* 2017,45(12):7049-7063

90. McHale L, Tan X, Koehl P, Michelmore RW. Plant NBS-LRR proteins: adaptable guards. *Genome Biol.* 2006,7(4):212.

91. Shao Z-Q, Xue J-Y, Wu P, Zhang Y-M, Wu Y, Hang Y-Y, et al. Large-scale analyses of angiosperm Nucleotide-Binding Site-Leucine-Rich Repeat genes reveal three anciently

diverged classes with distinct evolutionary patterns. *Plant Physiol.* 2016,170(4):2095-2109.

92. Guo X, Fang D, Sahu SK, Yang S, Guang X, Folk, R. et al. Chloranthus genome provides insights into the early diversification of angiosperms. *Nat Commun.* 2021,12(1): 6930.

93. Steuernagel B, Witek K, Krattinger SG, Ramirez-Gonzalez RH, Schoonbeek HJ, Yu G, et al. The NLR-annotator tool enables annotation of the intracellular immune receptor repertoire. *Plant Physiol.* 2020,183:468-482.

94. Vakirlis N, Sarilar V, Drillon G, Fleiss A, Agier N, Meyniel JP, et al. Reconstruction of ancestral chromosome architecture and gene repertoire reveals principles of genome evolution in a model yeast genus. *Genome Res.* 2016,26(7):918-932.

95. Drillon G, Carbone A, Fischer G. SynChro: a fast and easy tool to reconstruct and visualize synteny blocks along eukaryotic chromosomes. *PLoS One.* 2014,9 (3):e92621.

96. Pucker B, Pandey A, Weisshaar B, Stracke R. The R2R3-MYB gene family in banana (*Musa acuminata*): Genome-wide identification, classification and expression patterns. *PLoS One.* 2020,15(10):e0239275.

97. Busche M, Pucker B, Weisshaar B, Stracke R. Three R2R3-MYB transcription factors from banana (*Musa* spp.) activate structural anthocyanin biosynthesis genes as part of an MBW complex. *BioRxiv*. 2022. https://doi.org/10.1101/2022.08.15.503939.

98. Novák P, Hřibová E, Neumann P, Kobližková A, Doleřel J, Macas J. Genome-wide analysis of repeat diversity across the family Musaceae. *PLoS One.* 2014,9(6):e98918.

99. Bartoš J, Alkhimova O, Doleželová M, De Langhe E, Doležel J. Nuclear genome size and genomic distribution of ribosomal DNA in *Musa* and *Ensete* (Musaceae): taxonomic implications. *Cytogenet Genome Res.* 2005,109(1-3):50-57.

100. Lysák MA, Doleželová M, Horry JP, Swennen R, Doležel J. Flow cytometric analysis of nuclear DNA content in *Musa*. *Theo Appl Genet.* 1999,98:1344-1350.

101. Doležel J, Bartoš J. Plant DNA flow cytometry and estimation of nuclear genome size. *Ann Bot*. 2005,95(1):99-110.

102. Pellicer J, Powell RF, Leitch IJ. The application of flow cytometry for estimating genome size, ploidy level endopolyploidy, and reproductive modes in plants. *Methods Mol Biol.* 2021,2222:325-361.

103. Van't Ho, J, Sparrow AH. A relationship between DNA content, nuclear volume, and minimum mitotic cycle time. *P Natl Acad Sci USA.* 1963,49:897-902.

104. Šmarda P, Horová L, Bureš P, Hralová I, Marková M. Stabilizing selection on genome size in a population of *Festuca pallens* under conditions of intensive intraspecific competition. *New Phytol.* 2010,187(4):1195-1204.

105. Piegu B, Guyot R, Picault N, Roulin A, Sanyal A, Kim H, et al. Doubling genome size without polyploidization: dynamics of retrotransposition-driven genomic expansions in *Oryza australiensis*, a wild relative of rice. *Genome Res.* 2006,16(10):1262-1269.

106. Macas J, Novák P, Pellicer J, Čížková J, Koblížková A, Neumann P, et al. In depth characterization of repetitive DNA in 23 plant genomes reveals sources of genome size variation in the Legume Tribe Fabeae. *PLoS One.* 2015,10(11):e0143424.

107. Wicker T, Gundlach H, Spannagl M, Uauy C, Borrill P, Ramírez-González RH, et al. Impact of transposable elements on genome structure and evolution in bread wheat. *Genome Biol.* 2018,19(1):103.

108. Novák P, Guignard MS, Neumann P, Kelly LJ, Mlinarec J, Koblížková A, et al. Repeat-sequence turnover shifts fundamentally in species with large genomes. *Nat Plants.* 2020,6(11): 1325-1329.

109. Busche M, Pucker B, Viehöver P, Weisshaar B, Stracke R. Genome sequencing of *Musa acuminata* Dwarf Cavendish reveals a duplication of a large segment of Chromosome 2. *G3-Genes Genom. Genet.* 2020,10 (1):37-42.

110. Chen F, Tholl D, Bohlmann J, Pichersky E. The family of terpene synthases in plants: a mid-size family of genes for specialized metabolism that is highly diversified throughout the kingdom. *Plant J.* 2011,66(1):212-229.

111. Pichersky E, Raguso RA. Why do plants produce so many terpenoid compounds? *New Phytol.* 2016,220(3):655-658.

112. Jiang SY, Jin JJ, Sarojam R, Ramachandran S. A comprehensive survey on the terpene synthase gene family provides new insight into its evolutionary patterns. *Genome Biol Evol.* 2019,11(8):2078-2098.

113. Rojas CM, Senthil-Kumar M, Tzin V, Mysore KS. Regulation of primary plant metabolism during plant-pathogen interactions and its contribution to plant defense. *Front Plant Sci.* 2014,5:17.

114. Meshi T, Iwabuchi M. Plant transcription factors. *Plant Cell Physiol.* 1995,36(8):1405-1420.

115. Amorim LLB, da Fonseca DSR, Neto JPB, Guida-Santos M, Crovella S, Benko-Iseppon AM. Transcription factors involved in plant resistance to pathogens. *Curr Protein Pept Sci*. 2017,18(4):335-351.

116. Gani U, Vishwakarma RA, Misra P. Membrane transporters: the key drivers of transport of secondary metabolites in plants. *Plant Cell Rep*. 2021,40(1):1-18.

117. Pollard M, Beisson F, Li Y, Ohlrogge JB. Building lipid barriers: biosynthesis of cutin and suberin. *Trends Plant Sci*. 2008,13(5):236-246.

118. Wang A, Zha Z, Yin D, Shu X, Ma L, Wang L, et al. Comparative transcriptome analysis of *Tilletia horrida* infection in resistant and susceptible rice (*Oryza sativa* L.) male sterile lines reveals potential candidate genes and resistance mechanisms. *Genomics*, 2020,112(6): 5214-5226.

119. Ziv C, Zhao Z, Gao YG. Xia Y. Multifunctional roles of plant cuticle during plant-pathogen interactions. *Front Plant Sci*. 2018,9:1088.

120. Baales J, Zeisler-Diehl VV, Schreiber L. Analysis of extracellular cell wall lipids: wax, cutin, and suberin in leaves, roots, fruits, and seeds. *Methods Mol Biol*. 2021,2295:275-293.

121. KEGG PATHWAY Database. https://www.genome.jp/kegg/pathway.html. Accessed 27 January 2022.

122. Pighin JA, Zheng H, Balakshin LJ, Goodman IP, Western TL, Jetter R, et al. Plant cuticular lipid export requires an ABC transporter. *Science*. 2004,306(5696):702-704.

123. Elejalde-Palmett C, Segundo IMS, Garroum I, Charrier L, De Bellis D, Mucciolo A, et al. ABCG transporters export cutin precursors for the formation of the plant cuticle. *Curr Biol.* 2021,31(10):2111-2123.e9

124. Kang J, Park J, Choi H, Burla B, Kretzschmar T, Lee Y, Martinoia E. Plant ABC Transporters. *Arabidopsis Book*, 2011,9:e0153.

125. Bailly, A. Structure-function of plant ABC-Transporters. In: Geisler M editor. Plant ABC

Transporters. Cham: Springer; 2014. p. 219-240.

126. Do THT, Martinoia E, Lee Y. Functions of ABC transporters in plant growth and development. *Curr Opin Plant Biol.* 2018,4:32-38.

127. Banasiak J, Jasiński M. (2022). ATP-binding cassette transporters in nonmodel plants. *New Phytol*. 2022,233:1597-1612.

128. Winkel-Shirley B. Flavonoid biosynthesis. A colorful model for genetics, biochemistry, cell biology, and biotechnology. *Plant Physiol*. 2001,126(2):485-93.

129. Pucker B, Reiher F, Schilbert HM. Automatic identification of players in the flavonoid biosynthesis with application on the biomedicinal plant *Croton tiglium*. *Plants (Basel)*. 2020,9(9):1103.

130. Liu W, Feng Y, Yu S, Fan Z, Li X, Li J, et al. The flavonoid biosynthesis network in plants. *Int J Mol Sci.* 2021,22:12824.

131. Shen N, Wang T, Gan Q, Liu S, Wang Li, Jin B. Plant flavonoids: Classification, distribution, biosynthesis, and antioxidant activity. *Food Chem.* 2022,383:132531.

132. Pandey A, Alok A, Lakhwani D, Singh J, Asif MH, Trivedi PK. Genome-wide expression analysis and metabolite profiling elucidate transcriptional regulation of flavonoid biosynthesis and modulation under abiotic stresses in Banana. *Sci Rep.* 2016,6:31361.

133. Sun X, Gao P, Zhang J, Xu B, Jin Z, Liu J (2018) Characteristics of flavonoids biosynthesis and the differential expression analysis of the key enzyme genes in *Musa* AAA Group cv Brazilian fruit pulp. *Mol Plant Breeding.* 2018,16(7):2116-2123.

134. Murata K, Kitano T, Yoshimoto R, Takata R, Ube N, Ueno K, et al. Natural variation in the expression and catalytic activity of a naringenin 7-O-methyltransferase influences antifungal defenses in diverse rice cultivars. *Plant J*. 2019,101(5):1103-1117.

135. Busche M, Acatay C, Martens S, Weisshaar B, Stracke R. Functional characterisation of Banana (*Musa* spp.) 2-Oxoglutarate-Dependent Dioxygenases involved in flavonoid biosynthesis. *Front Plant Sci.* 2021,12:701780.

136. Petrussa E, Braidot E, Zancani M, Peresson C, Bertolini A, Patui S, Vianello A. Plant flavonoids—biosynthesis, transport and involvement in stress responses. *Int J Mol Sci* 2013,14:14950-14973.

137. Pucker B, Selmar D. Biochemistry and molecular basis of intracellular flavonoid transport in plants. *Plants*. 2022,11:963.

138. Pazmiño-Durán EA, Giusti MM, Wrolstad RE, Glória MBA. Anthocyanins from banana bracts (*Musa* X *paradisiaca*) as potential food colorants. *Food Chem.* 2001,73(3):327-332.

139. Fu X, Cheng S, Liao Y, Huang B, Du B, Zeng W, et al. Comparative analysis of pigments in red and yellow banana fruit. *Food Chem.* 2018,239:1009-1018.

140. Deng S, Cheng C, Liu Z, Chen Y, Zhang Z, Huang Y, et al. Comparative transcriptome analysis reveals a role for anthocyanin biosynthesis genes in the formation of purple peel in Minhou wild banana (*Musa itinerans* Cheesman). *J Hortic Sci Biotechnol.* 2019,94(2):184-200.

141. Jiao F, Zhao L, Wu X, Song Z, Li Y. Metabolome and transcriptome analyses of the molecular mechanisms of flower color mutation in tobacco. *BMC Genom.* 2020,21:611.

142. Zhang X, Lin S, Peng D, Wu Q, Liao X, Xiang K, et al. Integrated multi-omic data and analyses reveal the pathways underlying key ornamental traits in carnation flowers. *Plant Biotechnol. J.* 2022,20(6):1182-1196.

143. Nützmann H-W, Huang A, Osbourn A. (2016). Plant metabolic clusters – from genetics to genomics. *New Phytol.* 2016,211(3):771-789.

144. Polturak G, Osbourn A. The emerging role of biosynthetic gene clusters in plant defense and plant interactions. *PLoS Pathog.* 2021,17(7):e1009698.

145. Yu D, Xu F, Zeng J, Zhan J. Type III polyketide synthases in natural product biosynthesis. *IUBMB Life*, 2012,64(4):285-295.

146. Flores-Sanchez IJ, Verpoorte R. Plant polyketide synthases: A fascinating group of enzymes. *Plant Physiol Biochem.* 2009,47(3):167-174.

147. Mhlanga M. Plant polyketides. *Nat Biotechnol.* 1999,17:9.

148. Rajesh T, Tiwari MK, Thiagarajan S, Nair PS, Jeya M. Type III polyketide synthases: Current state and perspectives. In: Arora P, editor. Microbial Technology for the Welfare of Society. Singapore: Springer; 2019. p. 183-200

149. Pothiraj R, Ravikumar MJ, Suthanthiram B, Subbaraya U, Krishnamurthy P. Genome-scale analyses of polyketide synthases in banana: Phylogenetics and expression profiling forecast their candidacy in specialized metabolism. *Gene.* 2021,778:145472.

150. Gröger D. Terpenoid and steroid alkaloids. In: Constabel F, Vasil IK editors. Phytochemicals in Plant Cell Cultures. Academic Press; 1988. p. 435-448.
151. Piasecka A, Jedrzejczak-Rey N, Bednarek P. Secondary metabolites in plant innate immunity: conserved function of divergent chemicals. *New Phytol.* 2015,206(3):948-964.
152. Hoagland RE. Toxicity of tomatine and tomatidine on weeds, crops and phytopathogens fungi. *Allelopathy J.* 2009,23(2):425-435.
153. Nakayasu M, Akiyama R, Kobayashi M, Lee HJ, Kawasaki T, Watanabe B, et al. Identification of α-Tomatine 23-Hydroxylase involved in the detoxification of a bitter glycoalkaloid. *Plant Cell Physiol.* 2020,61(1):21-28.
154. Itkin M, Rogachev I, Rogachev I, Alkan N, Rosenberg T, Malitsky S, et al. GLYCOALKALOID METABOLISM1 is required for steroidal alkaloid glycosylation and prevention of phytotoxicity in tomato. *Plant Cell.* 2011,23(12):4507-4525.
155. Itkin M, Heinig U, Tzfadia O, Bhide AJ, Shinde B, Cardenas PD, et al. Biosynthesis of antinutritional alkaloids in solanaceous crops is mediated by clustered genes. *Science.* 2013,341(6142):175-179.
156. You Y, van Kan JAL. Bitter and sweet make tomato hard to (b)eat. *New Phytol.* 2020,230(1):90-100.
157. Cruz-Cruz CA, Ramírez-Tec G, García-Sosa K, Escalante-Erosa F, Hill L, Osbourn AE, et al. Phytoanticipins from banana (*Musa acuminata* cv. Grande Naine) plants, with antifungal activity against *Mycosphaerella fijiensis*, the causal agent of black Sigatoka. *Eur J Plant Pathol.* 2010,126(4):459-463.
158. Soares JMS, Rocha AJ, Nascimento FS, Santos AS, Miller RNG, Ferreira CF, et al. Genetic improvement for resistance to Black Sigatoka in Bananas: a systematic review. *Front Plant Sci.* 2021,12:657916.
159. Gachon CM, Langlois-Meurinne M, Saindrenan P. Plant secondary metabolism glycosyltransferases: the emerging functional analysis. *Trends Plant Sci.* 2005,10(11):542-549.
160. Lairson LL, Henrissat B, Davies GJ, Withers SG. Glycosyltransferases: structures, functions, and mechanisms. *Annu Rev Biochem.* 2008,77:521-555.
161. He B, Bai X, Tan Y, Xie W, Feng Y, Yang G-Y. Glycosyltransferases: Mining, engineering and

applications in biosynthesis of glycosylated plant natural products. *Synth Syst Biotechnol.* 2022,7:602-620.

162. Wang J, Hou B-K. Glycosyltransferases: key players involved in the modificationof plant secondary metabolites. *Front Biol China.* 2009,4(1):39-46.

163. Phan JL, Tucker MR, Khor SF, Shirley N, Lahnstein J, Beahan C, et al. Differences in glycosyltransferase family 61 accompany variation in seed coat mucilage composition in *Plantago* spp. *J Exp Bot.* 2016,67(22):6481-6495.

164. Cenci A, Chantret N, Rouard M. Glycosyltransferase family 61 in Liliopsida (Monocot): The story of a gene family expansion. *Front Plant Sci.* 2018,9:1843.

165. Rennie EA, Scheller HV. Xylan biosynthesis. *Curr Opin Biotech.* 2014,26:100-107.

166. Yu J, Hu F, Dossa K, Wang Z, Ke T. Genome-wide analysis of UDP-glycosyltransferase super family in *Brassica rapa* and *Brassica oleracea* reveals its evolutionary history and functional characterization. *BMC Genom.* 2017,18(1):474.

167. Hou J, Ye N, Dong ZY, Lu MZ, Li LG, Yin TM. Major chromosomal rearrangements distinguish willow and poplar after the ancestral "Salicoid" genome duplication. *Genome Biol Evol.* 2016,8:1868-1875.

168. Susek K, Bielski WK, Hasterok R, Naganowska B, Wolko B. A first glimpse of wild lupin karyotype variation as revealed by comparative cytogenetic mapping. *Front Plant Sci*. 2016,7:1152.

169. Ma X, Vaistij FE, Li Y, van Rensburg WSJ, Harvey S, Bairu MW, et al. A chromosome-level Amaranthus cruentus genome assembly highlights gene family evolution and biosynthetic gene clusters that may underpin the nutritional value of this traditional crop. *Plant J.* 2021,107:613-628.

170. Bennetzen JL. Transposable elements, gene creation and genome rearrangement in flowering plants. *Curr Opin Genet Dev.* 2005,15(6):621-627.

171. Kalendar R, Sabot F, Rodriguez F, Karlov GI, Natali L, Alix K. Editorial: Mobile elements and plant genome evolution, comparative analyzes and computational tools. *Front. Plant Sci.* 2021,12:735134.

172. Lysák MA, Schubert I. Mechanisms of chromosome rearrangements. In: Greilhuber J, Dolezel J, Wendel J, editors. Plant Genome Diversity Volume 2. Vienna: Springer; 2013. p. 137-147

173. Barra V, Fachinetti D. The dark side of centromeres: types, causes and consequences of structural abnormalities implicating centromeric DNA. *Nat Commun.* 2018,9(1):4340.

174. Manova V, Gruszka D. DNA damage and repair in plants –from models to crops. *Front Plant Sci.* 2015,6:885.

175. Wood RD. DNA repaired in eukaryotes. *Annu Rev Biochem*. 1996,65:135-167.

176. Nisa M-U, Huang Y, Benhamed M, Raynaud C. The plant DNA damage response: signaling pathways leading to growth inhibition and putative role in response to stress conditions. *Front Plant Sci*. 2019,10:653.

177. *Musa beccarii* genome. *Banana Genome Hub.* 2022. https://banana-genome-hub.southgreen.fr/node/50/1440401.

178. Wang Z, Rouard M, Droc G, Heslop-Harrison JS, Ge X. Supporting data for "Genome assembly of *Musa beccarii* shows extensive chromosomal rearrangements and genome expansion during evolution of Musaceae genomes" *GigaScience Database*. 2023. http://dx.doi.org/10.5524/102347

Table 1. Statistics of the genome assembly of *Musa beccarii*

Contig statistics of initial assembly using Nanopore reads		Contig statistics of initial assembly using PacBio HiFi reads		Scaffold statistics after Hi-C scaffolding		Chromosome	Length
Length of the sequence (bp)	Order of the sequence length	Length of the sequence (bp)	Order of the sequence length	Length of the sequence (bp)	Order of the sequence length		
N10=48,080,317	L10=2	N10=8,700,004	L10=7	N10=79,885,826	L10=1	chr1	79,367,759
N20=39,700,570	L20=3	N20=5,180,184	L20=17	N20=79,367,759	L20=2	chr2	79,885,826
N30=27,992,656	L30=5	N30=3,933,706	L30=31	N30=73,517,995	L30=3	chr3	67,088,101
N40=21,895,089	L40=7	N40=3,192,498	L40=48	N40=73,517,995	L40=3	chr4	57,442,642
N50=18,949,966	L50=11	N50=2,546,178	L50=70	N50=67,088,101	L50=4	chr5	73,517,995
N60=15,652,116	L60=14	N60=2,007,927	L60=99	N60=60,040,564	L60=5	chr6	60,040,564
N70=12,145,816	L70=18	N70=1,507,786	L70=136	N70=57,442,642	L70=6	chr7	53,040,366
N80=8,091,256	L80=25	N80=1,059,843	L80=186	N80=53,040,366	L80=7	chr8	42,891,246
N90=1,849,914	L90=40	N90=527,812	L90=271	N90=42,891,246	L90=8	chr9	38,409,407
N100=21,817	L100=306	N100=12,368	L100=811	N100=1,000	L100=449		
Total length	607,623,222bp	63,669,4734bp		56,961,7942bp			
Average length	1,985,696.80bp	785,073.65bp		1,268,636.84bp			
Largest length	52,524,701bp	11,573,678bp		79,885,826bp			
Minimum length	21,817bp	12,368bp		1,000bp			

Table 2. Possible biosynthetic gene clusters identified in Musaceae species. Tomatine 1 and 2 are tomatine clusters that locate in different chromosomes when previously identified

Cluster	*M. beccarii*	*E. glaucum*	*M. balbisiana*	*M. acuminata*	*M. schizocarpa*
Saccharide	2	3	3	2	3
Solanum l tomatine 1	11	7	10	13	9
Solanum l tomatine 1- Saccharide	0	1	0	1	1
Solanum l tomatine 1-Tomatine 2	2	1	2	2	3
T3PKS	21	17	18	22	14
T3PKS-Saccharide	2	1	0	1	1
T3PKS-Solanum l tomatine 1	6	8	9	9	11
T3PKS-Solanum l tomatine 1-Tomatine 2	3	1	0	2	3
T3PKS-Terpene	0	0	1	1	0
T3PKS-Terpene-Solanum l tomatine 1	1	2	1	1	1
T3PKS-Tomatine 2	2	2	3	1	0
Terpene	3	6	8	8	7
Terpene-Solanum l tomatine 1	3	5	2	2	2
Terpene-Solanum l tomatine 1-Tomatine 2	1	0	1	0	0
Tomatine 2	8	4	6	6	6
Other	1	0	0	1	0
Other-Solanum l tomatine 1-Tomatine 2	0	1	0	0	1
Total	66	59	64	72	62

Figure legends

Figure 1. Picture of a *Musa beccarii* flower

Figure 2. A) Hi-C interaction heat map (bin length 10,000 bp) for the *Musa beccarii* genome; B) Genome features across *M. beccarii* chromosomes. C) Inferred phylogenetic tree and contracted (–) and expanded (+) gene families in *M. beccarii* and other species in Liliopsida. Gene families within the most recent common ancestor (MRCA) are denoted at the root. Numbers following each species indicate the statistics of different genes

Figure 3. A) Number and B) length of transposable elements in Musaceae species. LTR: long terminal repeats; TIR: terminal inverted repeats; nonTIR: non-terminal inverted repeats

Figure 4. A) Density distribution of synonymous nucleotide substitutions (*Ks*) in whole genome duplication analysis; B) Speciation event (red line) detection using the rate-adjusted *Ks* distribution for *Musa beccarii* with the ksrates package. The background was the whole-paranome *Ks* distribution (light gray histogram and KDE curve) and anchor-pair *Ks* distribution (dark gray histogram and KDE curve) for *M. beccarii*. The shared number in the red circle indicates the same speciation event between *M. beccarii* and the other *Musa* species. The numbers and arrows in the parentheses of the four *Musa* species in the panel legend indicate *Ks* value shifts after substitution rate adjustments by ksrates; C) Syntenic blocks between Musaceae species. The largest blocks in *Musa* are highlighted in orange; D) Biosynthetic gene clusters (BGSs) in chr4 in *M. beccarii* and gene synteny with the other three *Musa* species in their chr4s. The regulatory genes in the BGCs are not shown

Figure 5. Dot plots of *Musa beccarii* and the other four species in Musaceae made using D-GENIES. The dot colors correspond to the similarity values, which are binned into four groups. Highly conserved chromosomes between *M. beccarii* and the other *Musa* genomes are highlighted in red boxes

Figure 6. The chromosomal history of the Musaceae genomes shows changes in the genome structure from the last common ancestor (LCA, the topmost one above the genome in the picture) to three intermediate ancestors and five studied species. The genes in the LCA are represented with lines and the same colors if they are in the same contigs, with the exception of genes in the contigs containing less than 100 genes, which are stacked into one super contig and all colored in black. The genes in the intermediate ancestors and in the studied species are colored based on orthologous genes in the LCA (determined by reciprocal best hits in SynChro analysis); otherwise, they are white to indicate the lack of homology with genes in the LCA.

Additional Files

Supplementary Table S1. Libraries for genome assembly and annotation for *Musa beccari*

Supplementary Table S2. Protein sequences of three species used for gene prediction

Supplementary Table S3. Species used for comparative genomics

Supplementary Table S4. Species pairs and their estimated divergence times used for time calibration points to infer the time-calibrated phylogeny of *Musa beccarii*

Supplementary Table S5. Genome size (bp) estimation using different programs

Supplementary Table S6. Assembly results using different assemblers

Supplementary Table S7. Repeat content of assemblies of Musaceae

Supplementary Table S8. Summary of gene functional annotations of the *Musa beccarii* genome using different databases

Supplementary Table S9. Summary of genes in Musaceae and the other compared species

Supplementary Table S10. Summary of MYB transcription factors identified by MYB_annotator, and their comparison with OrthoFinder results

Supplementary Table S11. MYB genes identified by MYB_annotator in *Musa beccarii*

Supplementary Table S12. Summer of MYB gene functions

Supplementary Table S13. Statistics of gene families in different species

Supplementary Table S14. GO enrichment results for Musaceae-specific gene families in *Musa*

beccarii

Supplementary Table S15. KEGG enrichment results for Musaceae-specific gene families in *Musa beccarii*

Supplementary Table S16. GO enrichment results for significantly expanded gene families

Supplementary Table S17. KEGG enrichment results for significantly expanded gene families

Supplementary Table S18. GO enrichment results for significantly contracted gene families

Supplementary Table S19. KEGG enrichment results for significantly contracted gene families

Supplementary Table S20. GO enrichment results for alternative splicing genes

Supplementary Table S21. KEGG enrichment results for alternative splicing genes

Supplementary Table S22. GO enrichment results for WGD type genes

Supplementary Table S23. KEGG enrichment results for WGD type genes

Supplementary Table S24. GO enrichment results for TD type genes

Supplementary Table S25. KEGG enrichment results for TD type genes

Supplementary Table S26. GO enrichment results for PD type genes

Supplementary Table S27. KEGG enrichment results for PD type genes

Supplementary Table S28. GO enrichment results for TRD type genes

Supplementary Table S29. GO enrichment results for DSD type genes

Supplementary Table S30. KEGG enrichment results for DSD type genes

Supplementary Table S31. Results of the syntenic block analysis

Supplementary Table S32. Biosynthetic gene clusters in Musaceae

Supplementary Table S33. NLR-Annotator results in *Musa beccarii*

Supplementary Table S34. NLR-Annotator results in Musaceae using their predicted genes and comparison of the results using the genomes

Supplementary Table S35. Structure variations (SVs) detected by SVanalyzer between *Musa beccarii* and *M. acuminata*

Supplementary Table S36. Summary of ABC transporter-related genes

Supplementary Table S37. Summary of glycosyl transferases genes

Supplementary Figure S1. A scheme showing *Musa beccarii* genome assembly

Supplementary Figure S2. A) Nanica repetitive sequences and B) NBS-LRR genes identified by NLR-annotator across the *Musa beccarii* genome

Supplementary Figure S3. Treemap showing hierarchy for GO terms enriched for Musaceae-specific gene families of *Musa beccarii* in biological process

Supplementary Figure S4. Upset plot showing the intersection of the gene family in *Musa*. *Musa* species are presented in the rows and the bar beside each species shows its total gene number. Black circles and vertical lines between the rows represent the intersection of gene families between species. Barplot indicates the total gene family count in each intersection

Supplementary Figure S5. Summary of GO annotations of Musaceae shared genes

Supplementary Figure S6. Treemap showing hierarchy for GO terms enriched with significantly expanded gene families in biological process

Supplementary Figure S7. Treemap showing hierarchy for GO terms enriched with alternative splicing genes in biological process

Supplementary Figure S8. Treemap showing hierarchy for GO terms enriched with genes related to WGD in biological process

Supplementary Figure S9. KEGG enrichment for genes related to A) whole genome duplication; B) tandem duplications; C) proximal duplications; D) dispersed duplications tested by DupGen_Finder

Supplementary Figure S10. Treemap showing the hierarchy for GO terms enriched with genes related to tandem duplications in biological process

Supplementary Figure S11. Treemap showing the hierarchy for GO terms enriched with genes related to dispersed duplications in biological process

Supplementary Figure S12. Synteny plot (Synvisio) between *Ensete glaucum* (egxx) and *Musa acuminata* (mpxx), *Musa beccarii* (bexx) genomes. Syntenic blocks of high homology are indicated by uniformly colored areas in the graphs

Supplementary Figure S13. Coverage distributions of Illumina WGS reads mapping against *Musa beccarii* assembly

Supplementary Figure S14. Histograms showing the allele frequencies of SNVs (single nucleotide variants) obtained from the Illumina WGS reads mapping against the *Musa beccarii* assembly

Supplementary Figure S15. KEGG pathway of flavonoid biosynthesis. In the pathway, the genes identified in *Musa beccarii* are boxed in green or pink colors.

Figure_1

Click here to access/download;Figure;Figure_1.tif

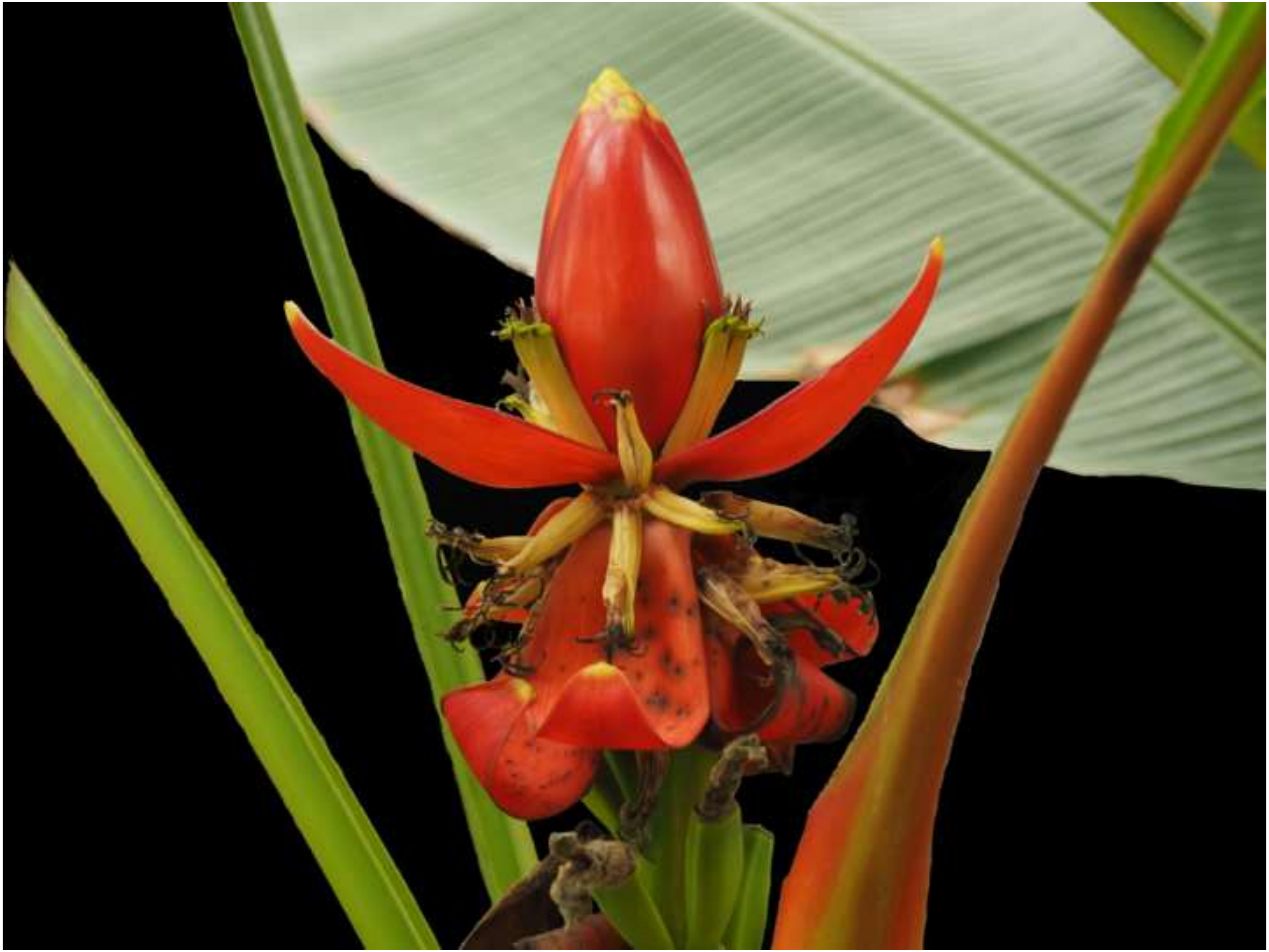

Click here to access/download;Figure;Figure_2.tif

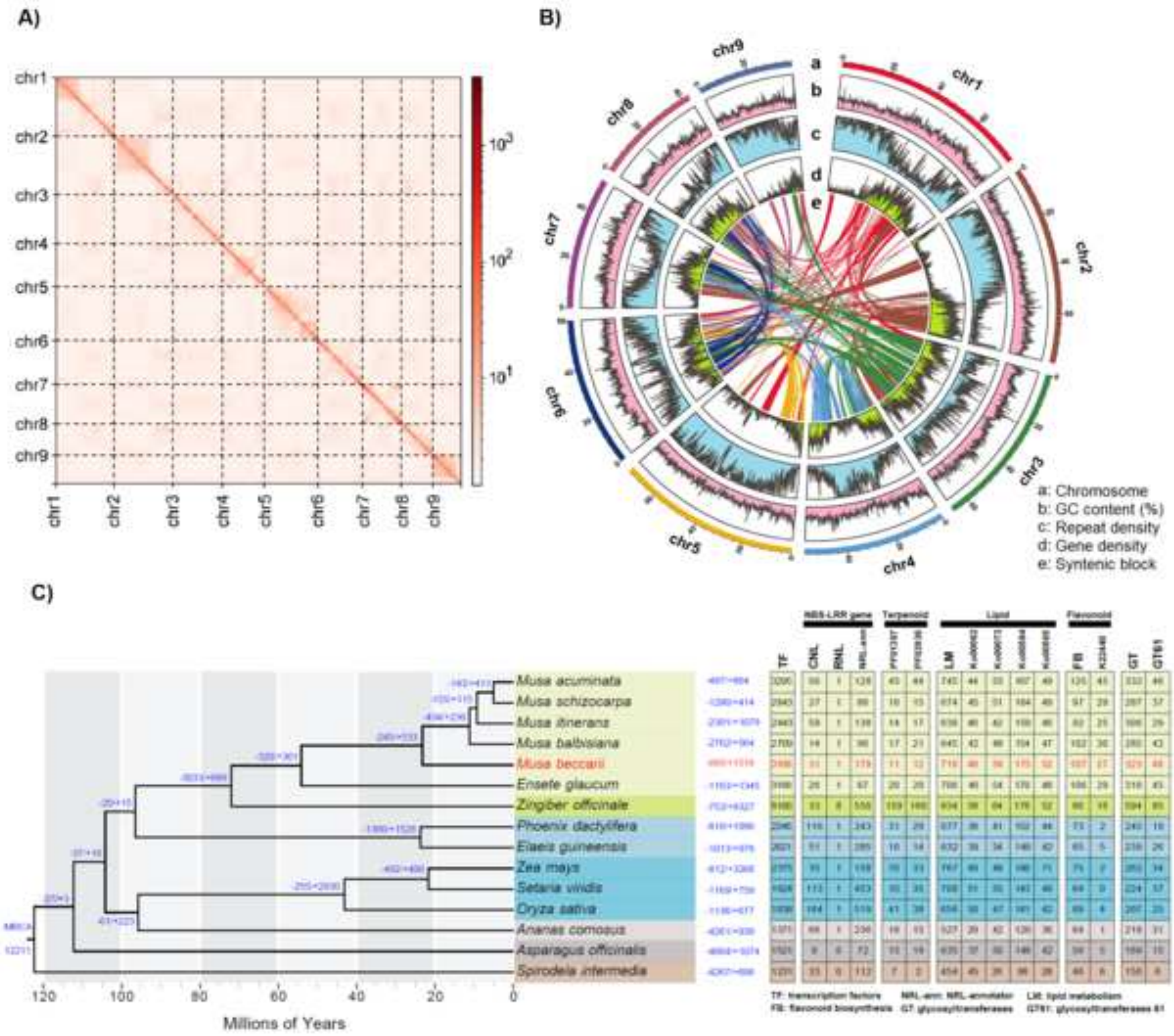

Figure_3 Click here to access/download;Figure;Figure_3.tif

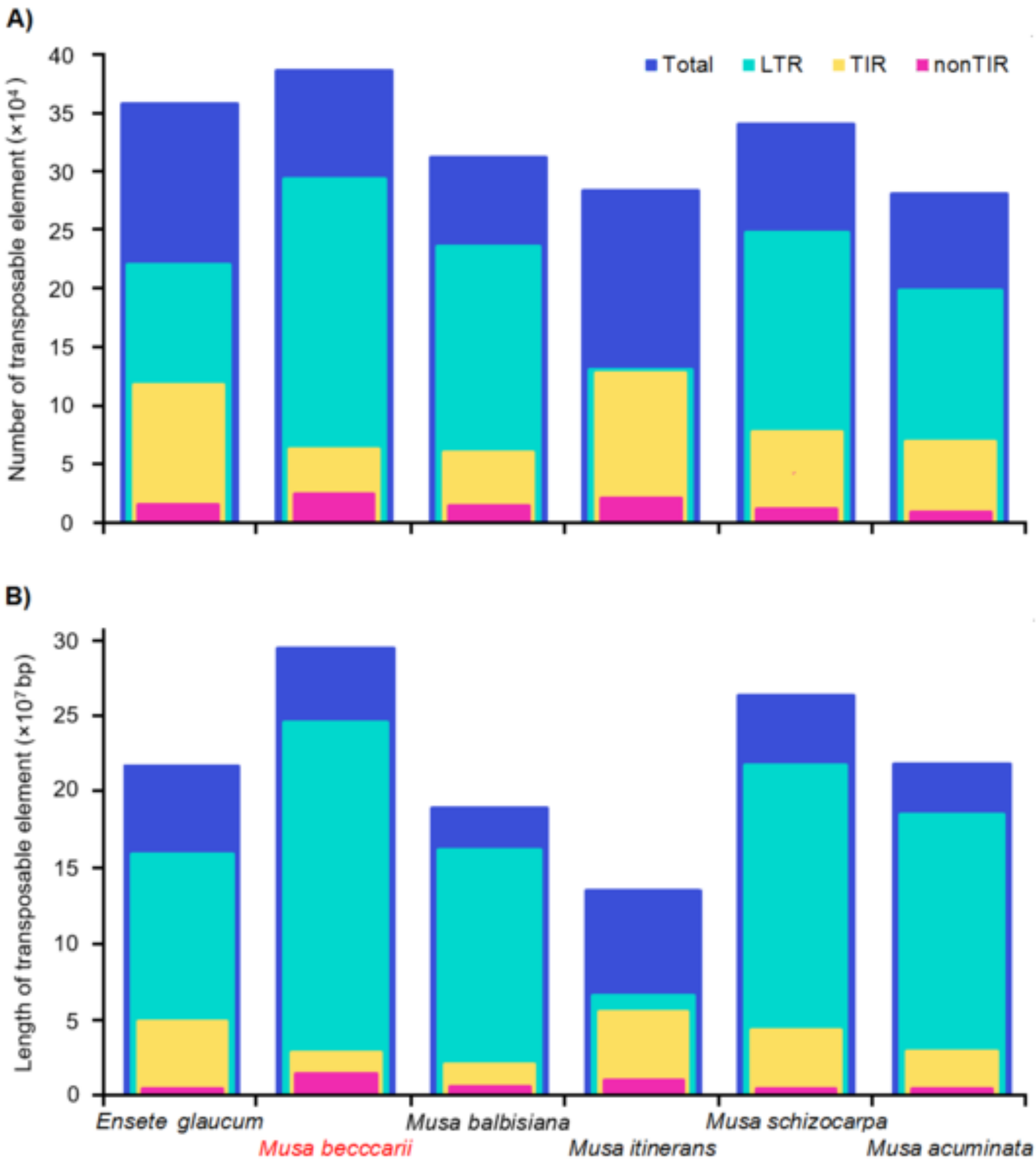

Figure_4

Click here to access/download;Figure;Figure_4.tif

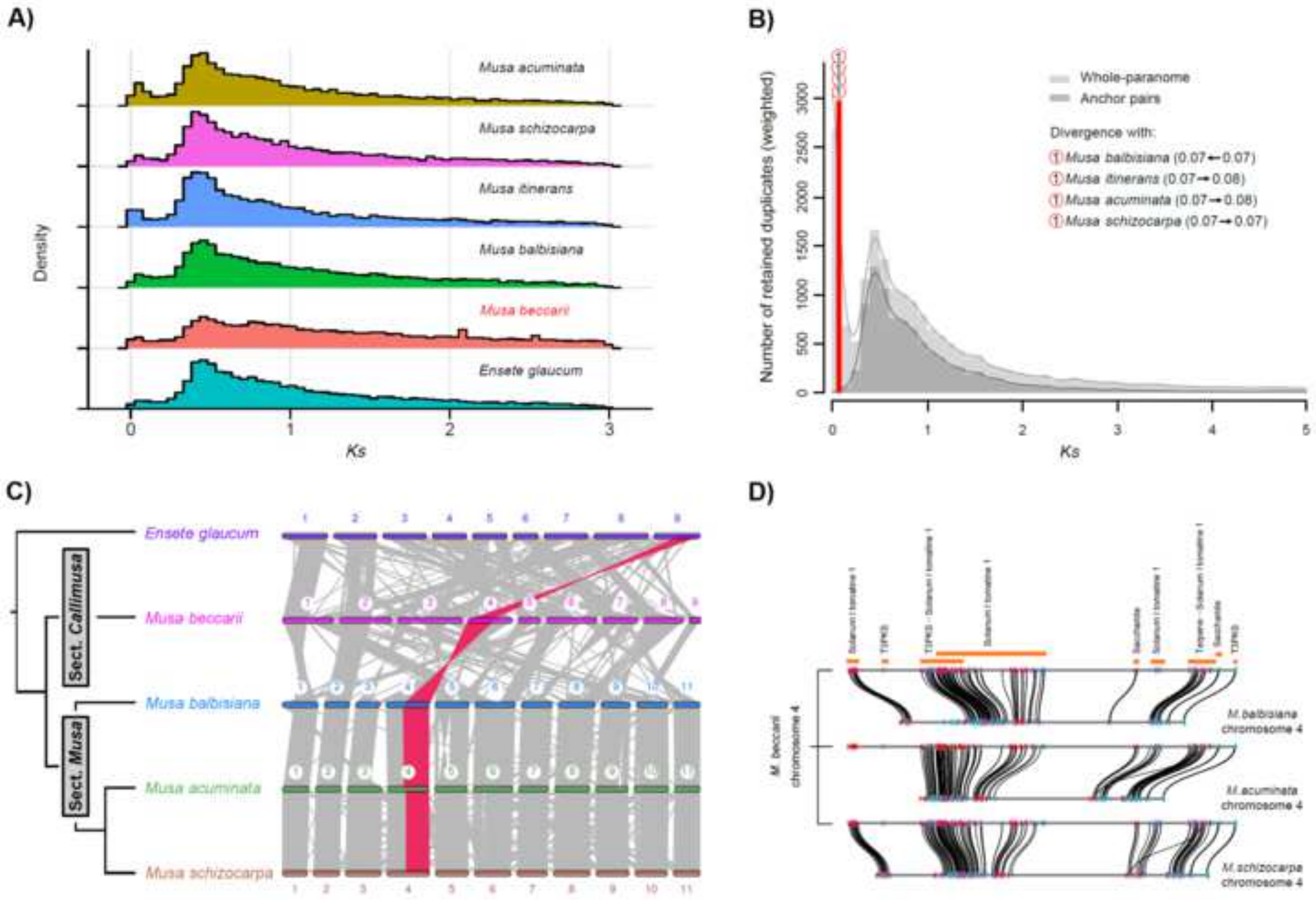

Figure_5

Click here to access/download;Figure;Figure_5.tif

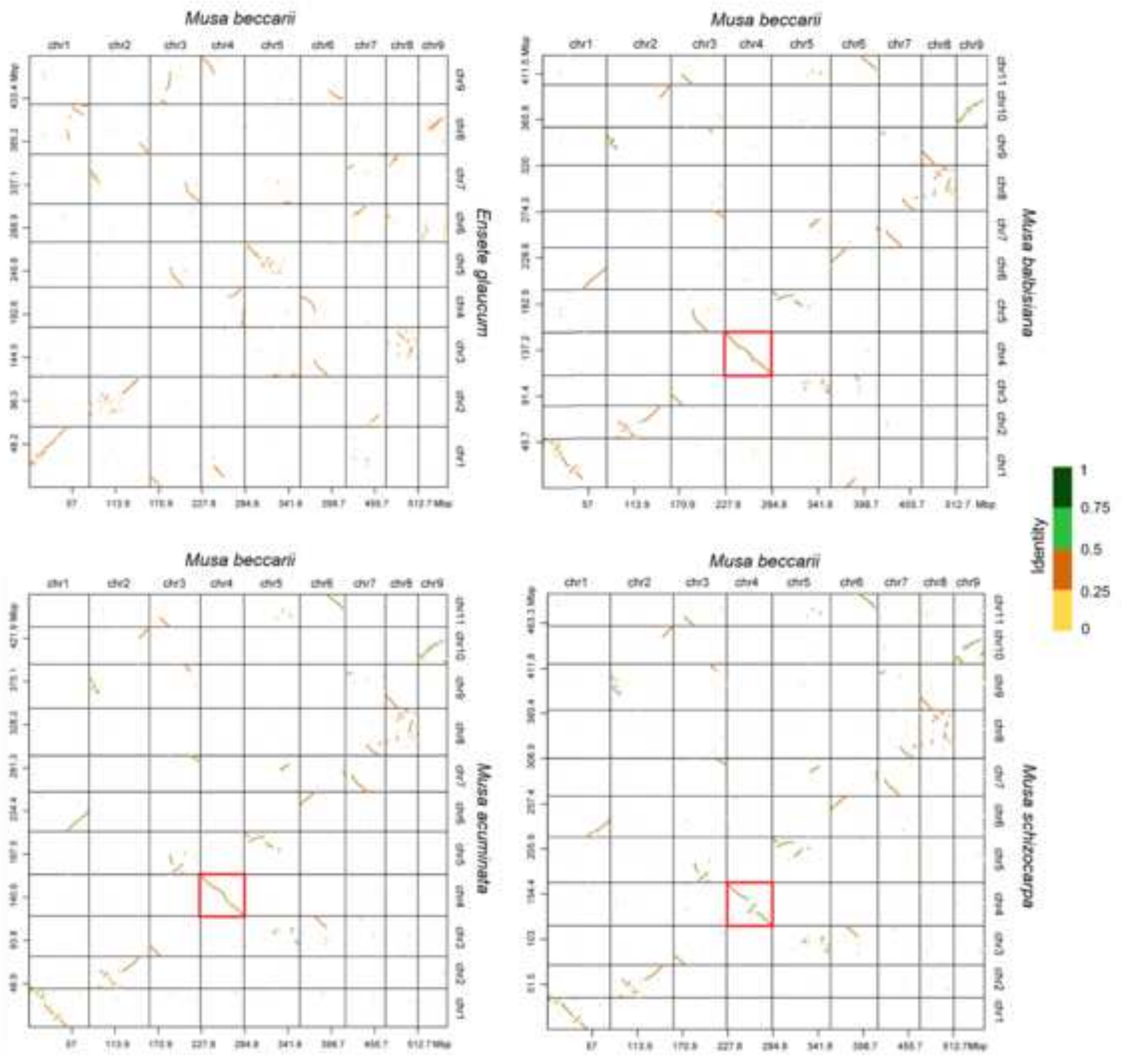

Click here to access/download;Figure;Figure_6.tif

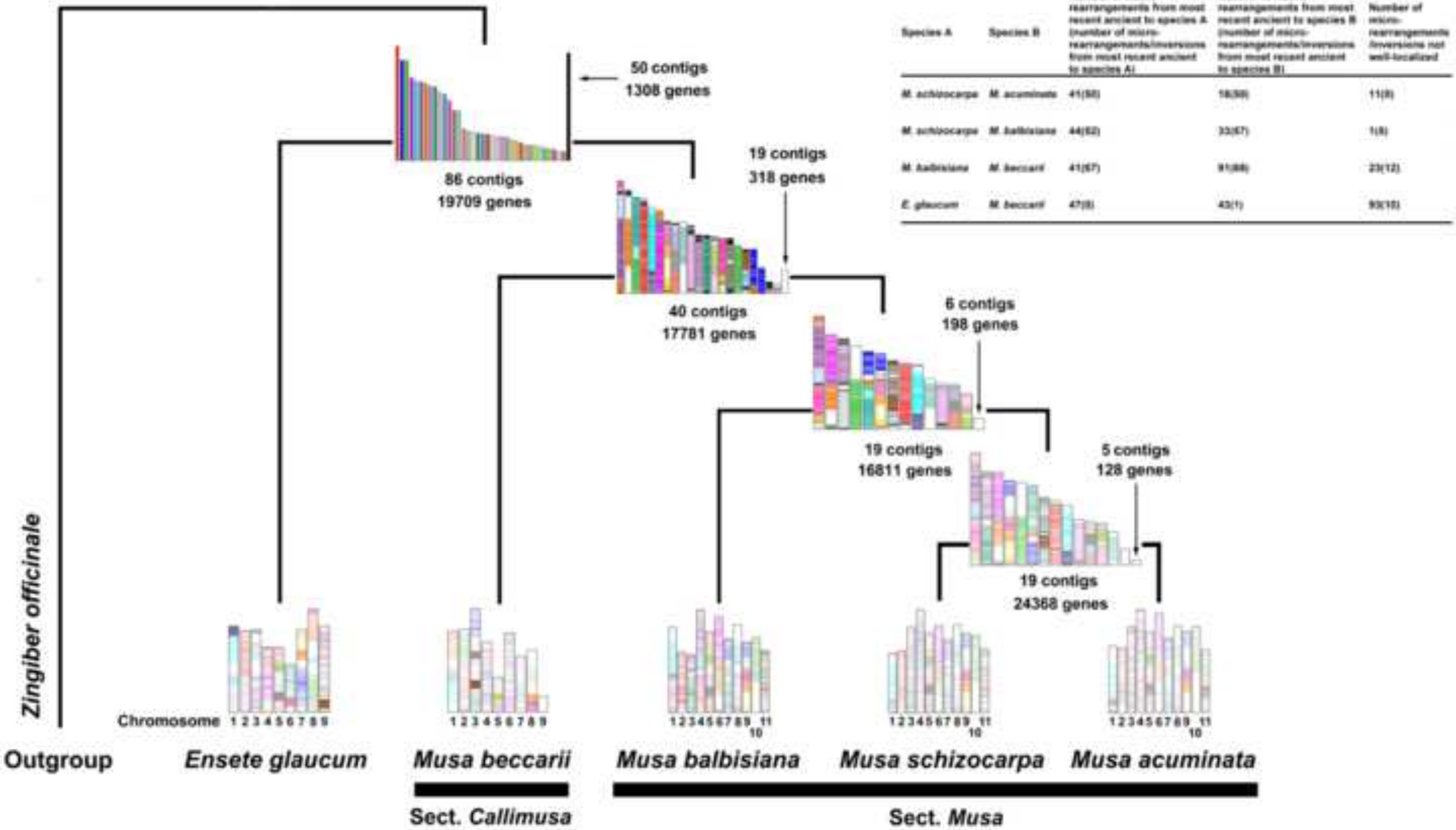

Species A	Species B	Number of macro-rearrangements from most recent ancient to species A (number of micro-rearrangements/inversions from most recent ancient to species A)	Number of macro-rearrangements from most recent ancient to species B (number of micro-rearrangements/inversions from most recent ancient to species B)	Number of micro-rearrangements /inversions not well-localized
M. schizocarpa	*M. acuminata*	41(50)	18(50)	11(8)
M. schizocarpa	*M. balbisiana*	44(52)	33(67)	1(8)
M. balbisiana	*M. beccarii*	41(67)	91(88)	23(12)
E. glaucum	*M. beccarii*	47(8)	43(1)	93(10)

Figure_S1

Click here to access/download
Supplementary Material
Figure_S1.tif

Figure_S2

Click here to access/download
Supplementary Material
Figure_S2.tif

Figure_S3

Click here to access/download
Supplementary Material
Figure_S3.pdf

Figure_S4

Click here to access/download
Supplementary Material
Figure_S4.tiff

Figure_S5

Click here to access/download
Supplementary Material
Figure_S5.tif

Figure_S6

Click here to access/download
Supplementary Material
Figure_S6.pdf

Figure_S7

Click here to access/download
Supplementary Material
Figure_S7.pdf

Figure_S8

Click here to access/download
Supplementary Material
Figure_S8.pdf

Figure_S9

Click here to access/download
Supplementary Material
Figure_S9.tiff

Figure_S10

Click here to access/download
Supplementary Material
Figure_S10.pdf

Figure_S11

Click here to access/download
Supplementary Material
Figure_S11.pdf

Figure_S12

Click here to access/download
Supplementary Material
Figure_S12.tif

Figure_S13

Click here to access/download
Supplementary Material
Figure_S13.tif

Figure_S14

Click here to access/download
Supplementary Material
Figure_S14.tif

Figure_S15

Click here to access/download
Supplementary Material
Figure_S15.png

Supplymentary_tableS1-S36

Click here to access/download
Supplementary Material
supplymentary_tableS1-S36.xlsx

manuscript tracked

Click here to access/download
Supplementary Material
ms-2nd-2022-12-18-traked.docx
