# Supplementary figures and images for: Genome assembly of Musa beccarii shows extensive chromosomal rearrangements and genome expansion during evolution of Musaceae genomes
Source: Gigascience. 2023 Feb 21;12:giad005. doi: 10.1093/gigascience/giad005 (PMC9941839; doi:10.1093/gigascience/giad005)

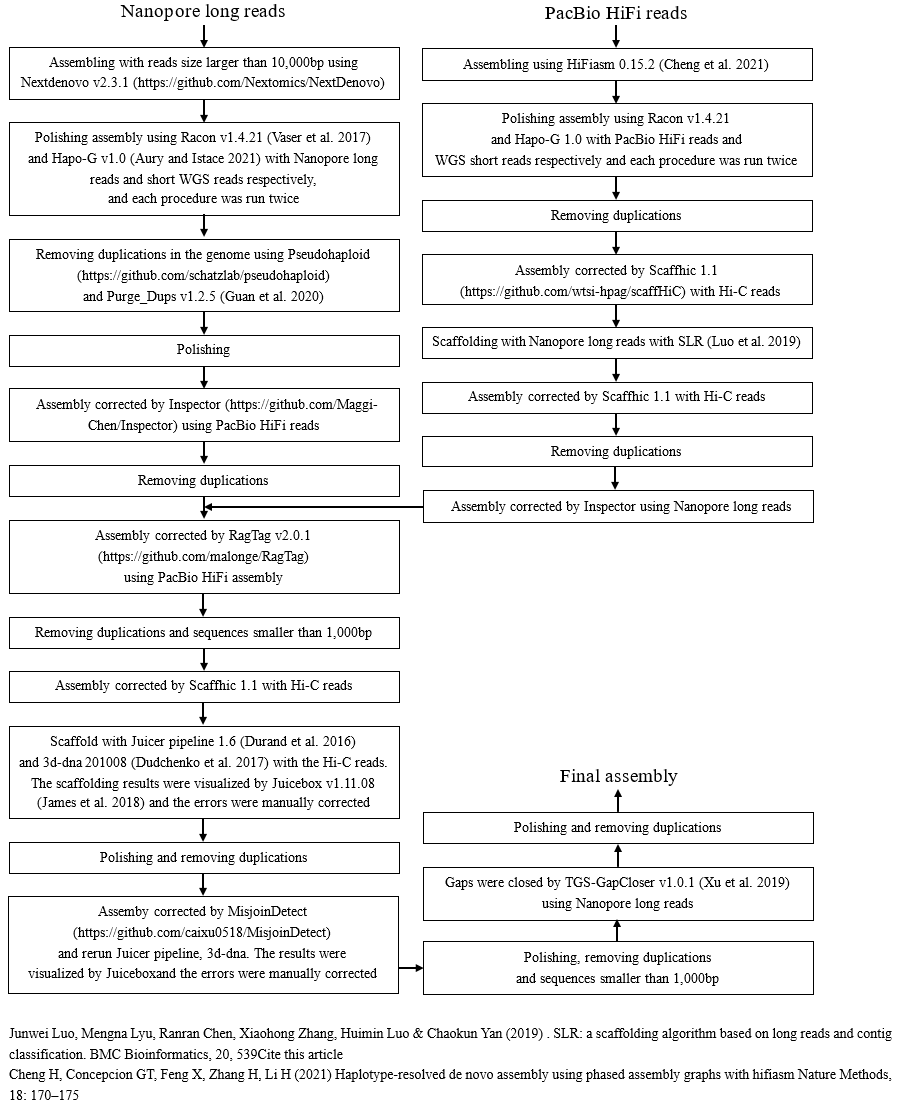

Supplement: giad005_Supplemental_Figures_and_Tables [file giad005_supplemental_figures_and_tables.zip › Figure_S1.tif]

Revigo TreeMap

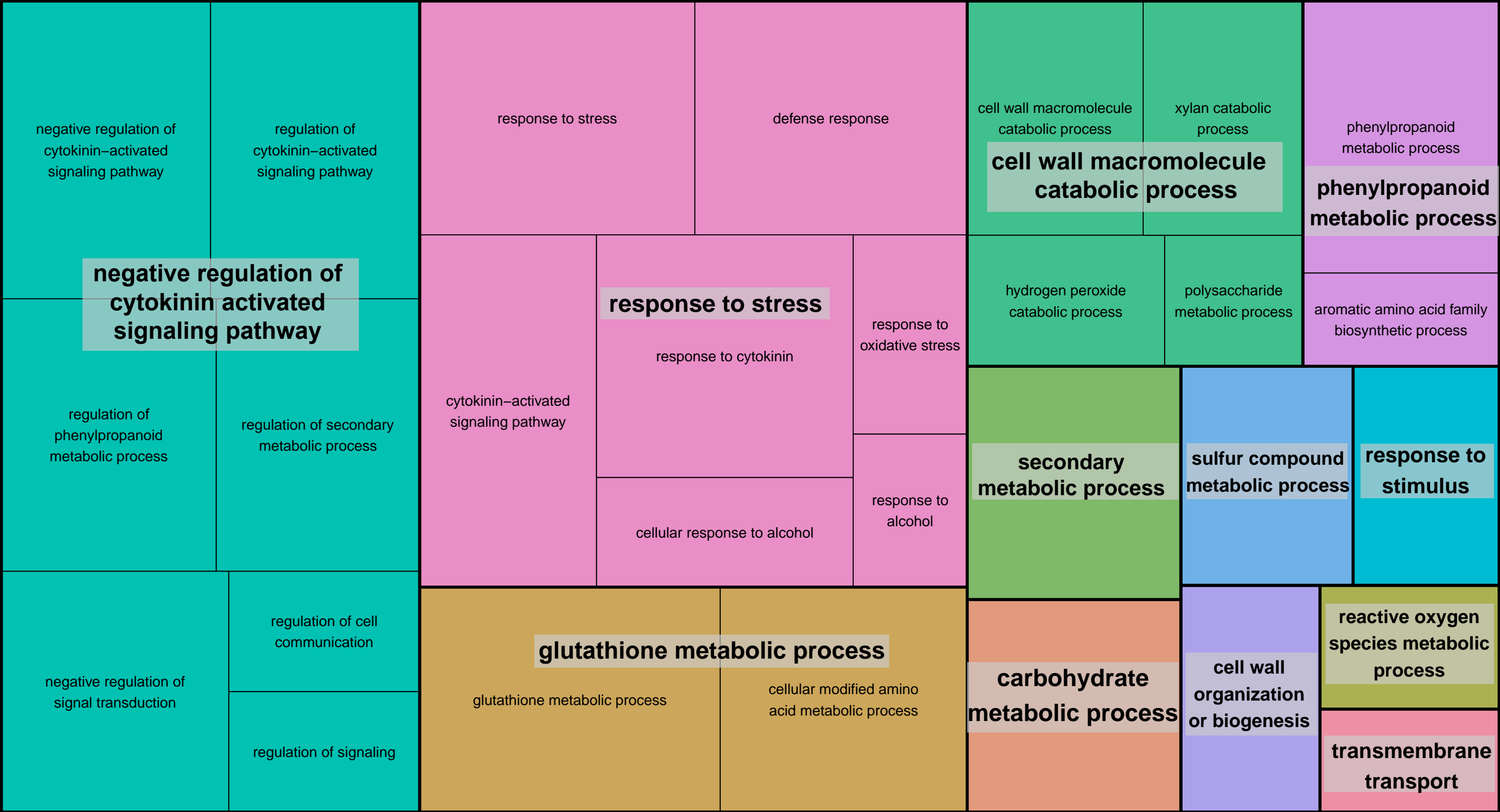

Supplement: giad005_Supplemental_Figures_and_Tables [file giad005_supplemental_figures_and_tables.zip › Figure_S10.pdf]

Revigo TreeMap

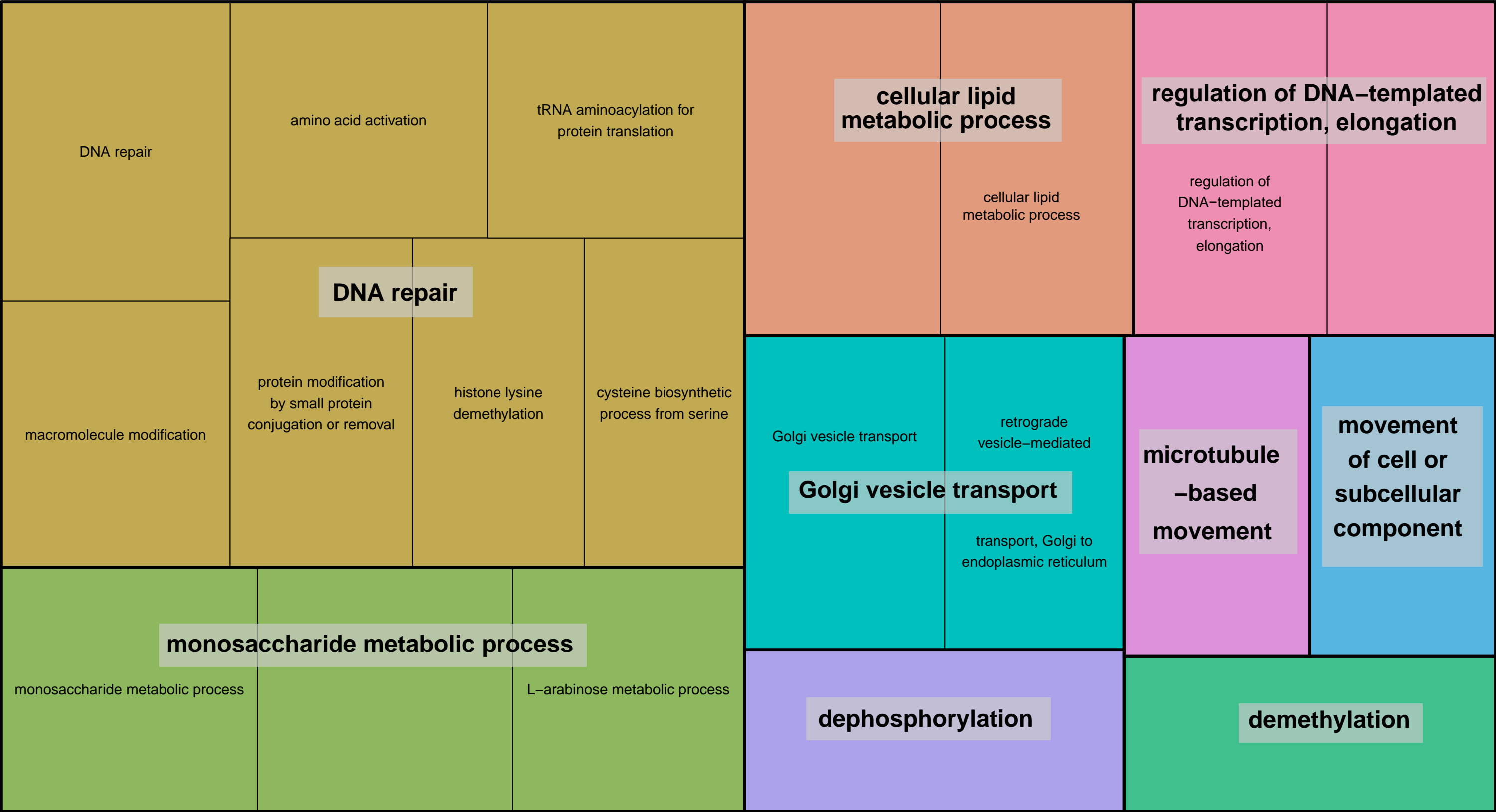

Supplement: giad005_Supplemental_Figures_and_Tables [file giad005_supplemental_figures_and_tables.zip › Figure_S11.pdf]

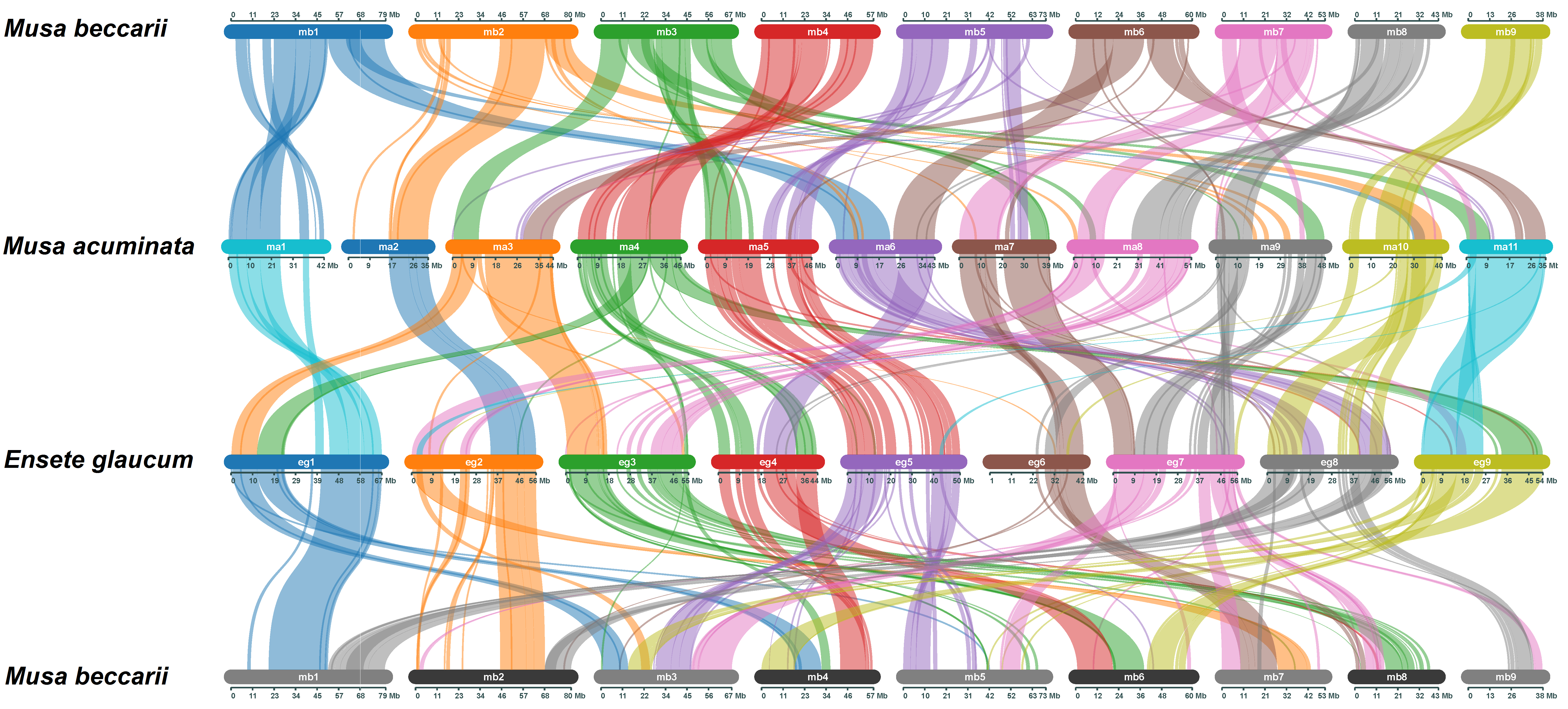

Supplement: giad005_Supplemental_Figures_and_Tables [file giad005_supplemental_figures_and_tables.zip › Figure_S12.tif]

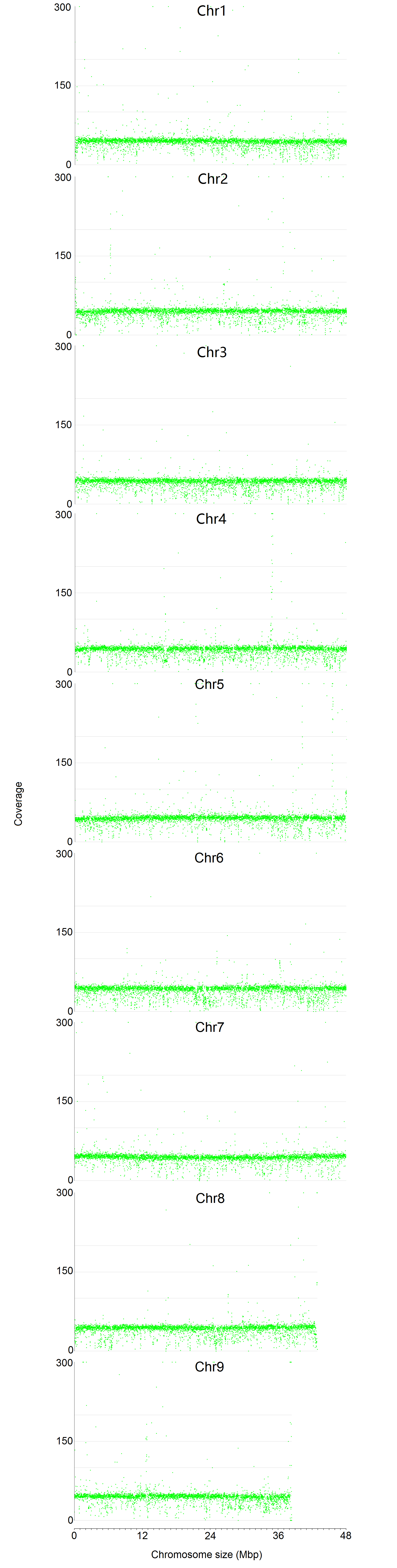

Supplement: giad005_Supplemental_Figures_and_Tables [file giad005_supplemental_figures_and_tables.zip › Figure_S13.tif]

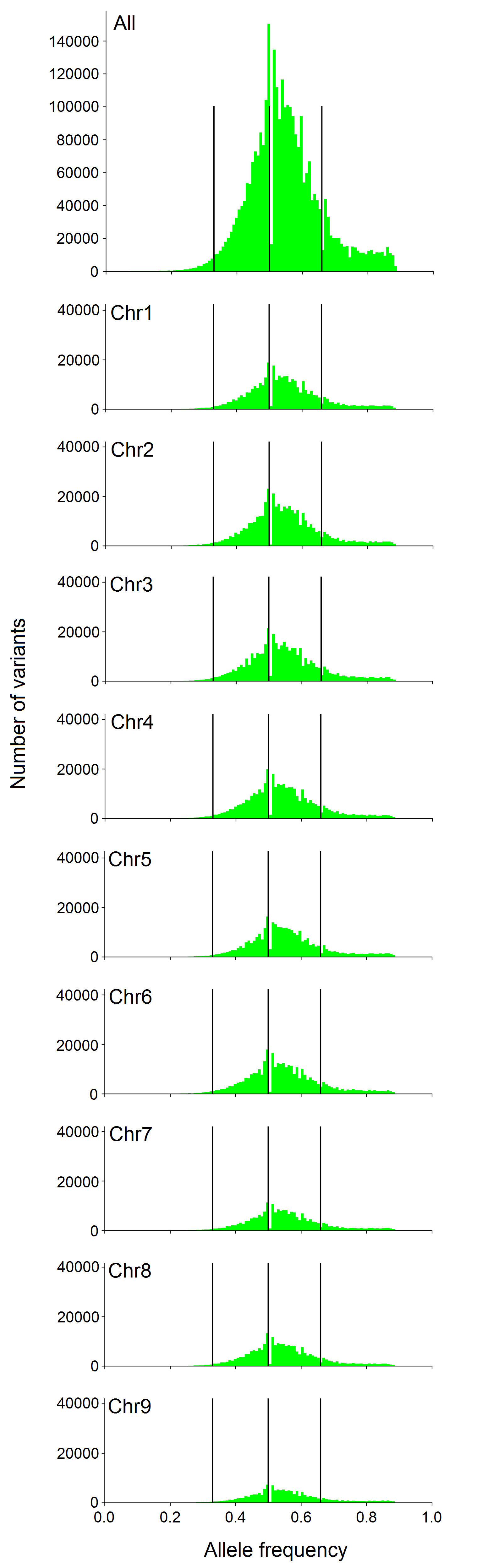

Supplement: giad005_Supplemental_Figures_and_Tables [file giad005_supplemental_figures_and_tables.zip › Figure_S14.tif]

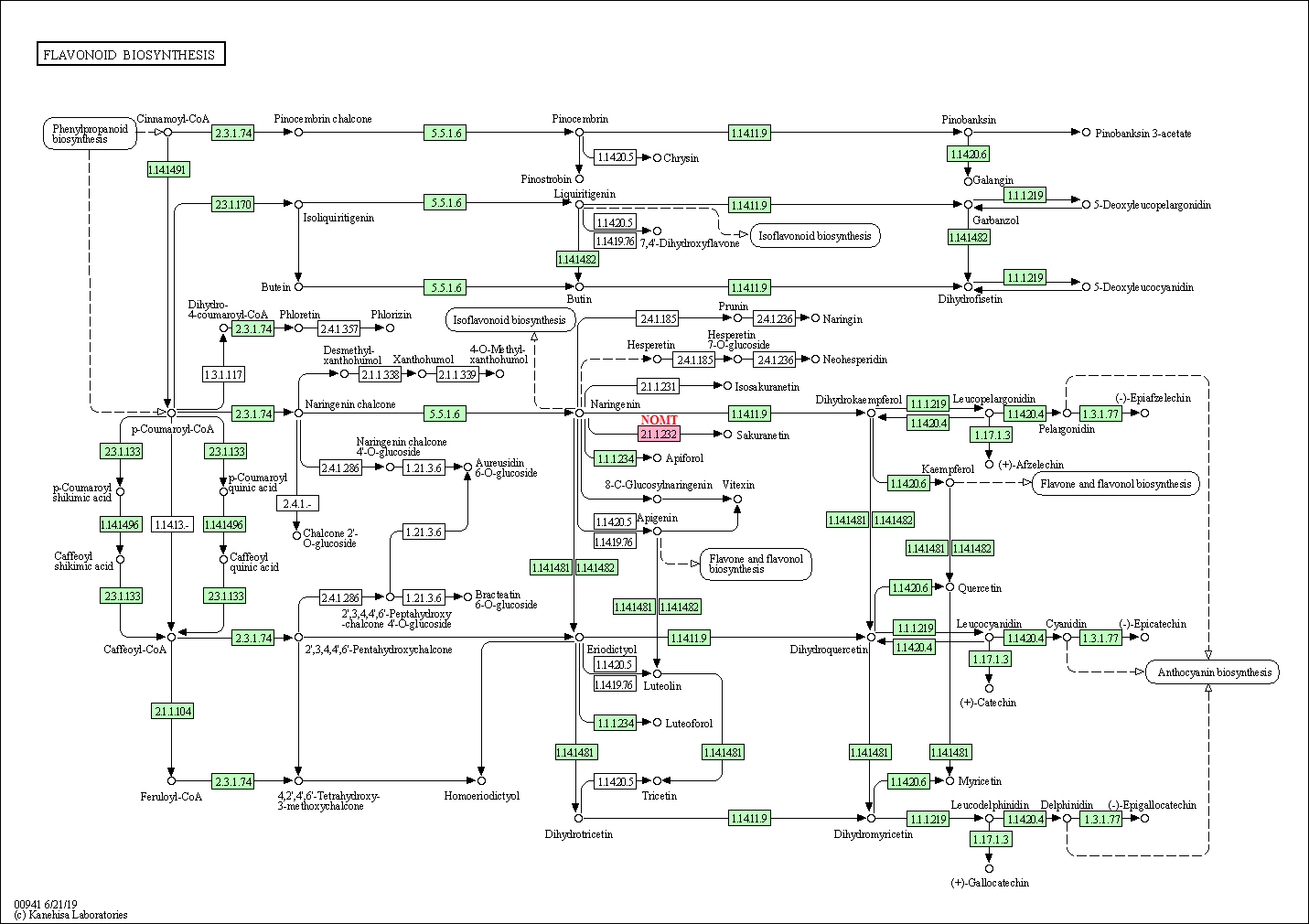

Supplement: giad005_Supplemental_Figures_and_Tables [file giad005_supplemental_figures_and_tables.zip › Figure_S15.png]

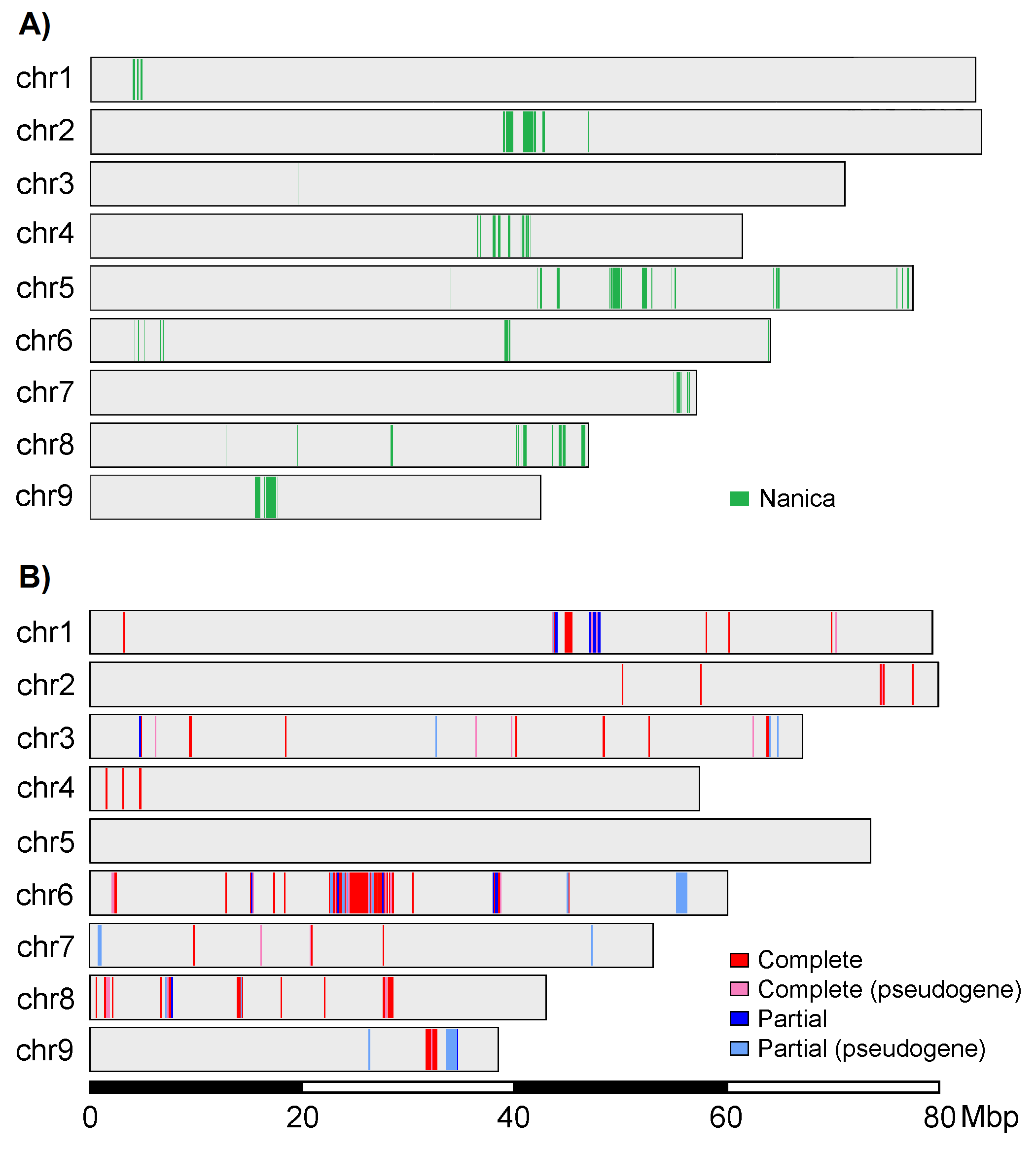

Supplement: giad005_Supplemental_Figures_and_Tables [file giad005_supplemental_figures_and_tables.zip › Figure_S2.tif]

Revigo TreeMap

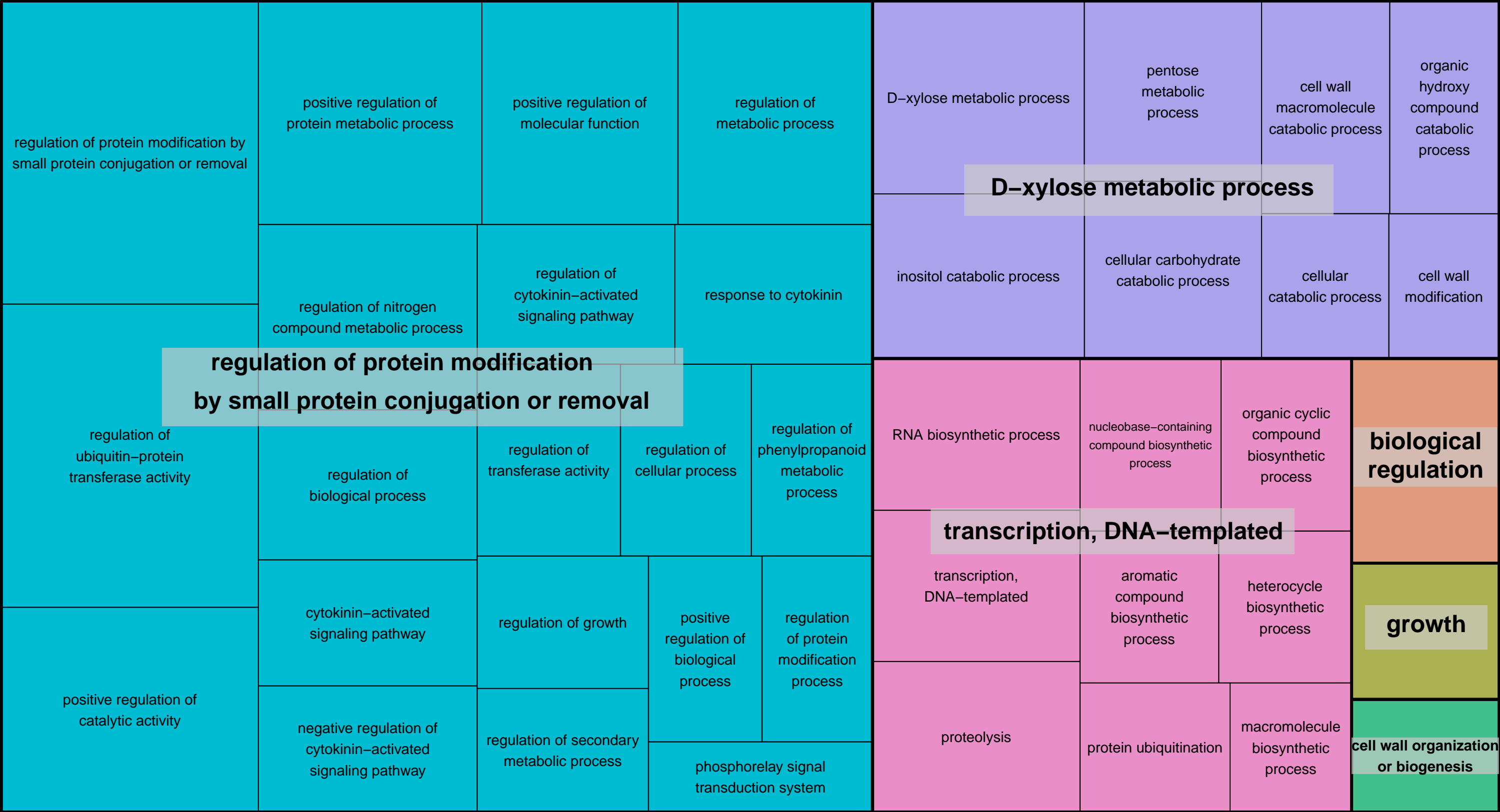

Supplement: giad005_Supplemental_Figures_and_Tables [file giad005_supplemental_figures_and_tables.zip › Figure_S3.pdf]

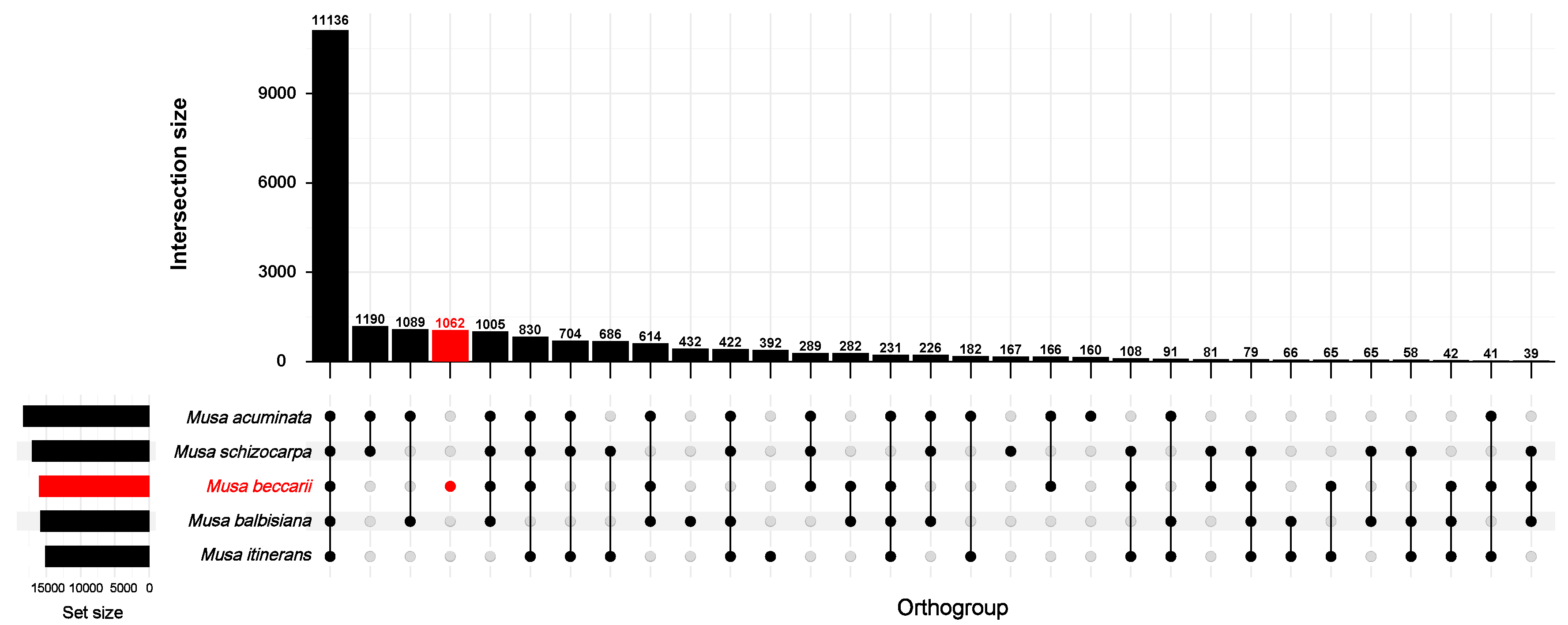

Supplement: giad005_Supplemental_Figures_and_Tables [file giad005_supplemental_figures_and_tables.zip › Figure_S4.tiff]

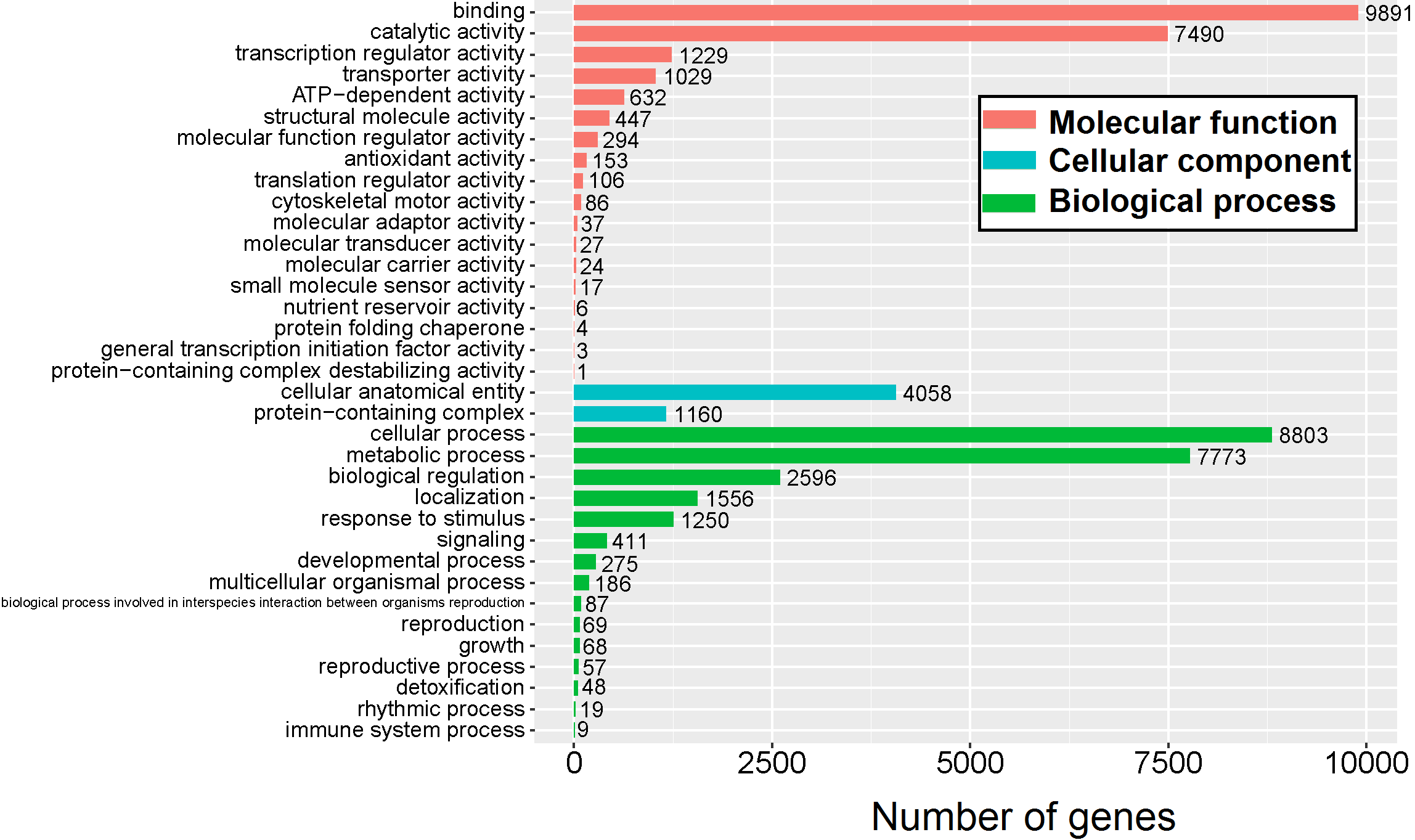

Supplement: giad005_Supplemental_Figures_and_Tables [file giad005_supplemental_figures_and_tables.zip › Figure_S5.tif]

Revigo TreeMap

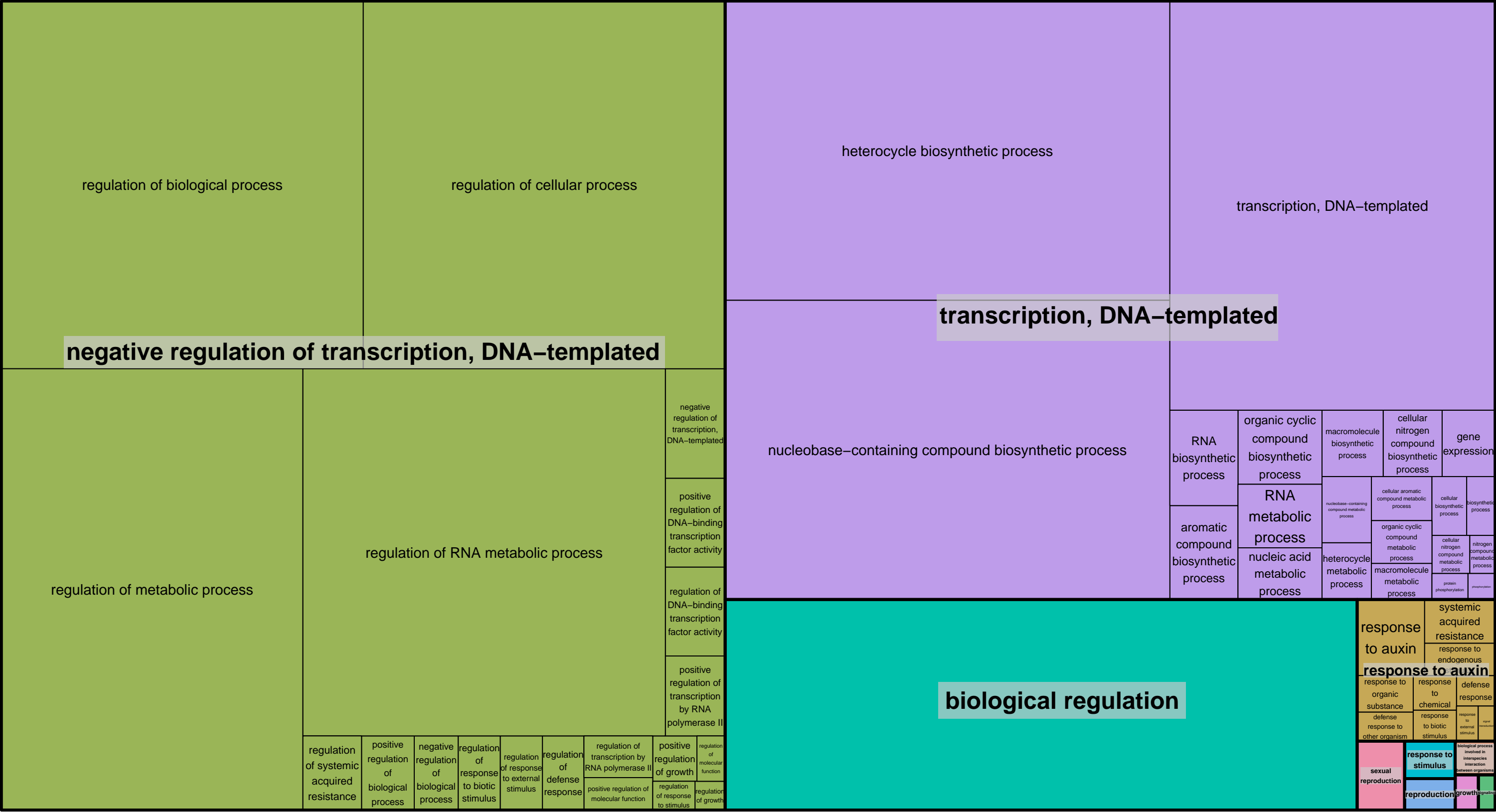

Supplement: giad005_Supplemental_Figures_and_Tables [file giad005_supplemental_figures_and_tables.zip › Figure_S6.pdf]

Revigo TreeMap

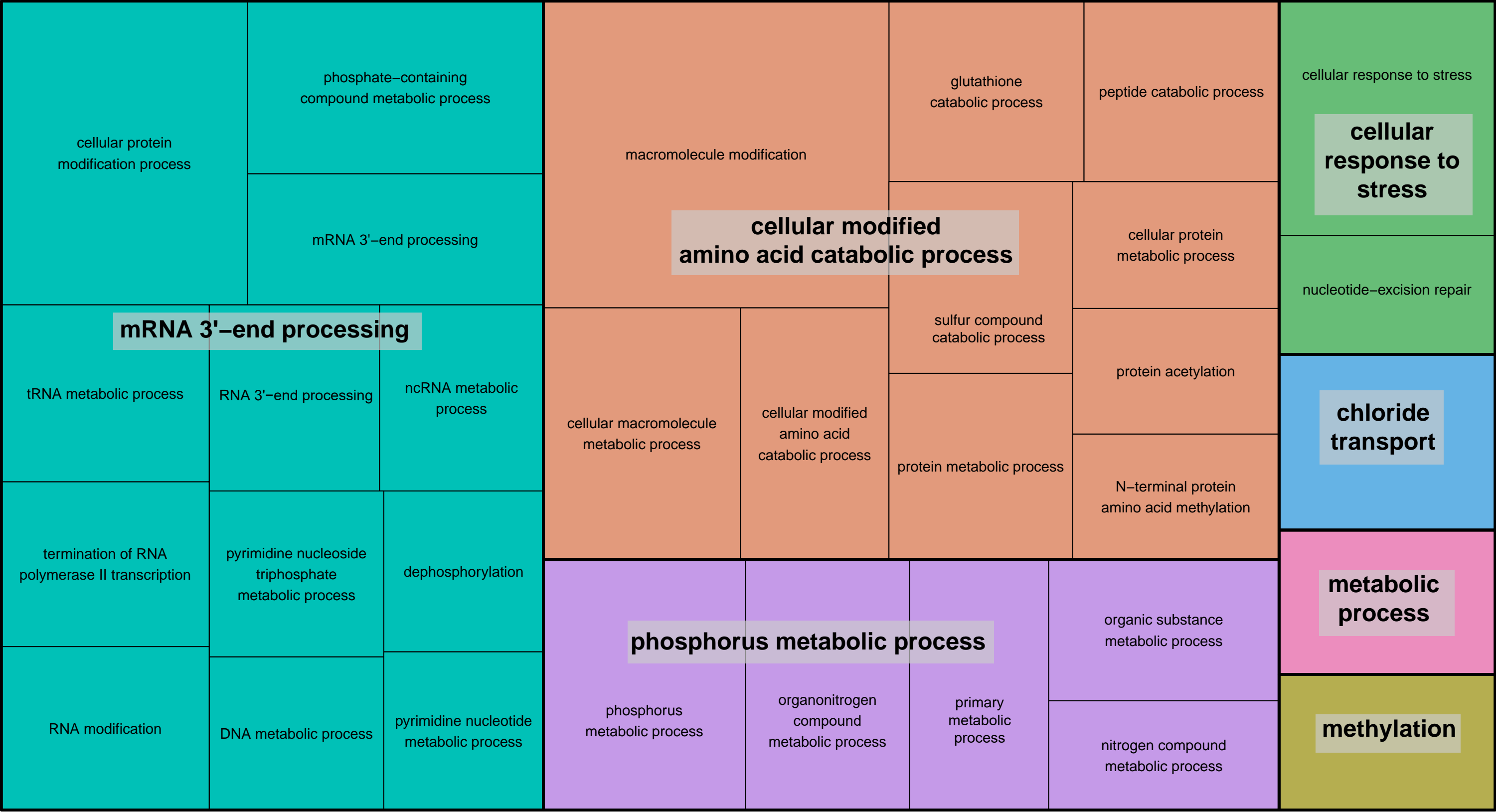

Supplement: giad005_Supplemental_Figures_and_Tables [file giad005_supplemental_figures_and_tables.zip › Figure_S7.pdf]

Revigo TreeMap

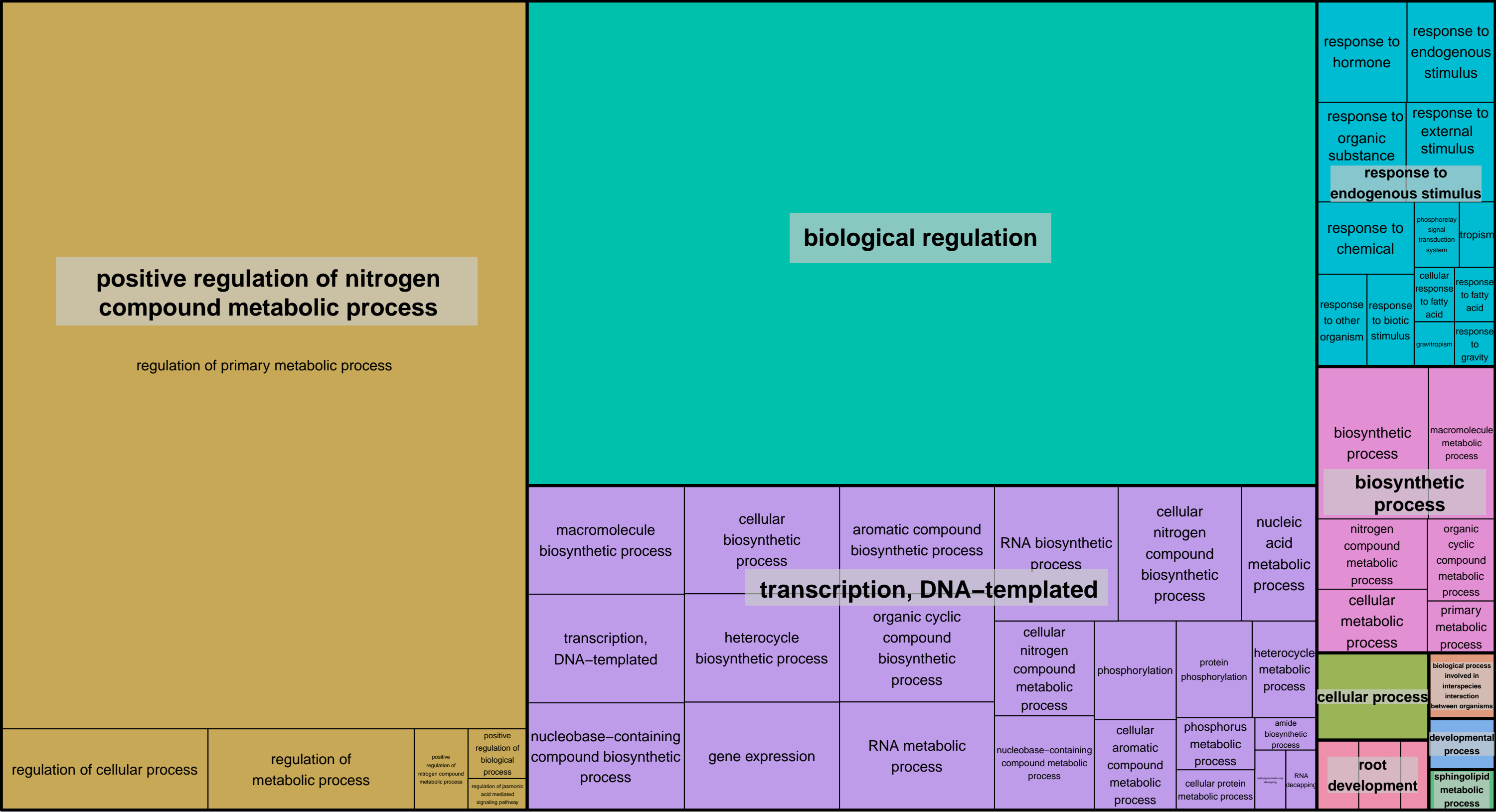

Supplement: giad005_Supplemental_Figures_and_Tables [file giad005_supplemental_figures_and_tables.zip › Figure_S8.pdf]

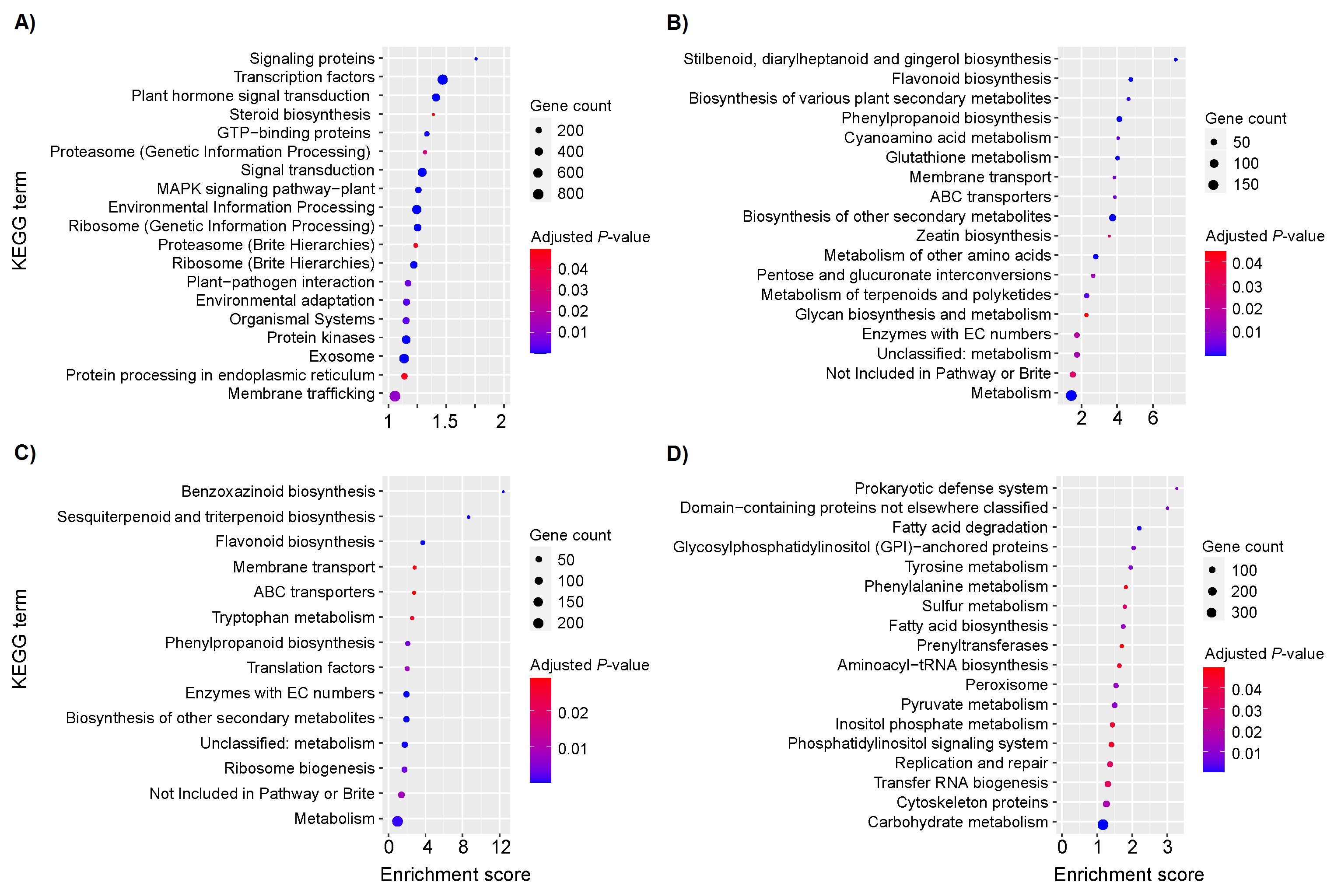

Supplement: giad005_Supplemental_Figures_and_Tables [file giad005_supplemental_figures_and_tables.zip › Figure_S9.tiff]
