# Supplementary material for: Genome assembly of Musa beccarii shows extensive chromosomal rearrangements and genome expansion during evolution of Musaceae genomes
Source: Gigascience. 2023 Feb 21;12:giad005. doi: 10.1093/gigascience/giad005 (PMC9941839; doi:10.1093/gigascience/giad005)
Supplement: giad005_Response_to_Reviewer_Comments_Original_Submission [file giad005_response_to_reviewer_comments_original_submission.pdf]

Author's Response To Reviewer Comments

Close

December 24, 2022
GigaScience

Dear editor,
Thank you very much for your decision letter about our manuscript entitled “Genome assembly of Musa beccarii shows extensive chromosomal rearrangements and genome expansion during evolution of Musaceae genomes”(No. GIGA-D-22-00219), including the comments.
We are now sending our responses to comments with our revised manuscript (both tracked and clean versions). All the modified parts in the revised manuscript are marked in red.
Our specific responses are as follows:

Reviewer(s)' Comments to Author:

Reviewer: 1
Wang et al. describe the genome sequence of Musa beccarii. The assembly is presented and compared against existing genome sequences. This additional genome sequence provides further insights into the evolution of Musaceae. All data sets were submitted to the corresponding repositories and will be shared via the banana genome hub. This work is certainly of interest to the community and the availability of all data sets will facilitate reuse in future studies. While the data set is of high quality, there are some (technical) issues that should be addressed. For example, the statements about the genome size should be checked and additional evidence for the contraction/expansion event would be helpful. Please find a list of specific comments below.

1) There is a difference between genome (DNA) and a genome sequence (information). This is particularly relevant when the genome size does not match the assembly size. Please adjust the use of both terms throughout the manuscript.
>>>We now replaced “genome” with “assembly” or “assembled genome” in the revised manuscript as suggested.

2) Do the authors have an approval from Malaysia to use this material for research and to publish genomic and transcriptomic data sets?
>>> Musa beccarii was named as a new species by Prof. Simmonds in 1960 based on a cultivated plant in Trinidad. M. beccarii was commonly cultivated in many botanical gardens across the world. Our material was retrieved from ex situ collection hosted in one European botanical garden, not directly from the wild, which arrived in Europe before 1993 and for which we got a transfer agreement. The material was used only for scientific research, not commercial purpose. Based on the non-retroactive principle of "Convention on Biological Diversity", this material was not constrained by the International Treaty on Plant Genetic Resources for Food and Agriculture.
Therefore, since the material was collected before Malaysia adopted a national ABS law, there is no need to get approval from Malaysia to use this material for research and to publish genomic and transcriptomic data sets. We now delete the sentence “The origin of this species was one botanical garden at Sabah, Borneo, Malaysia.” and briefly include above information in “Ethics Approval and Consent to Participate” section. Please see lines 691-692.

3) line 113: PacBio is not able to sequence RNAs. ONT is the only technology for high throughout RNA sequencing. What the authors are trying to describe is probably a cDNA sequencing run (with HiFi?). The short reads are most likely also derived from cDNAs.
>>>We agree with the reviewer. We have clarified it. Please see line 113-114 as follows: “total RNA from M. beccarii leaves of the same individual were extracted and reverse-transcribed to cDNA for PacBio full-

length RNA transcripts sequencing (Iso-Seq)".

4) Details of the conditions and time points of RNA extraction need to be added. Almost nothing is written about the samples used for the sequencing experiments. Please make sure to update the metadata (growth condition, plant part, developmental stage, ...) accordingly.
>>>We have included the information and updated the metadata of our submission to the GenBank (Biosample attributes tables). Please see lines 104-106. We deposited GenBank data (SRR16526886 for the Nanopore, and SRR16526885 for PacBio HiFi reads, SRR16526887 for the Illumina WGS reads, SRR16588090 and SRR16588091 for the Illumina Hi-C reads, SRR16351760 for the Illumina RNA-seq reads, SRR16351759 for the PacBio Iso-seq reads).

5) Where are the PacBio and ONT reads coming from? It seems that a description of the sequencing is missing. Which library preparation protocol was used? Which sequencing platform?
>>> We have included this information in Supplementary Table S1 and Supplementary File 1. Please see lines 116-117 and Supplementary Table S1, and Supplementary File 1.

6) The assembly is the core of this study thus more details about the actual assembly process are needed. Which parameters were used when running Nextdenovo? The authors might also want to compare their assembly results against an HiFi assembly. Apparently, this assembly was generated, but it is not clear how that was performed. Also, it would be good to compare the results against the results of other assemblers e.g. HiCanu (10.1101/gr.263566.120). Flye (https://doi.org/10.1038/s41587-019-0072-8) could be a good choice due to the high proportion of repetitive elements.
>>>The assembly script (with parameter) with the other results as GigaScience required (http://gigadb.org/site/guidegenomic) upon submitting the manuscript have been uploaded via FTP for reviewer, we also uploaded this file into figshare (https://doi.org/10.6084/m9.figshare.19165280.v10) and added this information in the revised text (please see lines 134-142). We also included the comparisons of different assemblers (please see lines 293-298 and Supplementary Table S6).

7) What is the definition of properly mapped reads? (line 145) While this is always desired, it is technically challenging to achieve this. Please explain how the mapping/filtering was performed.
>>>We have corrected the wrong expression. It is "properly paired" not "properly mapped". Please see lines 155-156.

8) Please specify the BLAST default parameters (line 155)
>>>We have included them. Please see lines 167-169.

9) The annotation could also be evaluated by a comparison against the banana genome hub information. How many of the typical Musaceae genes are present? When using BUSCO, it is important to specify the configuration in detail. Depending on these settings, completeness values can change substantially.
>>>The predicted gene sets were compared with the other Musa species, please see Figure S4. We also rephrased the sentences to provide more information, please see lines 208, 372-378. The script to run BUSCO is "busco --in mb_gap_closed_polished.fa --cpu 96 --out Mbe-busco --lineage_dataset /home/w/download/embryophyta_odb10 --mode geno --augustus_species arabidopsis". It is now provided in the assembly script, and with the other BUSCO result files ("Busco_full_table.tsv", "Busco_missing_list.tsv" and "Busco_short_summary.txt") uploaded via FTP for reviewer and also could be found in figshare (https://doi.org/10.6084/m9.figshare.19165280.v10).

10) The identification of gene families based on OrthoFinder is not an ideal approach. Gene families might be broken into multiple orthogroups or merged with non-members. At least for the MYBs, a dedicated annotation workflow is available (https://doi.org/10.1186/s12864-022-08452-5). Please check how the results compare against the OrthoFinder results to evaluate the performance of this analysis.
>>>We agree with the reviewer.
When examining MYB genes identified from MYB_annotator in OrthoFinder results (Supplementary Table S10), it revealed that each type of MYBs were assorted to different orthologous groups except those only one gene occurred. Then some more MYB alike genes identified in OrthoFinder. For example, in M. beccarii the MYB genes were found in 95 orthologous groups, while in these groups there were a total of 398 genes, far larger than 268 MYB genes identified from MYB_annotator. Therefore, more stringent workflow was

required for particular gene family analysis but not merely using OrthoFinder.
This is the reason why we used a complementary approach for the transcription factors using iTAK. In addition, we ran MYB_annotator. MYB_annotator identified similar amount of the MYB genes compared to iTAK results in M. beccarii and the others (Supplementary Table S10 and S11), and the amount from the former was generally slightly smaller than the latter. It makes us confident in the analyses computed for transcription factors, that complement Orthofinder for which there is easy alternate solution for all other gene families at large scale.
We update the manuscript with MYB results. Please see lines 201-203, 351-356. Supplementary Table S10-S12.

11) Why is all-versus-all BLASTP used if OrthoFinder already constructed groups? If this is for further refinement within orthogroups, it would be important to validate that there are no mis-assignments. Checking the synteny of gene pairs would be important. Apparently, such a synteny analysis was already performed, but the results need to be integrated.
>>> The all-versus-all BLASTP is required in the DupGen_finder pipeline. The pipeline was developed to identify different modes of duplicated gene pairs. The first step of the pipeline is to perform the all-versus-all local BLASTP to search all potential homologous gene pairs within genome. Then, the MCScanX (Wang et al. 2012) algorithm, which is based on MCscan (Wang et al. 2008), was utilized to identify the WGD-derived gene pairs. After excluding WGD-pairs from the whole set of homologous pairs (or BLASTP hits), the pipeline further determines the single-gene duplications, including tandem duplications (TD), proximal duplications (PD), transposed duplications (TRD), and dispersed duplications (DSD). In the text, we have removed the sentences ("…all-versus-all BLASTP …") that are actually not necessary as it is implicit with MCscan. Furthermore, because the same MCscan algorithm was used in the synteny analysis (please see section "Whole genome alignment and synteny analysis" of the text), part of DupGen_finder results (the WGD results) and synteny analysis results were similar. However, considering parameters and algorithms modifications, and different alignment search program used (BLASTP in DupGen_finder pipeline vs. LASTAL in synteny analysis (https://github.com/tanghaibao/jcvi/wiki/MCscan-(Python-version)) in two analyses, some different results might not be fully integrated in the current status.

References:
Tang HB, Wang XY, Bowers JE, Ming R, Alam M, Paterson AH. Unraveling ancient hexaploidy through multiply-aligned angiosperm gene maps. Genome Res. 2008,18(12):1944-1954.
Wang YP, Tang HB, DeBarry JD, Tan X, Li JP, Wang XY, et al. MCScanX: a toolkit for detection and evolutionary analysis of gene synteny and collinearity. Nucleic Acids Res. 2012,40:e49.

12) What is the definition of an enriched GO term? More details should be added about this analysis.
>>>We have included words to make that clear. Please see lines 222-226.

13) REVIGO lacks a reference (https://doi.org/10.1371/journal.pone.0021800).
>>>It actually has been cited in the previous section "Gene family and comparative genomics". Please see lines 226-227.

14) Were the NLR-Annotator results compared against the gene structures produced through the gene prediction process? NLR genes should be one of the best ways to perform the benchmarking of a high quality assembly.
>>>We have included this information. Please see lines 448-454 and Supplementary Table S34.

15) Predictions resulting from KmerGenie and GenomeScope differ by about 20%. Therefore, I would suggest to run some additional tools to narrow down the range of possible genome sizes. Here are some options: findGSE (https://doi.org/10.1093/bioinformatics/btx637), MGSE (https://doi.org/10.1101/607390), Gnodes (https://doi.org/10.1101/2022.05.13.491861), and gce (https://arxiv.org/abs/1308.2012).
>>>Thank you for the suggestion. We have included them and rephrased the sentence in the text accordingly. Except using Illumina short reads in the genome size estimation, we also include HiFi reads for the estimation. Please see lines 124-128, 288-291 and Supplementary Table S5.

16) It is not clear to me what this sentence means "KmerGenie estimated the optimal k-mer size for the

WGS short reads was 87". Is this the best k-mer for an assembly?
>>>Yes, it was automatically provided after performing KmerGenie program. We rephrased the sentence to make it clear. Please see line 287-288.

17) line 309: Was this check only performed based on the M. beccarii assembly or also by screening all reads belonging to that species? It is possible that a technical issue kept the region from being represented in the assembly. Checking the reads would be the more reliable approach.
>>>It was examined by both performing blasting against the assembly and mapping different types of WGS reads against assembly. The Egcen region was absent in M. beccarii. We rephrase the sentence to make it clearer. Please see lines 325-327.

18) MYBs are usually the most abundant group of TFs in plants. How do the numbers and sequences compare to the MYBs in Musa acuminata (https://doi.org/10.1371/journal.pone.0239275; https://doi.org/10.1101/2022.08.15.503939)? This is an available framework to ensure that the TF identification actually works.
>>>We have included this information. Please see answer to point 10, and additional lines 356-359, and Supplementary Table S10-12.

19) The purpose of performing GO and KEGG enrichment analyses is not clear to me. These analyses always produce some results, but what is their relevance? It would be important to look into complex pathways like the flavonoid biosynthesis to see HOW the species are different. Does a particular difference (presence/absence) correlate with the biology/phenotype of the respective species? I find it hard to believe that closely related plant species show a fundamental difference in the ratio of transcriptional control vs. translational control of their gene expression (suggested by the results). It is also unlikely that one species has significantly more membrane proteins than another species. There might be differences with respect to the specific proteins, but an overall differences does not seem plausible. Please check the results and drawn conclusions again.
>>>We have removed redundancy enrichment analyses including content of "helitron repetitive elements", Supplementary Table S9, S10, S13, S14, S19 and S20. The number of genes in the flavonoid biosynthesis is shown in Supplementary Table S9, we now include the pathways picture (please see Supplementary Figure S15).
However, we did not conduct experiments to compare the relationships of gene numbers and particular flavonoid biosynthesis related traits, nor gene expression experiments to find the relationship, we therefore have not drawn some conclusion like those in the text. Based on current results, some experiments could be conducted in the future. We have rephrased the sentences (removing the word of "over-expression" in sentence, lines 574-576) and included new sentences to make it clear (lines 584-587).

20) Is there a BGC that is specific to M. beccarii and could explain a pathogen response phenotype? BGCs are often associated with evolutionary novel defense components.
>>>We agree with reviewer. According to Table 2, our study did not find M. beccarii specific BGCs, future studies are needed to confirm these BGCs. We added some words to explain that. Please see line 632.

21) The authors investigated gene family expansion with focus on the TFs and looked for mechanisms to explain duplications. I would recommend to screen the assembly for duplicated regions that have been reported in close relatives before e.g. large segmental duplication on chr2 (https://doi.org/10.1534/g3.119.400847). Finding such duplications would support the statement about the TF duplications.
>>>We have included the analyses the reviewer suggested. The results show no large segmental nor recent duplications occurred in M. beccarii (a diploid species). Please see lines 509-516 and Supplementary Figure S13 and S14.

22) Is the large variation in chromosome sizes due to biological differences or could these be caused by technical artifacts. How does the fraction of missing/unassigned sequences compare between the species? For example, all unassigned sequences in another species could belong to the largest chromosome. The statement about the variation of chromosome sizes would required assemblies of equal quality in all species.
>>> The assembly size of M. beccarii is among the estimations of different programs (we run different

genome size estimating as suggested by question 15 from the reviewer, and please see the results in Supplementary Table S5). It currently unknown how different genome sizes among Musaceae species result in different biological functions. We then run structure variation analysis for M. beccarii with M. acuminata version 4, which is the most complete genome in Musaceae, it shows that the increased sequences in M. beccarii come from its different chromosomes. We included new sentences and table for the answers. Please see lines 478-482 and Supplementary Table S35.

23) It seems that the statement about the largest genome size (570Mbp, M. beccarii) is contradicted by some of the following sentences. The authors might want to check this again. It might be necessary to take the ploidy into consideration when comparing genome sizes.
>>>The assembly size of M. beccarii is among the estimations of different programs, please see above question 15. The "contradiction" sentences following the "genome size (assembly ≈ 570 Mbp…." are stating the flow cytometry results, which shows that the genome size estimation using flow cytometry may overestimate the genome size of M. beccarii, and the other Musa species, too. We rephrase the sentence to make it clear. Please see lines 470-471.
Furthermore, according to the Karyological observation of Häkkinen et al. (2007) and reference therein, M. beccarii is a diploid species. Following the method of Busche et al. (2020), by mapping the Illumnia WGS reads against the M. beccarii assembly, the allele frequencies of SNV (single nucleotide variant) obtained showing a single peak frequency of 0.5 (please see Supplementary Figure S14), indicating a diploid species of M. beccarii. The large genome size could be attributed to the repetitive sequences as shown in "Genome size" section of Discussion.

Reference:
Busche M, Pucker B, Viehöver P, Weisshaar B, Stracke R. Genome sequencing of Musa acuminata Dwarf Cavendish reveals a duplication of a large segment of Chromosome 2. G3-Genes Genom. Genet. 2020,10 (1):37-42.
Häkkinen M, Suchuánková P, Doleželová M, Hřibová E, Doležel J. Karyological observation in Musa beccarii var. hottana (Musaceae). Acta Phytotax Geobot. 2007,58(2/3):112-118.

24) The flavonoid biosynthesis is a well studied system of many branching reactions (https://doi.org/10.1104/pp.126.2.485; https://doi.org/10.3390/plants9091103, Fig. 1). Differences in single genes could already explain differences between species. Therefore, it is surprising that large differences picked up by enrichment analyses are reported here. Flavonoids are a large group of > 10k different substances. According to the descriptions in the introduction, M. beccarii might be enriched with anthocyanins (red pigments). ABC transporters are part of the flavonoid biosynthesis (https://doi.org/10.3390%2Fijms140714950; https://doi.org/10.3390/plants11070963) thus a connection between duplications could make sense. Again, a single gene difference could cause a huge phenotypical difference. This does not require a systematic increase of the entire gene family.
>>>We have included new sentences to address these and added the four literature citations the reviewer mentioned. The Supplementary Table S9 summarized the gene numbers of different species in the flavonoid biosynthesis which is obtained by functional annotation using the same procedures. In current study, we sampled a M. beccarii seedling and only performed transcriptome sequencing using its leaves. So we did not perform the comparative transcriptome analysis with different tissues and such analysis among species. We now include gene number comparison of different species in anthocyanin biosynthesis and sentences related to anthocyanin studies. Please see lines 568-571, 580-582, 588-596 and Supplementary Table S9.

Minor comments:
line 80: are polyploid triploids
>>> We have removed the "polyploidy". Please see line 81.

line 305: "After removing sequence length short than 1,000 bp" ... please rephrase.
>>>We have rephrased the sentences. Please see lines 323-324.

line 588: -predator > herbivore?
>>>We have replaced "predator" with "herbivore". Please see line 611.

line 651: is > are
>>>We have corrected that. Please see line 674.

Reviewer: 2
Wang et al sequenced and assembled a chromosome-level genome assembly for Musa beccarii, a banana species with high ornamental value. The genome assembly is of high completness and continuity. They described the gene strucutre and function annotation, and repeat annotations. They also compared the genome with relative species from the family Musaceae, including phylogenetic construction, gene family clustering and gene family size evolution, genomic synteny and whole genome duplication, chromosome rearrangement and ancestral genome reconstruction. They also focused on the gene families with specific function, such as NBS-LRR genes and biosyntheic gene clusters. Overall, this is a good paper on genome sequencing, with my recommendation of publication on GigaScience. Before publishing, a few issues need to be addressed.

1) The English writing must be significantly improved. I was confused with not a few sentences.
>>> The manuscript has now been carefully edited by Prof. Pat (J.S.) Heslop-Harrison (one of the authors), a native English speaker. The corrected parts are marked in red.

2) The Discussion section may be shortened. Some contents that are now placed in Discussion should be moved to Results. For examples, Lines 444-446, Lines 478-481, Lines 494-498,
>>>We have removed lines 444-446 and its following sentences, removed paragraphs that include the lines 478-481 and 494-498 to shorten the Discussion section.

3) Lines 638-639. "Dupgen_finder ..... in the genome." Why you mentioned this at this place? I can not see the meaning of stating some DNA repair genes are DSD.
>>>We have removed the sentence.

4) All figures excluding Fig.1 and Fig.3 are unreadable, due to low resolution.
>>>We now provided figures with high resolution. Please click the pictures, download and read them.

Miscellaneous:
Because there are supplementary tables, figures and references removed and new ones added, the order of supplementary tables, figures and references in the revised manuscript are changed. We also update and correct some format errors in some references.

Please let us know if there are any additional concerns about our manuscript after we have offered these corrections and responses, as we are happy to address any continued issues or proposed changes to the manuscript. Thank you for your time.

Sincerely yours,
Xue-Jun Ge
South China Botanical Garden
Chinese Academy of Sciences
Guangzhou, 510650
China

Close
