# Supplementary material for: Genome assembly of Musa beccarii shows extensive chromosomal rearrangements and genome expansion during evolution of Musaceae genomes
Source: Gigascience. 2023 Feb 21;12:giad005. doi: 10.1093/gigascience/giad005 (PMC9941839; doi:10.1093/gigascience/giad005)
Supplement: giad005_Response_to_Reviewer_Comments_Revision_1 [file giad005_response_to_reviewer_comments_revision_1.pdf]

Author's Response To Reviewer Comments

Close

November 19, 2022
GigaScience

Dear editor,
Thank you very much for your decision letter about our manuscript entitled “Genome assembly of Musa beccarii shows extensive chromosomal rearrangements and genome expansion during evolution of Musaceae genomes”(No. GIGA-D-22-00219R1), including the comments.
We are now sending our responses to comments with our revised manuscript (both tracked and clean versions). All the modified parts in the revised manuscript are marked in red.
Our specific responses are as follows:

Reviewer(s)' Comments to Author:

Reviewer #1:
The authors addressed almost all of my comments very well. I see an overall improvement of the manuscript. There are only a few remaining points:

1) "assembled genome" > "genome sequence"
>>>We have corrected them throughout the text.

3) Unfortunately, this was not clarified through the changes and is not correct yet:
"PacBio full-length RNA transcripts sequencing" > "PacBio full-length cDNA sequencing"
"short-read RNA transcripts sequencing" > "short-read cDNA fragment sequencing"
>>>We have corrected them. Please see lines 127-128.

4) What about the growth conditions (temperature, humidity, soil, ...)? As many details as possible should be provided to make the data sets suitable for re-use.
>>>We have enlarge the paragraph to include this information. Please see lines 109-119.

5) It would be important to include additional information about the ONT basecalling. I would also recommend to include all these sequencing method descriptions in the main document and not in a supplementary file. There are different font sizes/types used in this document (can be ignored if moved to the main document).
>>>We have included the ONT basecalling information, please see lines 185-187. The methods are now in the text and the supplymentary file 1 is deleted, please see lines 129-172.

after examination, the RNA was synthesized into cDNA > after examination, the RNA was reverse transcribed into cDNA
>>>We have rephrased that. Please see line 166.

7) I am not able to find the expression "properly mapped" in that sentence. It seems that only the percentage of mapped reads was considered. Was no filtering for multi-mapped reads and no filtering based on alignment scores applied? This sentence could benefit from some additional explanations.
>>>We removed “properly mapped” from our initial version of the manuscript because of its wrong expression. The followed question that the reviewer mentioned could not be obtained by the results from “flagstat” command of SAMtools. We now run SQUAT (Yang et al. 2019), a sequence assembly quality assessment tool, to include this information. Through the mapped reads, SQUAT categorizes reads into groups of uniquely mapped reads, multiply mapped, unmapped reads. For uniquely mapped reads, it further categorizes them into perfectly matched, with substitutions, containing clips, and the others. Please

see lines 213-220, 379-384.

Reference:

Yang L-A, Chang Y-J, Chen S-H, Lin C-Y. Ho J-M. SQUAT: a Sequencing Quality Assessment Tool for data quality assessments of genome assemblies. BMC Genom. 2019,19(Suppl 9):238

9) Thanks for the update. Unfortunately, I cannot find the BUSCO config file there. It would be interesting to know the e-value cutoffs and the number of genes to consider etc. These settings can influence the completeness score. My initial impression was that BUSCO was used to assess the completeness of the annotation. However, the log file suggest that BUSCO was run in the genome mode i.e. ignored the annotation and started with a gene prediction from scratch.
>>>We used BUSCO v5.2.2 and installed it via CONDA. When running the program, we did not use a config file and the program was then run under its default parameters setting. The config file is also not generated after running the program, but the settings were shown in a running log file. The e-value in the default setting is 0.001. We now upload the log file for reviewing (please see https://doi.org/10.6084/m9.figshare.19165280.v13).
We run BUSCO in two models, genome and protein model ("--mode prot" in which we used longest transcripts, 39,112 protein sequences). In the text, we provide the BUSCO gene model result (please see lines 419-421), but not indicated it in "Materials and Methods", we now include the information in the text. Please see lines 248-250. We also upload the gene model results into the website of "figshare" for reviewing (please see https://doi.org/10.6084/m9.figshare.19165280.v13).

11) The last part about LASTAL is not clear, but it seems that the authors have considered synteny.
>>>It should be the program "LAST" (https://gitlab.com/mcfrith/last). The command usage is "lastal …", we mislead that.

12) This addition is helpful. What was the specific p-value cutoff or any other cutoff used to define 'significantly enriched'?
>>>We have included this information. Please see lines 291-292.

X1) Supplementary Fig1: Illustrating the process is very important, but I have some questions about some aspects:
a) Why was the removal of duplicates performed multiple times in the HiFi branch of the process? Duplicates removed at the contig level should not become an issue after the scaffolding.
>>>Thank you. We actually did not compare the scaffolding results between removing and non-removing the duplications in the contigs. In our study, we used Pseudohaploid (https://github.com/schatzlab/pseudohaploid) and Purge_Dups (Guan et al. 2020) (Please see lines 201-203) to reduce the duplications caused by heterozygosity in both Nanopore and Hifi read based assemblies (please see Figure S1). According to Pseudohaploid manual and Guan et al. (2020), removing heterozygous duplications should not only improve scaffolding but also gene annotation. However, because such duplication could not be completely removed (please see Figure 1 in Guan et al. 2020 and Purge_Dups website discussion issue https://github.com/dfguan/purge_dups/issues/74), we therefore run the deduplication multiple times (please see the "Mbe_genome_assembly_srcipt.txt" file uploaded to https://doi.org/10.6084/m9.figshare.19165280.v13), which is also supported in the Purge_Dups website discussion issue (https://github.com/dfguan/purge_dups/issues/66). We now add words to make the deduplication step clear, please see line 203.

Reference:

Guan, DF, McCarthy SA, Wood J, Howe K, Wang YD. Identifying and removing haplotypic duplication in primary genome assemblies. Bioinformatics. 2020,36:2896-2898.

b) Combining ONT and HiFi assembled contigs does not make sense to me. Most regions must be present in both assembly. The motivation for this approach could be explained. Was it considered to run a combined assembly with both read sets?
>>>We agree with the reviewer. In the process, we used program RagTag (https://github.com/malonge/RagTag) to correct the Nanopore assembly using HiFi assembly (please see step 17 in "Mbe_genome_assembly_srcipt.txt" uploaded to

https://doi.org/10.6084/m9.figshare.19165280.v13). The command is "ragtag.py correct …". The "correct" module in RagTag program only breaks the query sequence at points of putative misassembly but not merge/combine two assemblies (please see https://github.com/malonge/RagTag/wiki/correct).

X2) While it is possible to understand the manuscript, an improvement of the writing could help. This is not about removing typos or grammatical errors, but to improve the readability.
>>>We have now improved the writing by English language editing company TopEdit (www.topeditsci.com). All revised parts are marked in red.

Minor comment:
line453: compete > complete
>>>We have corrected that. Please see line 533.

--
Please also take a moment to check our website at for any additional comments that were saved as attachments. Please note that as GigaScience has a policy of open peer review, you will be able to see the names of the reviewers.
>>>We find no attached file(s) that contain additional comments.

Miscellaneous:
Because there are new references added, the order of references in the revised manuscript are changed.

Please let us know if there are any additional concerns about our manuscript after we have offered these corrections and responses, as we are happy to address any continued issues or proposed changes to the manuscript. Thank you for your time.

Sincerely yours,

Xue-Jun Ge
South China Botanical Garden
Chinese Academy of Sciences
Guangzhou, 510650
China

Close
