# Supplementary material for: Genome assembly of Musa beccarii shows extensive chromosomal rearrangements and genome expansion during evolution of Musaceae genomes
Source: Gigascience. 2023 Feb 21;12:giad005. doi: 10.1093/gigascience/giad005 (PMC9941839; doi:10.1093/gigascience/giad005)
Supplement: giad005_Reviewer_1_Report_Original_Submission — Boas Pucker -- 9/13/2022 Reviewed [file giad005_reviewer_1_report_original_submission.pdf]

Reviewer Report

Title: Genome assembly of Musa beccarii shows extensive chromosomal rearrangements and genome expansion during evolution of Musaceae genomes

Version: Original Submission **Date:** 9/13/2022

Reviewer name: Boas Pucker

Reviewer Comments to Author:

Wang et al. describe the genome sequence of Musa beccarii. The assembly is presented and compared against existing genome sequences. This additional genome sequence provides further insights into the evolution of Musaceae. All data sets were submitted to the corresponding repositories and will be shared via the banana genome hub. This work is certainly of interest to the community and the availability of all data sets will facilitate reuse in future studies. While the data set is of high quality, there are some (technical) issues that should be addressed. For example, the statements about the genome size should be checked and additional evidence for the contraction/expansion event would be helpful. Please find a list of specific comments below.1) There is a difference between genome (DNA) and a genome sequence (information). This is particularly relevant when the genome size does not match the assembly size. Please adjust the use of both terms throughout the manuscript.2) Do the authors have an approval from Malaysia to use this material for research and to publish genomic and transcriptomic data sets?3) line 113: PacBio is not able to sequence RNAs. ONT is the only technology for high throughout RNA sequencing. What the authors are trying to describe is probably a cDNA sequencing run (with HiFi?). The short reads are most likely also derived from cDNAs.4) Details of the conditions and time points of RNA extraction need to be added. Almost nothing is written about the samples used for the sequencing experiments. Please make sure to update the metadata (growth condition, plant part, developmental stage, ...) accordingly.5) Where are the PacBio and ONT reads coming from? It seems that a description of the sequencing is missing. Which library preparation protocol was used? Which sequencing platform?6) The assembly is the core of this study thus more details about the actual assembly process are needed. Which parameters were used when running Nextdenovo? The authors might also want to compare their assembly results against an HiFi assembly. Apparently, this assembly was generated, but it is not clear how that was performed. Also, it would be good to compare the results against the results of other assemblers e.g. HiCanu (10.1101/gr.263566.120). Flye (https://doi.org/10.1038/s41587-019-0072-8) could be a good choice due to the high proportion of repetitive elements.7) What is the definition of properly mapped reads? (line 145) While this is always desired, it is technically challenging to achieve this. Please explain how the mapping/filtering was performed.8) Please specify the BLAST default parameters (line 155)9) The annotation could also be evaluated by a comparison against the banana genome hub information. How many of the typical Musaceae genes are present? When using BUSCO, it is important to specify the configuration in detail. Depending on these settings, completeness values can change substantially.10) The identification of gene families based on OrthoFinder is not an ideal approach. Gene families might be broken into multiple orthogroups or merged with non-members. At least for the MYBs, a dedicated

annotation workflow is available (https://doi.org/10.1186/s12864-022-08452-5). Please check how the results compare against the OrthoFinder results to evaluate the performance of this analysis.11) Why is all-versus-all BLASTP used if OrthoFinder already constructed groups? If this is for further refinement within orthogroups, it would be important to validate that there are no mis-assignments. Checking the synteny of gene pairs would be important. Apparently, such a synteny analysis was already performed, but the results need to be integrated.12) What is the definition of an enriched GO term? More details should be added about this analysis.13) REVIGO lacks a reference (https://doi.org/10.1371/journal.pone.0021800).14) Were the NLR-Annotator results compared against the gene structures produced through the gene prediction process? NLR genes should be one of the best ways to perform the benchmarking of a high quality assembly.15) Predictions resulting from KmerGenie and GenomeScope differ by about 20%. Therefore, I would suggest to run some additional tools to narrow down the range of possible genome sizes. Here are some options: findGSE (https://doi.org/10.1093/bioinformatics/btx637), MGSE (https://doi.org/10.1101/607390), Gnodes (https://doi.org/10.1101/2022.05.13.491861), and gce (https://arxiv.org/abs/1308.2012).16) It is not clear to me what this sentence means "KmerGenie estimated the optimal k-mer size for the WGS short reads was 87". Is this the best k-mer for an assembly?17) line 309: Was this check only performed based on the M. beccarii assembly or also by screening all reads belonging to that species? It is possible that a technical issue kept the region from being represented in the assembly. Checking the reads would be the more reliable approach.18) MYBs are usually the most abundant group of TFs in plants. How do the numbers and sequences compare to the MYBs in Musa acuminata (https://doi.org/10.1371/journal.pone.0239275; https://doi.org/10.1101/2022.08.15.503939)? This is an available framework to ensure that the TF identification actually works.19) The purpose of performing GO and KEGG enrichment analyses is not clear to me. These analyses always produce some results, but what is their relevance? It would be important to look into complex pathways like the flavonoid biosynthesis to see HOW the species are different. Does a particular difference (presence/absence) correlate with the biology/phenotype of the respective species? I find it hard to believe that closely related plant species show a fundamental difference in the ratio of transcriptional control vs. translational control of their gene expression (suggested by the results). It is also unlikely that one species has significantly more membrane proteins than another species. There might be differences with respect to the specific proteins, but an overall differences does not seem plausible. Please check the results and drawn conclusions again.20) Is there a BGC that is specific to M. beccarii and could explain a pathogen response phenotype? BGCs are often associated with evolutionary novel defense components.21) The authors investigated gene family expansion with focus on the TFs and looked for mechanisms to explain duplications. I would recommend to screen the assembly for duplicated regions that have been reported in close relatives before e.g. large segmental duplication on chr2 (https://doi.org/10.1534/g3.119.400847). Finding such duplications would support the statement about the TF duplications.22) Is the large variation in chromosome sizes due to biological differences or could these be caused by technical artifacts. How does the fraction of missing/unassigned sequences compare between the species? For example, all unassigned sequences in another species could belong to the largest chromosome. The statement about the variation of chromosome sizes would required assemblies of equal quality in all species.23) It seems that the statement about the largest genome size (570Mbp, M. beccarii) is contradicted by some of the following sentences. The authors might want to

check this again. It might be necessary to take the ploidy into consideration when comparing genome sizes.24) The flavonoid biosynthesis is a well studied system of many branching reactions (https://doi.org/10.1104/pp.126.2.485; https://doi.org/10.3390/plants9091103, Fig. 1). Differences in single genes could already explain differences between species. Therefore, it is surprising that large differences picked up by enrichment analyses are reported here. Flavonoids are a large group of >10k different substances. According to the descriptions in the introduction, M. beccarii might be enriched with anthocyanins (red pigments). ABC transporters are part of the flavonoid biosynthesis (https://doi.org/10.3390%2Fijms140714950; https://doi.org/10.3390/plants11070963) thus a connection between duplications could make sense. Again, a single gene difference could cause a huge phenotypical difference. This does not require a systematic increase of the entire gene family.Minor comments:line 80: are polyploid triploidsline 305: "After removing sequence length short than 1,000 bp" ... please rephrase.line 588: -predator > herbivor?line 651: is > are

Level of Interest

Please indicate how interesting you found the manuscript: Choose an item.

Quality of Written English

Please indicate the quality of language in the manuscript: Choose an item.

Declaration of Competing Interests

Please complete a declaration of competing interests, considering the following questions:

- Have you in the past five years received reimbursements, fees, funding, or salary from an organisation that may in any way gain or lose financially from the publication of this manuscript, either now or in the future?
- Do you hold any stocks or shares in an organisation that may in any way gain or lose financially from the publication of this manuscript, either now or in the future?
- Do you hold or are you currently applying for any patents relating to the content of the manuscript?
- Have you received reimbursements, fees, funding, or salary from an organization that holds or has applied for patents relating to the content of the manuscript?
- Do you have any other financial competing interests?
- Do you have any non-financial competing interests in relation to this paper?

If you can answer no to all of the above, write 'I declare that I have no competing interests' below. If your reply is yes to any, please give details below.

I declare that I have no competing interests

I agree to the open peer review policy of the journal. I understand that my name will be included on my report to the authors and, if the manuscript is accepted for publication, my named report including any attachments I upload will be posted on the website along with the authors' responses. I agree for my report to be made available under an Open Access Creative Commons CC-BY license (http://creativecommons.org/licenses/by/4.0/). I understand that any comments which I do not wish to be included in my named report can be included as confidential comments to the editors, which will not be published.

Choose an item.

To further support our reviewers, we have joined with Publons, where you can gain additional credit to further highlight your hard work (see: https://publons.com/journal/530/gigascience). On publication of this paper, your review will be automatically added to Publons, you can then choose whether or not to claim your Publons credit. I understand this statement.

Yes Choose an item.
