# Supplementary material for: Genome assembly of Musa beccarii shows extensive chromosomal rearrangements and genome expansion during evolution of Musaceae genomes
Source: Gigascience. 2023 Feb 21;12:giad005. doi: 10.1093/gigascience/giad005 (PMC9941839; doi:10.1093/gigascience/giad005)
Supplement: giad005_Reviewer_1_Report_Revision_1 — Boas Pucker -- 11/28/2022 Reviewed [file giad005_reviewer_1_report_revision_1.pdf]

Reviewer Report

Title: Genome assembly of Musa beccarii shows extensive chromosomal rearrangements and genome expansion during evolution of Musaceae genomes

Version: Revision 1 **Date:** 11/28/2022

Reviewer name: Boas Pucker

Reviewer Comments to Author:

The authors addressed almost all of my comments very well. I see an overall improvement of the manuscript. There are only a few remaining points:1) "assembled genome" > "genome sequence"3) Unfortunately, this was not clarified through the changes and is not correct yet:"PacBio full-length RNA transcripts sequencing" > "PacBio full-length cDNA sequencing""short-read RNA transcripts sequencing" > "short-read cDNA fragment sequencing"4) What about the growth conditions (temperature, humidity, soil, ...)? As many details as possible should be provided to make the data sets suitable for re-use.5) It would be important to include additional information about the ONT basecalling. I would also recommend to include all these sequencing method descriptions in the main document and not in a supplementary file. There are different font sizes/types used in this document (can be ignored if moved to the main document).after examination, the RNA was synthesized into cDNA > after examination, the RNA was reverse transcribed into cDNA7) I am not able to find the expression "properly mapped" in that sentence. It seems that only the percentage of mapped reads was considered. Was no filtering for multi-mapped reads and no filtering based on alignment scores applied? This sentence could benefit from some additional explanations.9) Thanks for the update. Unfortunately, I cannot find the BUSCO config file there. It would be interesting to know the e-value cutoffs and the number of genes to consider etc. These settings can influence the completeness score. My initial impression was that BUSCO was used to assess the completeness of the annotation. However, the log file suggest that BUSCO was run in the genome mode i.e. ignored the annotation and started with a gene prediction from scratch.11) The last part about LASTAL is not clear, but it seems that the authors have considered synteny.12) This addition is helpful. What was the specific p-value cutoff or any other cutoff used to define 'significantly enriched'?X1) Supplementary Fig1: Illustrating the process is very important, but I have some questions about some aspects:a) Why was the removal of duplicates performed multiple times in the HiFi branch of the process? Duplicates removed at the contig level should not become an issue after the scaffolding.b) Combining ONT and HiFi assembled contigs does not make sense to me. Most regions must be present in both assembly. The motivation for this approach could be explained. Was it considered to run a combined assembly with both read sets?X2) While it is possible to understand the manuscript, an improvement of the writing could help. This is not about removing typos or grammatical errors, but to improve the readability.Minor comment:line453: compete > complete

Level of Interest

Please indicate how interesting you found the manuscript: Choose an item.

Quality of Written English

Please indicate the quality of language in the manuscript: Choose an item.

Declaration of Competing Interests

Please complete a declaration of competing interests, considering the following questions:

- Have you in the past five years received reimbursements, fees, funding, or salary from an organisation that may in any way gain or lose financially from the publication of this manuscript, either now or in the future?
- Do you hold any stocks or shares in an organisation that may in any way gain or lose financially from the publication of this manuscript, either now or in the future?
- Do you hold or are you currently applying for any patents relating to the content of the manuscript?
- Have you received reimbursements, fees, funding, or salary from an organization that holds or has applied for patents relating to the content of the manuscript?
- Do you have any other financial competing interests?
- Do you have any non-financial competing interests in relation to this paper?

If you can answer no to all of the above, write 'I declare that I have no competing interests' below. If your reply is yes to any, please give details below.

I declare that I have no competing interests

I agree to the open peer review policy of the journal. I understand that my name will be included on my report to the authors and, if the manuscript is accepted for publication, my named report including any attachments I upload will be posted on the website along with the authors' responses. I agree for my report to be made available under an Open Access Creative Commons CC-BY license (http://creativecommons.org/licenses/by/4.0/). I understand that any comments which I do not wish to be included in my named report can be included as confidential comments to the editors, which will not be published.

Choose an item.

To further support our reviewers, we have joined with Publons, where you can gain additional credit to further highlight your hard work (see: https://publons.com/journal/530/gigascience). On publication of this paper, your review will be automatically added to Publons, you can then choose whether or not to claim your Publons credit. I understand this statement.

Yes Choose an item.
