# Supplementary material for: Genome assembly of Musa beccarii shows extensive chromosomal rearrangements and genome expansion during evolution of Musaceae genomes
Source: Gigascience. 2023 Feb 21;12:giad005. doi: 10.1093/gigascience/giad005 (PMC9941839; doi:10.1093/gigascience/giad005)
Supplement: giad005_Reviewer_2_Report_Original_Submission — Shuhua Shi -- 10/1/2022 Reviewed [file giad005_reviewer_2_report_original_submission.pdf]

**Reviewer Report**

**Title: Genome assembly of Musa beccarii shows extensive chromosomal rearrangements and genome expansion during evolution of Musaceae genomes**

**Version: Original Submission** **Date:** 10/1/2022

**Reviewer name: Shuhua Shi**

**Reviewer Comments to Author:**

Wang et al sequenced and assembled a chromosome-level genome assembly for Musa beccarii, a banana species with high ornamental value. The genome assembly is of high completness and continuity. They described the gene strucutre and function annotation, and repeat annotations. They also compared the genome with relative species from the family Musaceae, including phylogenetic construction, gene family clustering and gene family size evolution, genomic synteny and whole genome duplication, chromosome rearrangement and ancestral genome reconstruction. They also focused on the gene families with specific function, such as NBS-LRR genes and biosyntheic gene clusters. Overall, this is a good paper on genome sequencing, with my recommendation of publication on GigaScience. Before publishing, a few issues need to be addressed.1. The English writing must be significantly improved. I was confused with not a few sentences.2. The Discussion section may be shortened. Some contents that are now placed in Discussion should be moved to Results. For examples, Lines 444-446, Lines 478-481, Lines 494-498,3. Lines 638-639. "Dupgen_finder ..... in the genome." Why you mentioned this at this place? I can not see the meaning of stating some DNA repair genes are DSD.4. All figures excluding Fig.1 and Fig.3 are unreadable, due to low resolution.

**Level of Interest**

Please indicate how interesting you found the manuscript: Choose an item.

**Quality of Written English**

Please indicate the quality of language in the manuscript: Choose an item.

**Declaration of Competing Interests**

Please complete a declaration of competing interests, considering the following questions:

- Have you in the past five years received reimbursements, fees, funding, or salary from an organisation that may in any way gain or lose financially from the publication of this manuscript, either now or in the future?
- Do you hold any stocks or shares in an organisation that may in any way gain or lose financially from the publication of this manuscript, either now or in the future?

- Do you hold or are you currently applying for any patents relating to the content of the manuscript?
- Have you received reimbursements, fees, funding, or salary from an organization that holds or has applied for patents relating to the content of the manuscript?
- Do you have any other financial competing interests?
- Do you have any non-financial competing interests in relation to this paper?

If you can answer no to all of the above, write 'I declare that I have no competing interests' below. If your reply is yes to any, please give details below.

I declare that I have no competing interests

I agree to the open peer review policy of the journal. I understand that my name will be included on my report to the authors and, if the manuscript is accepted for publication, my named report including any attachments I upload will be posted on the website along with the authors' responses. I agree for my report to be made available under an Open Access Creative Commons CC-BY license (http://creativecommons.org/licenses/by/4.0/). I understand that any comments which I do not wish to be included in my named report can be included as confidential comments to the editors, which will not be published.

Choose an item.

To further support our reviewers, we have joined with Publons, where you can gain additional credit to further highlight your hard work (see: https://publons.com/journal/530/gigascience). On publication of this paper, your review will be automatically added to Publons, you can then choose whether or not to claim your Publons credit. I understand this statement.

Yes Choose an item.
